# Single Atom Engineering for Electrocatalysis: Fundamentals and Applications

**DOI:** 10.1021/acscatal.4c08027

**Published:** 2025-06-20

**Authors:** Mario Urso, Xiaohui Ju, Radhika Nittoor-Veedu, Hyesung Lee, Dagmar Zaoralová, Michal Otyepka, Martin Pumera

**Affiliations:** † Dipartimento di Fisica e Astronomia “Ettore Majorana”, Università di Catania, Via Santa Sofia 64, 95123 Catania, Italy; ‡ CNR-IMM, Via Santa Sofia 64, 95123 Catania, Italy; § Future Energy and Innovation Laboratory, Central European Institute of Technology, 48274 Brno University of Technology, Purkyňova 123, 612 00 Brno, Czech Republic; ∥ Quantum Materials Laboratory, 3D Printing & Innovation Hub, Center for Nanorobotics and Machine Intelligence, Department of Chemistry and Chemical Biology, Mendel University in Brno, Zemědělská 1, 613 00 Brno, Czech Republic; ⊥ IT4Innovations, VŠB − Technical University of Ostrava, 17. listopadu 2172/15, 708 00 Ostrava, Czech Republic; # Regional Centre of Advanced Technologies and Materials, Czech Advanced Technology and Research Institute (CATRIN), Palacký University Olomouc, Šlechtitelů 241/27, 783 71 Olomouc, Czech Republic; ∇ Advanced Nanorobots & Multiscale Robotics Laboratory, Faculty of Electrical Engineering and Computer Science, VŠB − Technical University of Ostrava, 17. listopadu 2172/15, 708 00 Ostrava, Czech Republic

**Keywords:** single-atom catalysis, catalyst engineering, atomically dispersed sites, metal−support interaction, computational modeling, electrocatalytic reactions

## Abstract

The global transition to sustainable energy production revolves around innovations in electrocatalysis, the cornerstone of energy conversion technologies. Over the years, catalysts have evolved from bulk materials to nanoparticles (NPs) and nanoclusters (NCs), culminating in single-atom catalysts (SACs), which represent the peak of catalyst engineering. SACs have revolutionized electrocatalytic processes by maximizing atom efficiency and offering tunable electronic properties, lowering the energy barrier associated with the absorption and desorption of key reaction intermediates, thus promoting specific reaction pathways. This review delves into the synthesis, characterization, and theoretical modeling of SACs, offering a comprehensive analysis of state-of-the-art methodologies. It highlights recent breakthroughs in diverse electrocatalytic reactions, including the hydrogen evolution reaction (HER) and oxygen evolution reaction (OER) in water splitting, the oxygen reduction reaction (ORR) for Zn–air batteries and fuel cells, the CO_2_ reduction reaction (CO_2_RR), and green ammonia synthesis. The discussion emphasizes the unique mechanisms that drive the exceptional performance of SACs, shedding light on their unparalleled activity, selectivity, and stability. By integrating experimental insights with computational advances, this work outlines a path for the rational design of next-generation SACs tailored to a broad spectrum of electrocatalytic applications. While summarizing the current landscape of electrocatalysis by SACs, it also outlines future directions to address the energy challenges of tomorrow, serving as a valuable resource for advancing the field.

## Graphical abstract

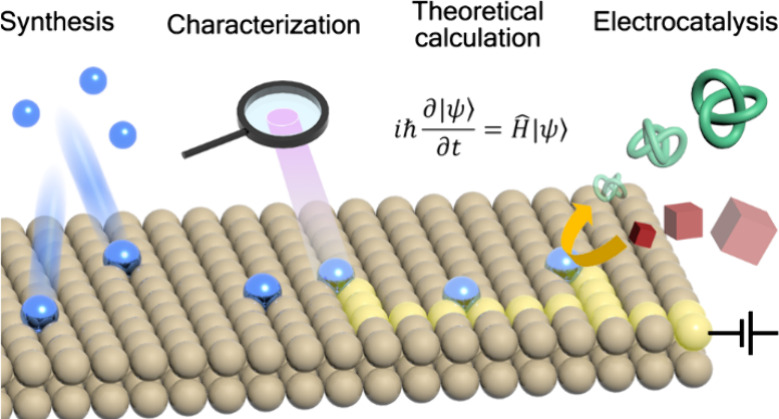

## 1 Fundamentals of Single-Atom Electrocatalysis: Beyond the Bulk and Nanoscale

The increasing energy demand and the rising concerns about climate change have driven the urgent need to transition from fossil fuels to sustainable and renewable energy sources. In this context, electrochemical processes behind energy conversion technologies, such as green hydrogen production by water splitting reaction in water electrolyzers,^1–3^ the oxygen reduction reaction (ORR) in metal–air batteries and fuel cells,^4–6^ the CO_2_ reduction reaction (CO_2_RR),^7,8^ and green ammonia (NH_3_) synthesis,^9,10^ have emerged as promising solutions to solve this pressing challenge. The efficiency of these systems is determined by the electrocatalytic properties of the active materials, which have to be further developed to lower the energy barriers associated with the reactions, accelerate their kinetics, and boost the overall efficiency.^11^ At the same time, it is imperative to reduce the cost and enhance the sustainability of the catalysts and their preparation methods.^12^ Indeed, among the best-performing catalysts are expensive metals, such as Pt, which excels in catalyzing the hydrogen evolution reaction (HER) in water electrolysis.^13^

With the advent of nanotechnology, the performance of nanostructured metals has significantly improved compared to their bulk counterpart. Specifically, reducing the size of metals to nanoparticles (NPs) has demonstrated improvement due to new effects emerging at the nanoscale, associated with the increased surface area of these materials and quantum phenomena.^14,15^ A further decrease in size exposes a greater number of undercoordinated surface atoms and alters the electronic structure of the catalyst, enhancing its electrocatalytic properties (Figure 1).^16^ Indeed, the reduction of NPs to smaller nanoclusters (NCs) represented another advancement in catalyst design.^17^ Following this strategy, it is not surprising that successive efforts have been devoted to further downscale the size of the metal until obtaining an atomically sized catalyst. The first example of this catalyst was introduced in the study by Zhang et al, who synthesized Pt single atoms (SAs) on an FeO_*x*_ substrate obtaining unprecedented performance in CO oxidation.^18^

**Figure 1:**
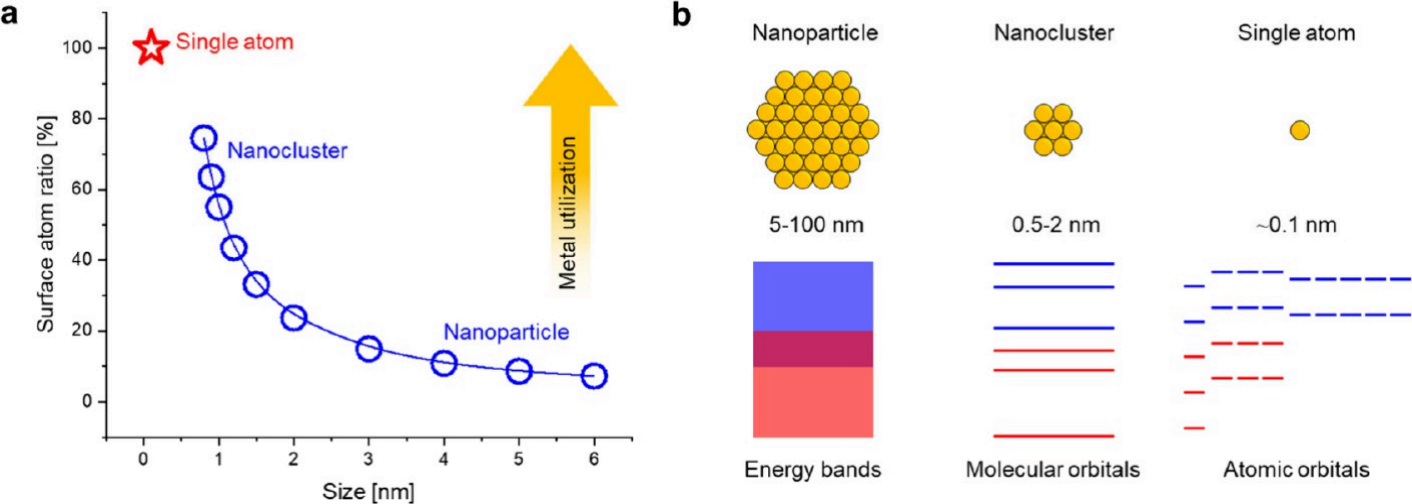
(a) Surface atom ratio and (b) electronic structure as a function of particle size.

Single-atom catalysts (SACs), consisting of isolated metal atoms anchored on suitable supports, achieve the full utilization of the active material because every atom is exposed and participates in the reaction.^19^ The most evident consequence of this approach is that it drastically reduced the amount of metal, addressing important economic challenges associated with the high costs of traditional noble metal catalysts.^20^ Moreover, the introduction of SACs has not only advanced our understanding of catalytic processes but also bridged the gap between heterogeneous and homogeneous catalysis by providing uniformly distributed, atomically dispersed active sites.^21^ Metal–support interactions in SACs play a decisive role in determining their selectivity, activity, and stability.^22^ Indeed, the unsaturated coordination environments of SACs not only stabilize isolated atoms but also tune electronic structures and adsorption and activation energies of reactants, facilitating a specific reaction pathway over the competing ones.^23^ As a result, SACs can be engineered to enhance the selectivity toward a desired reaction.^24^ Furthermore, the atomic-scale confinement of active centers in SACs offers opportunities to improve the turnover rates, i.e., the intrinsic catalytic efficiency of each metal site, designing electrocatalysts with unparalleled activity.^25,26^ However, SACs are not without challenges. The high surface free energy of SAs makes them prone to aggregation into NPs, losing any advantage associated with discrete atoms. Therefore, robust strategies for firmly anchoring SAs on substrates are required to ensure stability during reactions.^27^ Besides, the simple single-site structure of SACs may limit their performance in complex reactions, where synergistic effects due to the combination of different active sites and/or NPs/NCs could be necessary.^28^

With the exponentially growing interest in these novel catalysts, advanced characterization techniques with atomic resolution, such as high-angle annular dark-field scanning transmission electron microscopy (HAADF-STEM), X-ray absorption spectroscopy (XAS), and in situ/operando methods, provide fundamental knowledge in unraveling the geometry and unique properties of SACs.^29,30^ In combination with density functional theory (DFT) calculations, these techniques have enabled the discovery of extraordinary insights into the structure–activity relationships and mechanisms underlying electrocatalysis by SACs.^31^ Such advancements have paved the way for the rational design of efficient and robust SACs for a plethora of electrocatalytic processes, ranging from the well-known OER and HER,^32,33^ ORR,^34,35^ CO_2_RR,^36,37^ and electrochemical NH_3_ production,^38^ as discussed in authoritative literature survey articles whose reading is recommended by the authors of this work.

This review stands apart by providing a comprehensive examination of SACs as an innovative approach bridging atomic-scale engineering of materials and electrocatalysis. In particular, it highlights the most significant developments in the field over the past five years, offering an up-to-date perspective on recent advancements and emerging trends. It delves deeply into the methodologies for SAC preparation, with a detailed discussion of both top-down and bottom-up synthesis. It highlights the fundamental principle and application of advanced microscopy and spectroscopy techniques to characterize catalysts at the atomic level, and emerging in situ/operando methods, which shed light on the evolution of SACs during the electrocatalytic reaction. A distinctive feature of this review is also its emphasis on computational methods to model SA microenvironments, presented as vital tools to fully comprehend the potential of SACs. Additionally, a broad spectrum of electrocatalytic reactions by SACs has been presented, focusing on the most recent, original, and impactful studies. The described applications include not only OER, HER, ORR, CO_2_RR, and NH_3_ synthesis but also other less explored yet strategically relevant electrochemical processes, like those focusing on natural resource recovery, the conversion of waste sources into clean fuels and valuable chemical derivatives, and organic synthesis. By integrating insights from experimental advancements and theoretical studies, this review offers a comprehensive perspective on the current status, opportunities, and future directions for SAC research, aiming to provide a valuable resource for researchers in the field of electrocatalysis and a guide for the rational design of high-performance SACs tailored to diverse catalytic processes.

## 2 Synthesis of Single-Atom Engineered Catalysts

### 2.1 Support Materials

Support materials play a crucial role in SACs. Beyond serving as mere support platforms, they allow the stabilization of SAs, preventing their aggregation. Strong metal–support interactions influence SA electronic properties, ultimately defining their electrocatalytic performance.^39^ In addition, both SAs and the support can be directly involved in a synergistic catalytic process by binding intermediates and enhancing the reaction.^40^ Ideally, the support material should ensure good dispersion of SAs with high metal loading, excellent electrical conductivity, and good stability, so as to create the optimal environment for a desired electrocatalytic reaction. Various support materials have been used to design SACs, including carbon-based materials, metals, metal oxides, hydroxides, carbides, nitrides, sulfides, and transition metal dichalcogenides (TMDs), each with pros and cons.^41^

Carbon-based materials, such as graphene, carbon nanotubes, and N-doped carbon, are widely used as supports due to their low cost, high conductivity, large surface area, and chemical and thermal stability. Consequently, they enable efficient charge transport, which is highly desirable for electrocatalysis, and allow dispersing a high number of SAs, maintaining them stable under diverse conditions.^42^ For example, Ni SAs were dispersed on graphene for efficient and durable CO_2_RR,^43^ while Fe SAs anchored on carbon nanotubes proved to be an efficient catalyst for ORR to hydrogen peroxide (H_2_O_2_) in both alkaline and neutral media.^44^ Moreover, SACs using N-doped carbon supports demonstrated excellent performance over a wide range of electrochemical reactions, comprising OER, HER, ORR, and CO_2_RR.^45^ Besides, heteroatom doping in these carbon frameworks (e.g., N, S, or P) introduces active sites that strongly bind SAs and significantly affect their electronic properties, improving catalytic activity.^46^

Metal supports, which form single-atom alloys (SAAs), offer another approach to stabilizing SAs by exploiting strong metal–metal interactions. SAAs are characterized by higher activity and selectivity compared to monometallic catalysts, resistance to deactivation via coke formation, and resistance to CO poisoning.^47^ For instance, a RuAu SAA consisting of Au atoms dispersed on Ru NPs was prepared by laser ablation, revealing high activity for HER.^48^ Instead, a BiCu SAA with Bi atoms decorating Cu NPs showed higher selectivity for CO_2_RR into C_2+_ products rather than C_1_ ones compared to Cu NPs or BiCu nanocomposites.^49^

Metal oxides, such as TiO_2_, CeO_2_, and Fe_2_O_3_, are an alternative class of supports frequently employed for SACs. These materials are rich in surface defect sites, such as oxygen vacancies, or −OH groups which serve as binding sites for SAs. Additionally, their redox properties and surface acidity or basicity, reflecting their affinity toward electrons or protons, provide versatile catalytic environments for electrocatalytic reactions.^50^ Also, metal hydroxides, particularly, layered double hydroxides [M^2+^_1–*x*_M^3+^_*x*_(OH)_2_]^*x*+^[A^*n*–^]_*x*/*n*_, a class of two-dimensional (2D) materials formed by positively charged layers of divalent and trivalent metal ions (M^2+^ and M^3+^) and interlayer anions (A^*n*–^), are advantageous supports for the formulation of SACs owing to their large surface area and high tunability in terms of possible choices for M^2+^, M^3+^, and A^*n*–^, and M^2+^/M^3+^ ratio.^51^ Similarly, metal carbides,^52,53^ nitrides,^54,55^ sulfides,^56^ and TMDs^57^ are also effective supports for SACs combining large surface areas with excellent conductivity and stability. In this regard, the introduction of Pt SAs into defective 2D Mo_2_TiC_2_T_*x*_ MXene nanosheets enabled the improvement of the electrocatalytic activity toward HER.^58^ Similarly, the anchoring of Fe SAs on MoS_2_ nanosheets boosted the N_2_ reduction reaction to produce NH_3_.^59^

Metal–organic frameworks (MOFs) represent advantageous support materials due to their ordered porous structures and abundant anchoring sites for SAs, as well as potential cocatalysis.^60^ However, only a few examples of SAs immobilized in pristine MOFs have been reported. In contrast, MOFs are frequently converted into N-doped carbon supports by pyrolysis.^61^

In summary, while the choice of support materials is crucial for enhancing SACs electrocatalytic performance, it is vital to establish appropriate synthetic strategies that stabilize SAs in the support materials, avoiding their tendency to aggregate due to their high surface energy. Several synthetic strategies to solve this challenge have been developed. Here, we classified these strategies into two categories depending on the metal precursors: bottom-up and top-down strategies. Bottom-up strategy is a common way to prepare SACs, which uses the atomic metal precursors. In contrast, top-down approaches utilize bulk metal or metal NPs as precursors for SAC fabrication. The top-down strategies have been less developed than bottom-up ones because the bulk metal or metal NPs should initially undergo the dissociation of metal–metal bonds, which requires high energy. Nevertheless, it provides insights to solve the challenges associated with the aggregation of the metal atoms, allowing the fabrication of highly dispersed and stabilized SACs.

### 2.2 Bottom-Up Synthesis

#### 2.2.1 Atomic Layer Deposition

Atomic layer deposition (ALD) is a technique capable of depositing various thin film materials layer-by-layer from the vapor phase with atomic-level precision.^66,67^ The self-limiting characteristic of ALD process enables the homogeneous deposition of metal atoms on supports. Furthermore, this vapor phase deposition technique enables the penetration of metal atoms deep inside the porous substrate, which can maximize the loading of SA active sites.^68^ The ALD process includes four steps in one ALD cycle; (1) exposure to the first precursor vapor, (2) removal of excess precursor molecules and byproducts, (3) exposure to a second precursor vapor, and (4) removal of excess precursor molecules and byproducts. By controlling the number of ALD cycles, the metals can be built up from SAs to NPs. In the ALD process, the anchor sites are required to react with metal precursors. For example, Sun et al. reported that the reaction of O-functional groups of graphene nanosheets and (methylcyclopentadienyl)­trimethylplatinum (MeCpPtMe_3_) followed by the O_2_ exposure via ALD process could form Pt SA active sites on the graphene substrate.^62^ Stambula et al. further revealed that the N-functional groups also provide anchoring and nucleation sites for the SA Pt deposition by utilizing N-doped graphene as a support material.^69^ Metal oxides or metallic NPs can also react with metal precursor vapors.^70–72^ For example, Wang et al. reported a Pt_1_/CeO_2_ SAC, where the isolated Pt atoms are selectively located on the Ce rows of (110) and (100) facets of CeO_2_.^70^ Instead, Cao et al. selectively deposited SA Fe_1_(OH)_*x*_ only on the Pt NPs of the Pt/SiO_2_ by exposing the Fe precursor, ferrocene (FeCp_2_), and O_2_ at 393 K, which is a far lower temperature than the conventional ALD process using the same precursors (Figure 2a).^71^

**Figure 2:**
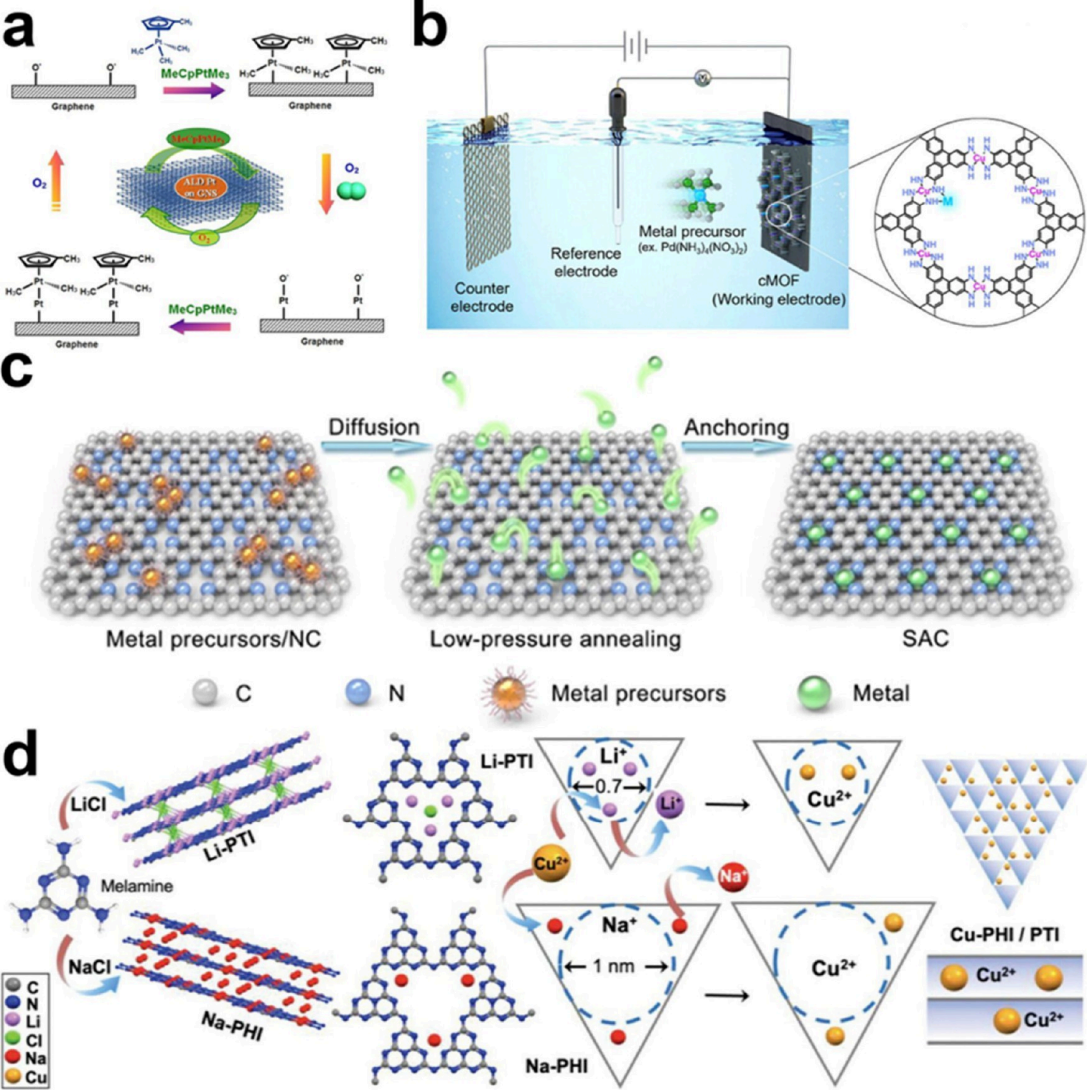
**Bottom-up synthesis of SACs.** (a) Schematic illustrations of Pt ALD mechanism on graphene nanosheets. Reproduced from ref 62. CC BY-NC-ND 3.0. (b) Schematic illustration of the synthesis of M1-cMOF by electrochemical deposition. Reproduced from ref 63. Copyright 2024 American Chemical Society. (c) Schematic illustration of the pressure-controlled metal diffusion approach for preparing SACs. Reproduced from ref 64. CC BY 4.0. (d) Synthetic scheme of Na-PHI, Li-PTI via molten alkali salt method and Cu-PHI/PTI samples through metal-ion exchange. Reproduced with permission from ref 65. Copyright 2024 Wiley-VCH.

Although the ALD technique provides a precisely controllable way to produce SACs, the high cost limits its large-scale and commercial usage. Furthermore, it requires specific metal precursors and the loading of a high amount of SA metals is still challenging because of the aggregation.^73^

#### 2.2.2 Electrodeposition

The electrochemical deposition technique enables the fast, facile, and controlled synthesis of SACs under mild operation conditions without the use of additional capping or reducing agents.^74–77^ During the electrodeposition process, electrons are directly delivered to metal ions at the electrode surface, where the thermodynamics and kinetics of the metal deposition can be easily controlled by adjusting potential, current density, electrolyte, and deposition time.^77^ Typically, low concentrations of metal precursors are utilized in the electrodeposition of SAs to prevent the growth of the metal.^78–82^ For instance, Zhang et al. prepared an atomically dispersed Pt catalyst on the NiFe-layered double-hydroxide (LDH) surface via electrodeposition.^83^ The catalyst possessed an axial chlorine ligand, which was reversibly exchangeable with a hydroxide ligand retaining the single Pt sites on the support. Lei et al. reported a synthetic strategy to load SA Ir on Ni_3–*x*_Fe_*x*_S_*x*_ with high surface density (Ir_1_/NFS) via a two-step electrodeposition.^84^ They separated the substrate construction and the deposition of Ir, which induced the achievement of Ir SAs on the surface of the substrate to maximize Ir utilization. Recently, Park et al. demonstrated that SACs can be made from MOFs via the electrodeposition method.^63^ Specifically, they reported the introduction of Pd SAs to the conductive MOF Cu_3_(HITP)_2_ (HITP = 2,3,6,7,10,11-hexaimino triphenylene) via cathodic deposition with the sweeping potential range from 0.513 to 0.013 V vs Ag/AgCl (Figure 2b). Galvanic replacement is another widely adopted strategy for the preparation of SACs on metal supports, also known as SAAs.^85–88^ The difference in the standard reduction potential between two metal species leads to the spontaneous redox reaction. The metal ion precursor with a higher reduction potential is reduced by oxidizing the metal atom on the surface of metallic supports, which has a lower reduction potential. The oxidized metal atom dissolves into the solution and the reduced metal precursor occupies the site, producing a SAA. For example, Wang et al. prepared Ir_1_Ni SAAs supported on MoO_2_ for alkaline water electrolysis by this method.^88^

#### 2.2.3 Pyrolysis

The pyrolysis method is the most common way to produce carbon-based SACs from metal–organic composites.^89–91^ MOFs and polymers with metal anchoring ligands are mainly used as precursors. These organic precursors are condensed to bulk carbon networks remaining metal coordination sites. By using organic precursors or inorganic additives containing various metal-anchoring ligands, studies on the relationships between the active site structure and catalytic activity have been conducted.^92–96^ Although the organic molecules work as barriers to resist metal sintering or agglomeration during the process to some extent, the preparation of SACs with high metal loadings is challenging due to the undesired formation of bulk metal particles at elevated temperatures, which requires additional acid treatment.^97,98^ Recently, several strategies were developed to prepare SACs with ultrahigh metal loadings, such as the utilization of additives with metal anchoring sites,^99,100^ big organic ligands,^101^ and slow-release synthesis (SRS).^102^ For example, Xia et al. utilized surface-functionalized graphene quantum dots (GQDs) as precursors,^101^ which possess numerous metal-coordinating sites and do not undergo significant structural change during the pyrolysis process, to prepare Ir–, Pt–, and Ni–N–C with the metal contents of 41.6, 32.3, and 15 wt %, respectively. Gu et al. reported an SRS strategy to prepare a Cu SAC with high metal utilization (97%) and a controlled coordination structure.^102^ They utilized natural montmorillonite (MMT) as a Cu^2+^ precursor, mixed it with melamine, and pyrolyzed the mixture. Melamine formed C_3_N_4_ emitting NH_3_, and this NH_3_ slowly liberated the Cu^2+^ from the Cu^2+^-exchanged MMT. The released Cu^2+^ coordinated with C_3_N_4_ where the coordination structure was controlled to form Cu–N_4_.

While the above-mentioned methods are for the simultaneous production of SA sites and carbonaceous supports, postsynthetic strategies to prepare SACs via annealing of the mixture of metal precursors and support materials are also available. For example, Hai et al. could prepare ultrahigh-density SACs with metal loadings up to 23 wt % via a two-step annealing method.^103^ First, the metal precursors were introduced via wet-impregnation to the support materials. After drying the solvents, the mixture was annealed below the temperature where the metal precursors are decomposed, to maximize the metal coverage and to enable the removal of excess species by washing. This low-temperature annealing process prevented the metal sintering during the second high-temperature annealing step to remove residual ligands and stabilize metal atoms. Besides, the annealing process under reduced pressure was found to be another effective way to prepare SACs with high-metal loadings.^64,104^ Al-Hilfi et al. found that the reduced pressure could increase the SA metal loading by 3.2 times compared with ambient pressure during the pyrolysis process to synthesize SACs from various metal precursors (Fe, Co, Ni, Mn, Cu, Mo, Pd, and Ru) and porous N-doped carbon (Figure 2c).^64^ Computational studies revealed that the diffusion of metal atoms was significantly enhanced at the reduced pressure due to the long mean free path of the atoms, which minimized agglomerations. A similar process was reported by Wang et al. nearly at the same time, which supports the validity of the proposed strategy.^104^

The pyrolysis method possesses the advantages of simple operation and the use of inexpensive precursors. Furthermore, it is suitable for fabricating SACs with abundant SA loadings and with various ligand environments. However, the formation of NPs or NCs at high temperatures is still a big challenge.

#### 2.2.4 Wet-Chemical Synthesis

The wet-chemical methods have been widely utilized to fabricate SACs on the prepared support materials due to their easy operation and feasibility of large-scale production.^105,106^ During the wet-chemical process, the metal ions are adsorbed on the surface of support materials via coordination bonds with the ligands or electrostatic interactions, followed by drying, reduction, or stabilization.^107,108^ For instance, Roy et al. prepared Cu–N_2_ SACs supported on crystalline 2D carbon nitrides (2D-CN) via wet chemical method (Figure 2d).^65^ They prepared two 2D-CNs of poly­(heptazine imide) and poly­(triazine imide) which contain Na^+^ and Li^+^ cations, respectively. Those cations were replaced by Cu^2+^ through the cation exchange in aqueous media, where the Cu^2+^ ions coordinated to N atoms of the 2D-CNs. Giulimondi et al. developed a standardized and scalable strategy by applying incipient wetness impregnation on carbon extrudates.^109^ Coprecipitation is another simple and fast wet-chemical method to prepare SACs by getting all the precursors of metal and support materials together during the synthesis. This strategy is utilized to fabricate SACs supported on metal-oxides,^110–113^ MOFs,^90,114–116^ metals,^117^ and high-entropy SA materials.^118^ For example, Zhang et al. prepared a Pt_1_Pd alloy for Li–O_2_ batteries by the reduction reaction of Pt­(acetylacetonate)_2_ and Pd­(acetylacetonate)_2_ in the oleylamine solvent using ascorbic acid and (acetylacetonato)­dicarbonylrhodium­(I) as the reducing agent and structure-directing agent, respectively.^117^

The wet-chemical method is an economical and simple way to fabricate SACs. However, it is difficult to synthesize SACs with high metal loadings due to the limited number of functional groups or defects on the support materials to anchor the metal precursors.

#### 2.2.5 Bottom-Up Ball-Milling

Ball-milling is a mechanochemical method where mechanical energy is applied to induce chemical reactions between the metal precursors and solid supports.^119^ Synthesis of SACs via the ball-milling process can be achieved in both bottom-up and top-down ways. The top-down ball-milling will be discussed in section 2.3.3, while in this section the bottom-up ball-milling is described. This process enables the anchoring of molecular metal precursors on the support material via the manipulation of chemical bonds with little or no solvent. For example, metal phthalocyanine (Pc) molecules such as FePc, MnPc, CoPc, NiPc, and CuPc could be embedded in graphene nanosheets by this method.^120,121^ Furthermore, this approach offers an easy scaling-up, enabling kilogram-scaled synthesis of highly dispersed Pt,^122,123^ Au,^124^ and Pd SACs,^125^ which is challenging in wet-chemical methods. Recently, modifications of the local environment of SACs via the ball-milling process were reported. Zhong et al. prepared a cathode for all-solid-state Li–S batteries with long life and high capacity through the ball-milling process.^126^ First, CoPc was embedded into the acetylene black matrix (Co@AB) as a S host. Then, the ball-milling process was used to mix S and Co@AB, which made the stable uniform dispersion of S preventing cracks and agglomeration during the charge/discharge process. In the case of Guan et al., they found that the ball-milling process can relocate the surface Cu SAs in the subsurface of Fe_2_O_3_, which showed unprecedented dynamic and adsorption behaviors during the catalysis.^127^ The reconstruction of surface Fe_2_O_3_ during the ball-milling process positioned the Cu SAs in a depth of around 1–5 nm, which not only significantly contributed to the catalytic activity, but also prevented catalyst poisoning and sintering at the elevated temperature. Ball-milling method also provides a way to prepare SAAs. For example, Sun et al. could prepare M/Ni SAAs (M = Re, Ir, Ni) using the Ni balls and metal precursor solution containing perrhenic acid, chloroiridic acid, or nickel acetate, followed by drying, calcination, and reduction.^128^

The ball-milling method enables the simple fabrication of highly and uniformly dispersed SACs on a large scale. Furthermore, it uses the least amount or no solvent, which is friendly to the environment. However, the process takes longer time compared to other methods.

#### 2.2.6 Others

Many other methodologies to fabricate SACs have been developed, such as physical vapor deposition (PVD),^129^ chemical vapor deposition (CVD),^130^ microwave synthesis,^131^ and the laser planting method.^132,133^ The PVD and CVD methods are similar to ALD in that the metal precursors are vaporized and uniformly deposited on the substrate. In the PVD process, the metal precursors are only adsorbed on the surface or inside the pores of substrates, while in the CVD process, the metal precursors undergo chemical reactions to form SA sites. However, additional thermal treatment at high temperatures is required to stabilize the SAs in both PVD and CVD processes. The microwave synthesis method offers an easy and time/energy-saving way to prepare carbon-based SACs within a few minutes. Microwave rapidly heats up and carbonize the organic precursors within a short time, inhibiting the migration and agglomeration of metal atoms during the synthesis of SACs. However, this method remains uncultivated because most organic precursors have poor microwave absorption. The laser planting method enables the fabrication of SACs supported on various substrates, such as carbons, metals, and oxides, at an atmospheric temperature and pressure. The laser pulses create the defects on substrates and simultaneously produce SACs from the metal precursors. The development of such novel techniques is expected to offer fast and convenient ways to fabricate precise and controlled SACs in the future.

### 2.3 Top-Down Approaches

#### 2.3.1 Thermal Atomization

Nanoparticle atomization strategy consists of atomizing the metal NP by evaporation or diffusion. In 2016, Jones et al. reported an NP-to-SA Pt strategy by thermally vaporizing Pt under oxidizing conditions.^137^ Based on the fact that volatile PtO_2_ is formed from the bulk Pt at 800 °C in oxidizing conditions, they tried to trap the Pt vapor at downstream. They exploited the characteristics of CeO_2_ in that they not only improve the sinter resistance of metals by lowering the surface energy of NPs but also trap Pt atoms in an isolated state at elevated temperatures. Wei et al. demonstrated that the N-doped carbon derived from ZIF-8 MOF can be also utilized to downsize Pd, Pt, and Au NPs into SAs.^138^ Other than noble metals, Ni NPs were found to be atomized via thermal diffusion through the defect-containing N-doped carbon support (Figure 3a).^134^

**Figure 3:**
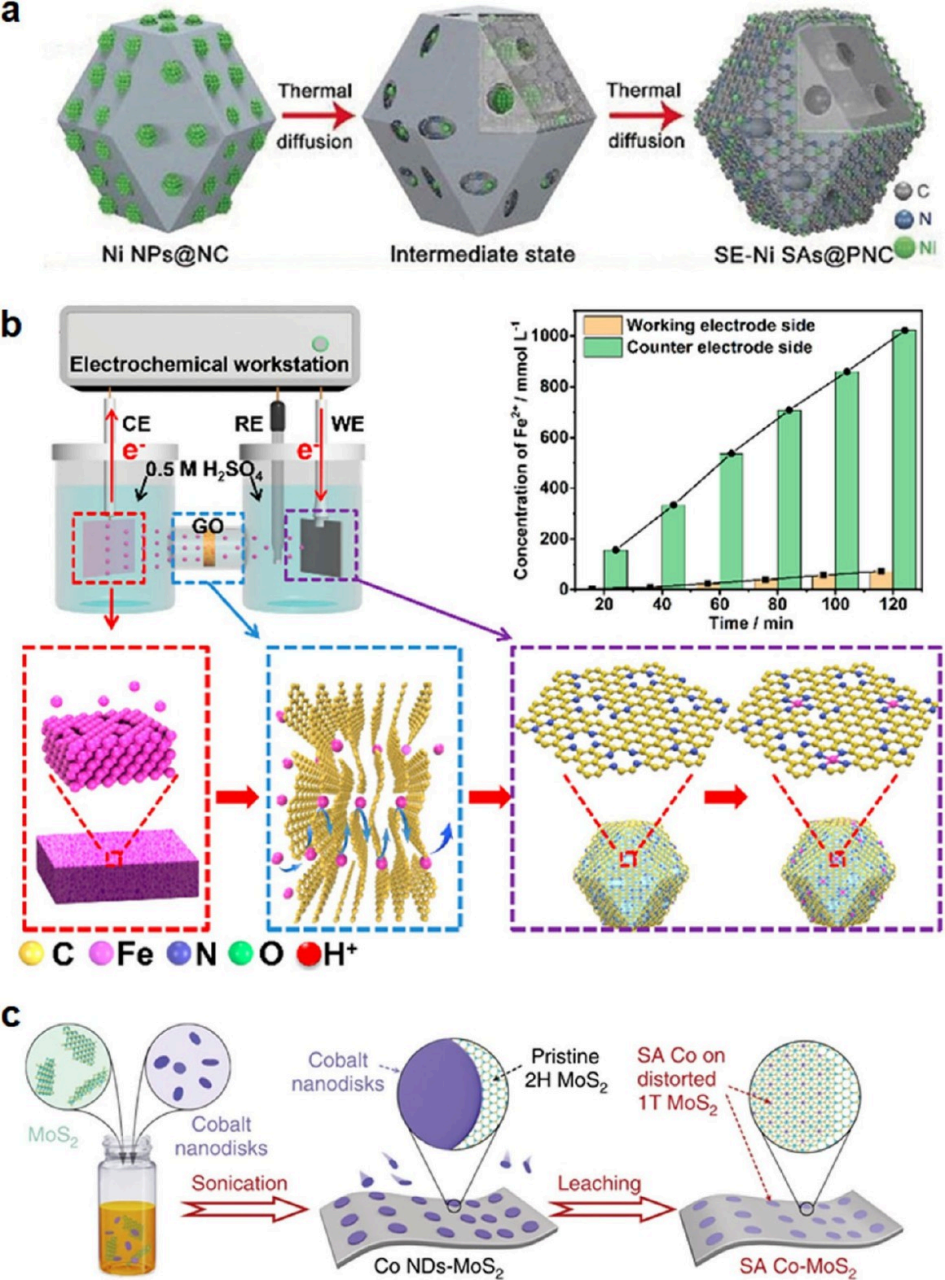
**Top-down synthesis of SACs.** (a) Schematic illustration of the transformation of Ni NPs to SAs via thermal diffusion. Reproduced with permission from ref 134. Copyright 2018 Wiley-VCH. (b) Schematic illustration of the formation of Fe–N–C SAC via anodic stripping method. Reproduced from ref 135. Copyright 2020 American Chemical Society. (c) Schematic illustration of the fabrication process of SA Co on MoS_2_ nanosheets via the assembly/leaching strategy. Reproduced from ref 136. CC BY 4.0.

Despite these findings, this method suffers from several challenges, such as high operation temperature to break metal–metal bonds, lack of universal approach, and low degree of atomization due to the Ostwald ripening limitations.

#### 2.3.2 Anodic Stripping

Top-down synthesis of SACs is also available via electrochemical methods, such as anodic stripping. This strategy utilizes the counter electrode as a sacrificial metal donor.^58,135,139–142^ Through the anodic dissolution of the counter electrode, the target metal ions are released to the electrolyte and subsequently adsorbed and reduced on the working electrode to produce SACs. The dissolution amount can be controlled by changing the anode voltage,^143^ pH,^144^ and purging gases.^145^ Again, achieving a low amount of dissolved metal is crucial to avoid the undesired formation of NPs. Wang et al. reported an electrochemical filtration strategy utilizing a graphene oxide membrane (GOM) to prepare SA Fe supported on porous N-doped carbon (Fe–N–C) (Figure 3b).^135^ The Fe^2+^ ions were generated from a bulk Fe foil and transported by the electric field through the GOM, reaching the working electrode. The GOM reduced the diffusion rate of Fe^2+^ during the process, thereby preventing the aggregation and growth of nuclei. Qi et al. developed an assembly/leaching strategy to fabricate a Co SAC selectively on the basal plane of MoS_2_ (Figure 3c).^136^ They first assembled the Co nanodisks (NDs) with MoS_2_ nanosheets via an ultrasonic process. The Co–S bonds induced by the sonication allowed close contact between the Co NDs and MoS_2_ surface. The Co NDs were leached via electrochemical cyclic voltammetry treatment, forming an SA Co array on the MoS_2_ surface. Thus, this strategy enables the synthesis of SACs on the electrode at room temperature by controlled metal leaching.

#### 2.3.3 Top-Down Ball-Milling

Recently, the ball-milling process has been utilized to prepare SACs by using bulk metallic precursors. In 2021, Wang et al. reported a unique top-down ball-milling strategy to prepare Co SACs from bulk Co and S,N codoped carbon (SNC).^146^ They claimed that the higher bonding energy of Co–N compared to that of Co–Co in bulk Co enabled the dissociation of surface Co atoms followed by anchoring the detached Co atoms on SNC. Han et al. further developed this top-down ball-milling strategy by using metal balls as precursors for SACs.^147^ In particular, Fe balls and graphite were mixed and ball-milled at 450 rpm for 30 h under an N_2_ atmosphere. During the process, the Fe balls were abraded into SAs by the graphite, and the N_2_ gas underwent catalytic dissociation on the activated surfaces of the Fe balls, thereby forming metal anchoring sites on the graphite framework. They further revealed that the heat treatment during the process was advantageous to improve thermal stability and increase the density of SAs by further transforming Fe_3_C clusters into SAs. Theoretical investigations demonstrated that the adatoms on the Fe balls generated from the repeated collisions of Fe balls with graphite are easily detached by the strong Fe–N bond with the N-doped graphite NPs via a hopping mechanism to form SA Fe sites. The versatility of this strategy was proved by using other metal balls, such as Co, Ni, Cu, and brass, and other support materials, such as MgO, SiO_2_, CeO_2_, and C_3_N_4_. Hence, top-down ball milling strategy may pave a novel way for the large-scale synthesis of SACs.

## 3 Characterization of Single-Atom Catalysts

The precise identification of the structure, electronic properties, and catalytic behavior of SACs is critical for understanding their unique performance in electrochemical catalysis. This section outlines key characterization techniques used to unravel these features, as summarized in the schematic illustrations of Figure 4.

**Figure 4:**
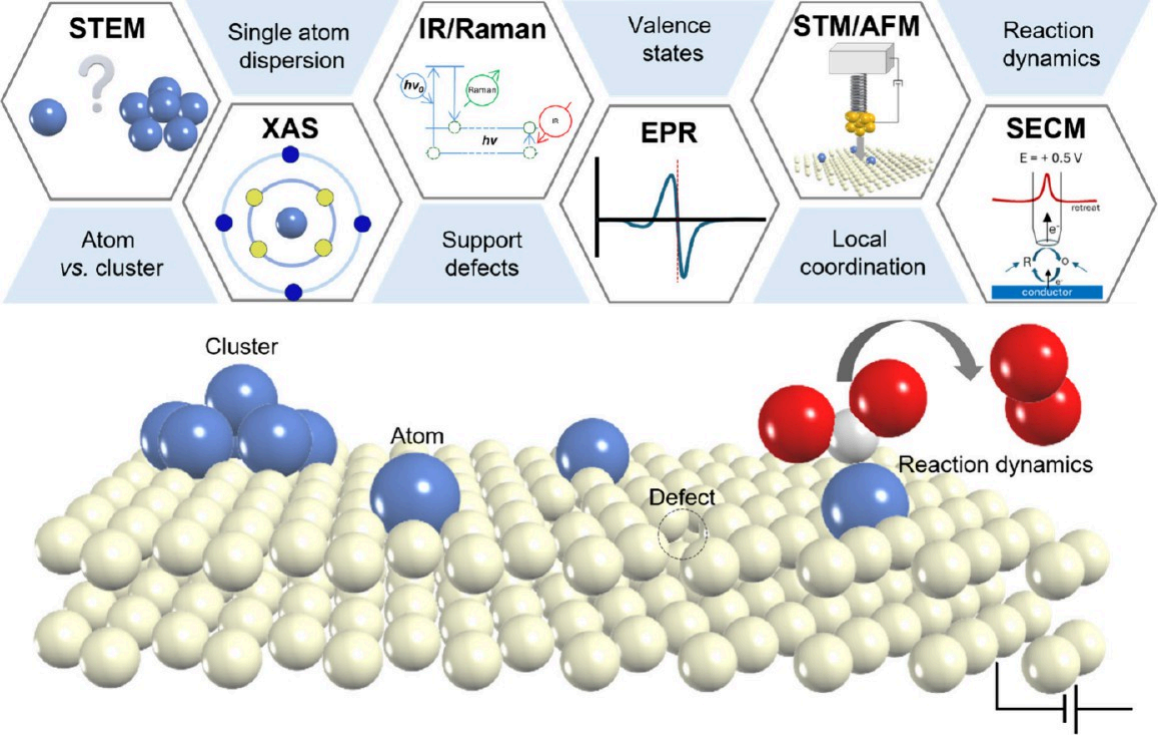
**Schematic summary of characterization techniques for SACs.** Selected characterization techniques provide morphological and structural information about SACs as well as their potential to probe reaction dynamics under in situ/operando conditions.

### 3.1 Electron Microscopy

#### 3.1.1 High-Angle Annular Dark-Field Scanning Transmission Electron Microscopy

The physical properties of materials are governed by their chemical composition and structural arrangement. Therefore, accurately identifying and reliably quantifying the type of atoms, their positions within the matrix, as well as their quantity, uniformity, and dispersion, is critically important.^148^ Direct visualization provides invaluable insights into a system and serves as a critical parameter in guiding successive synthesis processes. Achieving imaging at the SA level on substrates presents significant challenges. Ultrahigh vacuum electron microscopy techniques are essential for resolving individual atomic features. Among these methods, scanning transmission electron microscopy (STEM) stands out as a highly effective tool for SA characterization. In STEM, detectors collect electrons emitted from the sample surface based on their scattering angles. Annular dark-field (ADF) microscopy is an imaging technique where the scattered electrons beyond the convergence angle of the incident electron beam are collected by the detectors.^149^ A high-angle annular dark-field (HAADF) detector specifically collects the signals from scattering angles of 50° or greater, producing a HAADF image. These images provide atomic-level resolution and are highly sensitive to the number of atoms within the atomic columns. Importantly, HAADF STEM image intensities exhibit *Z*-contrast, where the heavier metal elements appear brighter. Figure 5a–c shows that two distinct crystalline regions with well-defined atomic arrangements reported by Zhu et al., designated as Pos 1 and Pos 2, have been identified as corresponding to the RuO_2_(110) and Ru(101) facets, respectively.^150^ Notably, due to the higher atomic number (*Z*-contrast) of Pt compared to other elements, isolated bright spots representing Pt atoms are directly observed. These Pt atoms are uniformly distributed across both the Ru and RuO_2_ regions, indicating that Pt predominantly exists as SAs on the Ru/RuO_2_ support structure.

**Figure 5:**
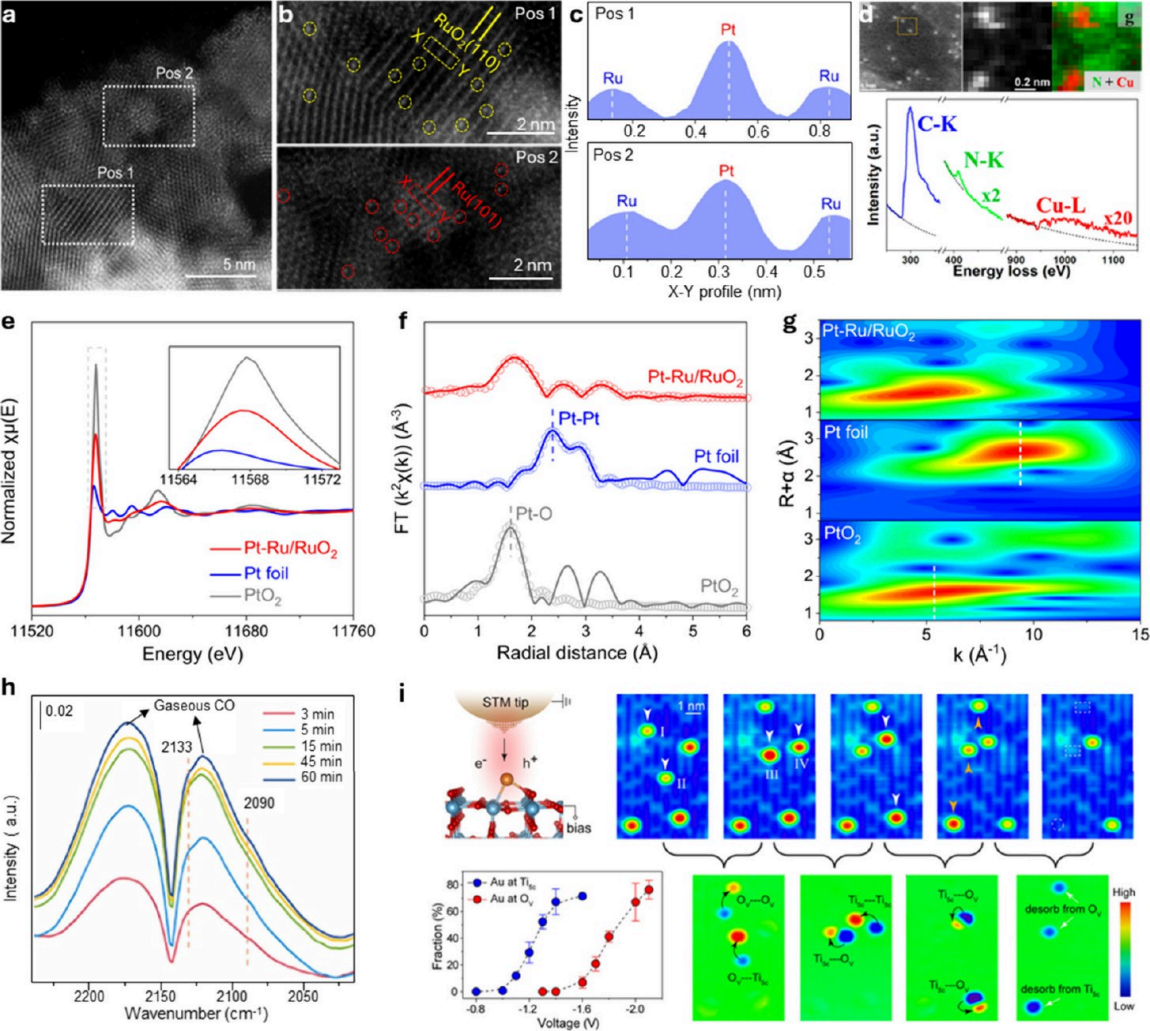
**Selected structural characterization techniques for SACs.** (a) AC-HAADF-STEM image of Pt–Ru/RuO_2_. (b) Enlarged area of Pos 1 and Pos 2 from (a), with the Pt SAs marked in circles. (c) Atomic intensity profiles along the dashed rectangles in (a). Reproduced from ref 150. CC BY 4.0. (d) EELS characterization of a Cu–N–C SAC. Atomic resolution HAADF-STEM image with a yellow rectangle representing the area of EELS mapping. Simultaneously acquired HAADF image and EELS composite map of N and Cu. The EELS spectrum extracted from a single Cu site in the HAADF image shows the presence of N and Cu signals. Reproduced from ref 153. Copyright 2021 American Chemical Society. (e) Normalized Pt L_3_-edge XANES spectra of Pt–Ru/RuO_2_, Pt foil, and PtO_2_. (f) Pt L_3_-edge EXAFS spectra and corresponding fitting curves. (g) Wavelet-transformed *k* ^2^-weighted EXAFS spectra of Pt–Ru/RuO_2_, Pt foil, and PtO_2_. Reproduced from ref 150. CC BY 4.0. (h) In situ DRIFTS spectra of V_Cu_–Au_1_–Cu SACs under CO flow at different times. Reproduced with permission from ref 161. Copyright 2022 Elsevier. (i) Schematic STM drawing of the electron or hole injection from a tip with positive or negative pulses. The following top row graphs show consecutively acquired images after applying negative or positive voltage pulse over the marked Au SAC. The white arrows indicate the negative voltage pulse (−2.0 V) and the orange arrows indicate the positive voltage pulse (+2.8 V) over the Au SAC. The dashed circles and rectangles indicate the sites after the desorption of Au SAC. The first graph in the second row shows fractions of the moved Au SAs from the Ti_5c_ sites and O_V_ sites, plotted as a function of applied negative voltages. The image differences were subtracted and shown in the second row. Reproduced from ref 173. Copyright 2019 American Chemical Society.

#### 3.1.2 Electron Energy Loss Spectroscopy

Electron energy loss spectroscopy (EELS) is a robust method for analyzing individual atoms within materials. This technique measures the energy lost by electrons traversing a sample, providing insights into the atom’s electronic structure and chemical composition. HAADF-STEM can reveal the distribution of particle sizes, and by incorporating an electron spectrometer, it provides the measurement of EELS.^151^ When electrons interact with atoms, they can excite inner-shell electrons to higher energy states, resulting in energy loss. The specific energy of this core-loss electron is unique to each element, enabling precise identification. Additionally, EELS can investigate an atom’s chemical state by examining the core-loss edge’s fine structure. This approach has become increasingly vital in studying SACs, where understanding the electronic configuration and chemical environment of individual atoms is essential for enhancing their catalytic efficiency. EELS is widely employed to investigate the chemical composition and bonding states of materials. When used in conjunction with transmission electron microscopy (TEM), it can identify atomic components of individual molecules. However, this application is not commonplace due to the high accelerating voltages (100–2400 kV) typically used in TEM to achieve high spatial resolution, which can cause electron damage to the molecules under examination. Both EELS and energy-dispersive X-ray spectroscopy (EDS) offer effective methods for elemental analysis in TEM, particularly for locating isolated atoms and assessing their distribution uniformity across catalysts, especially in samples with low atomic numbers. To perform SA spectroscopy, high-energy incident electrons are necessary, which inevitably results in significant structural damage to the specimens.^152^

HAADF imaging primarily relies on incoherent elastic scattering, where electrons change their propagation direction without losing energy. However, some electrons experience inelastic scattering as they interact with the sample through processes such as inner-shell ionizations, inter- and intraband transitions, and the excitations of plasmons, excitons, and phonons. These inelastic interactions cause the transmitted electrons to lose energy specific to the interaction, providing critical information about the material’s local coordination, chemical environment, and electronic and vibrational structures. The energy loss of inelastically scattered electrons can be measured using a magnetic-prism spectrometer. After passing through the projector lenses and HAADF detector, the electron beam is directed into the spectrometer via an EELS aperture. Within the magnetic prism, electrons are deflected along trajectories based on their kinetic energy. These electrons, with varying energy losses, are refocused onto an EELS detector to generate an electron energy-loss spectrum. In the core-loss regime (>50 eV), the spectrum exhibits characteristic peaks for different elements, similar to EDS, enabling elemental mapping. EELS offers superior energy resolution compared to EDS, allowing for a more detailed analysis of the fine structure in ionization edges. By examining these features, the local coordination environment and oxidation states of SAs can be determined. Additionally, EELS is highly effective for identifying light elements, analyzing composition, and probing chemical bonding environments. It also provides insights into the electronic and vibrational properties of materials, making it an invaluable tool for advanced material characterization.^29^ As shown in Figure 5d,^153^ the EELS analysis from a single atomic site with brighter contrast in HAADF imaging of a Cu–N_3_ SAC reported by Yang et al. shows clear C, N, and Cu signals suggesting that the Cu atoms are most likely coordinated with C/N elements, confirming the already suggested M–N_*x*_ coordination of the catalyst. However, the weak signal from atomic centers and their movement under the electron beam remain the main challenges for using EELS to determine the oxidation state of SAs.

#### 3.1.3 Energy-Dispersive X-ray Spectroscopy

Similar to EELS, EDS elemental mapping plays a critical role in determining the distribution of elements within a catalyst. With the advent of large-area detectors, EDS has enabled SA-level analysis. This technique is particularly useful for confirming the location and dispersion of atoms across a support material. EDS can be utilized in both TEM and STEM modes. Typically, when an electron beam passes through the sample, it can eject an electron from the inner shell of the atom, leaving a hole. An electron from the outer shell can fill this hole by releasing an X-ray with energy corresponding to the energy difference between the two shells. Since every element has characteristic energies, EDS enables spatially resolved elemental mapping.^29^ Combined with high-magnification HAADF images, EDS provides a robust method for assessing the dispersity of SAs across the support. While some studies have successfully mapped SAs spatially using EDS, they required careful quantitative evaluation of the counts to confirm that the signal originated from an SA rather than a small cluster. Despite its utility, EDS faces limitations, such as a 2-fold lower collection efficiency compared to EELS, making quantitative EDS challenging for SA analysis. As a result, most studies rely on lower-magnification EDS for dispersity evaluation, while HAADF imaging remains the primary microscopy characterization technique for SACs.

Electron microscopy techniques face significant challenges in characterizing SACs compared to NPs and bulk materials. Conventional TEM struggles with the low contrast of isolated atoms against support, while HAADF-STEM, though capable of atomic-resolution imaging, requires high electron dosages that can cause beam-induced damage or atom migration. Unlike NPs with defined lattice structures, SACs lack periodicity, making crystallographic analysis via selected area electron diffraction (SAED) or fast Fourier transform (FFT) ineffective. Additionally, EELS can provide chemical state information but suffers from low signal intensity and background noise. Sample preparation can also alter SAC dispersion, leading to aggregation or redistribution. Furthermore, statistical challenges arise in confirming SAC presence across large sample areas. Due to these limitations, electron microscopy alone is often insufficient for SAC characterization and is typically complemented by spectroscopic techniques such as XAS and XPS to obtain reliable structural and chemical insights.

### 3.2 X-ray Absorption Spectroscopy

X-ray absorption spectroscopy, including X-ray absorption near-edge spectroscopy (XANES) and extended X-ray absorption fine structure spectroscopy (EXAFS), provide detailed information about SAs coordination environment, binding geometry, and oxidation states. When combined with morphological data, these techniques allow for the confirmation of the presence of SAs.^24^ Typically utilizing soft and hard X-ray synchrotrons as the light sources, XAS can either be performed to characterize catalysts as it is, or during reaction conditions, investigate catalytically active sites in situ. These in situ/operando studies will be further discussed in the following sections.

In combination with standard material characterization techniques such as XRD and XPS, the first set of information acquired from XAS involves the oxidation states of metal species on support substrates. For instance, Zhu et al. analyzed XANES data and observed that the absorption edge position of Pt–Ru/RuO_2_ lies between Pt foil and PtO_2_. This finding indicates that the Pt species deposited on RuO_2_ exists in an intermediate oxidation state between 0 and 4+ (Figure 5e–g).^150^ Similarly, Zhou et al. reported that the Fe absorption edges of the P/Fe–N–C structure were located between FeO and Fe_2_O_3_, implying the valence of Fe is between 2+ and 3+.^154^ Cao et al. reported a design of SAAs with V/Mo/W atoms on Bi nanosheets. The V–Bi XANES absorption edge is close to that of V foil but shows a negative shift, likely due to electron transfer from Bi to V, indicating strong p–d orbital interactions within the SAA.^155^

Additionally, EXAFS offers valuable insights into the binding geometry and bond lengths, though analyzing the data involves a complex fitting process. The existence of SA metals is usually confirmed by the absence of M–M bonds. These SAs bonding structures can be categorized into three types. (1) Metal–oxygen formation. SAs usually form an M–O bond with the oxide-based support for dispersed active sites. For Pt_1_ supported on RuO_2_ surfaces, isolated Pt is coordinated by surrounded O atoms from RuO_2_ substrates, as identified by EXAFS analysis.^150^ Similar cases have been reported for Co SAs on RuO_2_ surfaces.^156^ (2) Metal + nonmetal coordination (excluding oxygen, with C, N, or S, for example).^157,158^ Yang et al. used Fourier transform (FT)-EXAFS analysis to confirm that the Cu–N–C SACs exhibited a Cu–N_4_ structure with a Cu–N bond length of 1.92 Å.^159^ Similarly, for the Ni–N structure, Luo et al.^160^ reported that one Ni atom was coordinated with four N atoms (Ni–N_4_). (3) M_1_–M_2_ structure. In the case of alloys, EXAFS data also confirms the formation of SAs localization, for example, Cu–Au–Cu contributions as reported by Zhang et al. to claim the Au–Cu alloy formation.^161^

Another key information obtained from EXAFS data analysis is the determination of the coordination number of SA metals. Yasin et al. derived from EXAFS data the precise coordination number of Fe and Co coordination with N, and S, respectively, enlightening a possible dual atom structure of diatomic coordination in Fe/Co dual atom catalysts.^158^ Zhang et al. proposed the local structure of In–N_3_S with the coordination of three N atoms and one S atom in their first shell structure.^157^

XAS is frequently employed to confirm the absence of metal clusters or NPs in SACs. However, this technique provides only area-average information across the entire catalysts, making it difficult to resolve valence states or coordination environments in nonuniform samples, particularly for minor species that may significantly influence catalytic activity. To gain a more complete understanding of the structure–property relationship in SACs, XAS is often combined with STEM, which complements it by offering structural information at different scales (global vs local) and perspectives (indirect vs direct). Moreover, XAS has limited sensitivity for detecting adsorbed reaction intermediates, especially in situ, due to the low scattering cross-section of light elements relative to metal atoms. Consequently, techniques like infrared spectroscopy are crucial for investigating the adsorption behavior of the active sites of SACs. XAS characterization of SACs also has several limitations compared to NPs and bulk materials. One major challenge is the need for high-quality reference spectra, as the coordination environment of SAs can be complex and difficult to model accurately. Unlike bulk materials and NPs, which exhibit long-range order, SACs have highly dispersed metal centers with varied local environments, making EXAFS fitting more challenging and sometimes ambiguous. Additionally, XAS provides an averaged signal from the entire sample, meaning it cannot distinguish between isolated SAs and small clusters if they coexist. The technique also requires synchrotron radiation, which limits accessibility.

### 3.3 Diffuse Reflectance Infrared Fourier Transform Spectroscopy

Diffuse reflectance infrared Fourier transform spectroscopy (DRIFTS-FTIR, or simply DRIFTS) is a powerful technique for examining molecular adsorbates on SAs in gas-phase reactions, though its application in characterizing SACs remains relatively uncommon. DRIFTS employs small molecular gaseous probes, such as CO or NO, to analyze the atomic structures and adsorption properties of active sites. CO is particularly effective because it binds strongly to many transition metals such as Pt, Pd, and Ni, but it binds weakly with Au, Ag, and Cu. Their vibrational modes are sensitive to the electronic structure and coordination of metal sites. While NO can also be used, its complex chemistry often complicates the interpretation of structural information for catalysts. The most common purpose of employing DRIFTS for SACs is to distinguish the formation of SAs from aggregates. Adsorption of CO on transition metals results in a stretching frequency of linear and bridge configuration, where the latter requires two adjacent metal atoms. Thus, the bridging adsorption peak is often used to indicate the presence of metal clusters and NPs. Li et al. used DRIFTS to characterize 0.4 wt % Pt anchored on CeO_2_/SiO_2_ via CO probe, where only a sharp single peak near 2100 cm^–1^ was shown and assigned to isolated cationic Pt sites.^106^ CO adsorption on Au_1_Cu surfaces was also reported, exhibiting a small feature around 2133 cm^–1^ assigned to Au^+^–CO species, associated with atomically dispersed Au on the supporting surface (Figure 5h).^161^

To date, most of the DRIFTS analyses in the field of SAC characterization are related to heterogeneous catalysis. One key challenge is the weak signal intensity from isolated SAs, as their low concentration results in lower adsorption site densities, making spectral interpretation more difficult. Additionally, DRIFTS spectra of SACs can be highly sensitive to the support material, leading to overlapping signals that obscure the vibrational features of metal–adsorbate interactions. Unlike NPs and bulk materials, which provide stronger, more defined spectral features due to higher metal loading and uniform coordination environments, SACs exhibit diverse local structures, complicating peak assignments. Moreover, DRIFTS analysis requires careful experimental conditions, as factors such as temperature, humidity, and gas-phase interactions can significantly influence the spectrum. Therefore, DRIFTS is often used alongside complementary techniques like XAS and XPS for more reliable SAC characterization.

To date, most of the DRIFTS analyses in the field of SA characterization are related to heterogeneous catalysis. The limited application of DRIFTS for SACs for electrocatalysis is because the carbon substrates and aqueous environments (for operando studies) used in electrocatalysts can exhibit strong absorption in the infrared wavelength region. Weak binding between the molecular probe (CO, NO, etc.) to noble SAs (especially the M–N–C type) also results in insufficient surface coverage for DRIFTS studies at room temperature. Additionally, certain molecular probe adsorption can result in undesirable surface reconstruction and metal SAs aggregation, highlighting the need for alternative approaches or optimized conditions for characterizing SACs for electrocatalysts using DRIFTS.

### 3.4 Electron Spin Resonance

Electron paramagnetic resonance (EPR), or electron spin resonance (ESR), is a powerful technique for characterizing SACs due to its sensitivity to unpaired electrons, which are often present in transition metal atoms or active sites in SACs. Additionally, EPR can identify ferro- and antiferromagnetic species through the ferromagnetic resonance phenomenon, where these species often serve as active sites or intermediates. For transition metals, EPR provides detailed information about the metal oxidation state, the local electronic environment, and the coordination geometry of the SACs. While EPR is a well-established method for characterizing isolated paramagnetic species, its use in studying operational SACs has been limited, primarily due to the technique’s complexity and the absence of standardized protocols. EPR spectroscopy serves as a powerful technique for assessing the dispersion of catalysts supported by metals if the metal species are EPR-active, such as paramagnetic, ferromagnetic, or antiferromagnetic materials.^162^ While EPR is a well-established method for characterizing isolated paramagnetic species, its use in studying real working SACs has been limited, primarily due to the technique’s complexity and the absence of standardized protocols.

Examples of using EPR to estimate Cu atomic distance in SACs stabilized on active carbon have been demonstrated by Faust Akl et al., where the quasi-in situ EPR spectrum of the synthesized catalysts confirmed the isolated Cu­(II) SA active centers without the presence of Cu aggregates.^163^ The analysis of EPR spectral line shapes has also been used to qualitatively assess the dispersion of Mn species on CeO_2_.^164^ The spectrum of the as-prepared catalyst displayed the characteristic EPR signal of Mn­(II), consisting of six hyperfine-split lines arising from the interaction between the electron spin (*S* = 5/2) and the ^55^Mn nuclear spin (*I* = 5/2). The narrow line width indicated minimal dipolar interactions between Mn­(II) centers, suggesting that a portion of the Mn­(II) species are well dispersed on the CeO_2_ surface. Besides providing information about the oxidation states and coordination environments from the SAC’s distinct spectral features, EPR can also provide information about the defects in supporting materials. Supporting materials, especially oxides, often possess oxygen vacancies as defect points for SA anchoring sites.^165^ Most techniques commonly used in heterogeneous catalysis lack sensitivity to defects or light elements in the support structures. This makes EPR uniquely valuable, as it can provide detailed insights into these features. When a sufficient concentration of defects is present, and the signals are strong enough, the long relaxation times of these systems enable pulse measurements to be conducted even at room temperature.^166^

EPR is limited to detecting paramagnetic species, making it ineffective for SACs in closed-shell states like Cu­(I) or Pt­(II). The weak and anisotropic signals of SACs, due to low concentration and diverse coordination environments, make spectral interpretation challenging. Additionally, interactions with the support can induce strong relaxation effects, further complicating detection. Unlike NPs and bulk materials, where unpaired electrons are more abundant, SACs exhibit localized spins, leading to broadened or unresolved signals.

### 3.5 Ultra-high-Vacuum-Based Model System

Although the above-mentioned characterization techniques for SAs are generally applicable to electrocatalysts, the electron microscopic imaging techniques are insensitive to elements with low atomic mass such as O. Moreover, STEM generates images representing the 2D projection of 3D atomic structures, where local coordination information in the *z* direction remains difficult to extract. Thin film-based model systems provide more precise insights into structure–activity relationships by enabling the determination of local bonding environments and their evolution under reaction conditions. Traditionally, researchers have relied on model catalysts based on well-defined single crystals under ultra-high vacuum (UHV) conditions to investigate the interplay between structure and catalytic properties. Recently, the study of model systems for electrocatalysis has advanced, with a growing focus on understanding how SAs can remain stable on model supports.^167^ Surface-sensitive techniques like scanning tunneling microscopy (STM) and noncontact qTip atomic force microscopy (qTip AFM) are powerful tools for studying SA model systems,^168,169^ offering high spatial resolution and detailed insight into atomic-scale interactions. The atomic-scale structures of support surfaces have been reported with subangstrom precision characterization.^169^ The technical advancement of scanning probe instruments enables simultaneous imaging with STM and noncontact AFM, providing detailed views of both atomic structure and electronic properties of the surface.

In STM, electrons in a metal-vacuum-metal junction can tunnel through the vacuum region, with their wave function decaying exponentially, allowing the measurement of tunneling current and providing high-resolution images of surface topography and electronic structure. A key limitation of STM is its inability to analyze insulating materials, as these are typically only conductive at room temperature due to impurities or defects, with conductivity decreasing exponentially at lower temperatures. This restricts the study of their electronic structure at the atomic and submolecular levels, which often require low temperatures for stable measurements. Atomic force microscopy (AFM), developed in 1985 by Binning, Quate, and Gerber, can be used to address this issue. Similarly to STM, AFM requires an atomically sharp tip close to a well-defined flat surface. However, the magnitude measured is the force between the tip and the sample. The exact behavior of tip–sample interaction depends on the tip shape and the chemical reactivity of its last atoms.

In practice, model systems of single crystals with well-defined low-index surface orientations are prepared under UHV conditions free from contamination. Metal elements in the form of SAs are typically deposited directly onto the surface via evaporation. These prepared SA model systems lack ligands, and no additional calcination or activation steps are carried out before the studies. Metal elements such as Cu, Ag, Au, Ni, Pd, Pt, Co, Ir, Fe, and Rh, have been reported to be deposited on oxide-based substrates such as CeO_2_, MgO, Fe_3_O_4_, and Fe_2_O_3_ in different surface orientations. Parkinson’s group recently reported the local coordination of Pt atoms binding on the α-Fe_2_O_3_(11̅02). By combining STM, XPS, and computational methods, they demonstrated that Pt could reconfigure the support lattice to enable pseudolinear coordination with surface O atoms.^170^ After depositing Pt, near-circular protrusions were observed by STM, consistent with isolated Pt_1_ atoms. Rh on Fe_3_O_4_(001) is another well-studied SA model system under UHV. Dohnálek’s team reported that Rh adatoms were found to bind to two undercoordinated O atoms on the surface, and in surface octahedral Rh atoms that have replaced one of the octahedral Fe atoms.^171^ They discovered that the catalyst becomes dynamically activated in the presence of surface intermediates but returns to its inactive state once the reaction concludes.^172^ Dong et al. applied STM to study the interfacial electronic coupling behavior of Au SAs on the model TiO_2_(110) system. By applying high negative voltage pulses, hole injections enable Au SAs at Ti_5c_ and O_V_ sites to move across the surface, with distinct threshold voltages reflecting their different electronic states. In contrast, positive voltage pulses (electron injection) cause the Au SAs to desorb rather than diffuse, highlighting the differing effects of holes and electrons on their behavior (Figure 5i).^173^

It has also been reported that Au adatoms preferentially occupy the oxygen vacancies on the TiO_2_(110) system, providing a potential candidate for photocatalytic reactions.^173^ These oxygen vacancies also provide low-energy preferential sites for metal adatoms to nucleate. A comparison of Au and Ag NP growth on the TiO_2_(110) surfaces suggests that the presence of hydroxyl groups accelerates sintering for both metals.^174,175^ It is important to note that due to the possibility of reducing model systems under UHV conditions, the sample surface oxygen vacancies are common defects as observed by bright protrusions in STM and as missing atoms in noncontact AFM. Although these oxygen vacancies are important since they are reactive both as chemical reaction sites and nucleation sites for metal NPs, in realistic electrocatalytic environments, these surface oxygen vacancies will react fast with ambient oxygen and water, thus limited surface oxygen vacancies are presented for application purposes.

While UHV techniques are capable of atomic-level surface analysis, they are extremely sensitive to contamination hence requiring tedious sample preparation and handling. SACs are highly sensitive to environmental changes, and UHV conditions can alter their oxidation state or coordination environment, leading to artifacts. Unlike bulk materials and NPs, which maintain stable structures, SACs may exhibit mobility or aggregation under UHV, complicating accurate analysis. Additionally, their low atomic concentration results in weak signals, making detection and quantification more difficult.

### 3.6 In Situ/Operando Characterizations

In-depth mechanistic studies require in situ/operando characterizations, as they provide invaluable insights into active sites, catalyst structure evolution, and reaction mechanisms. In situ and operando characterization techniques, when employed under reaction conditions, provide critical insights into the intricate kinetics of reactions, thereby facilitating a deeper understanding of the nature of active sites and the underlying reaction mechanisms.^176^ For a comprehensive understanding of the in situ characterizations, we refer the readers to these reviews for detailed information.^177,178^ This section provides a brief review of the recent development of operando techniques, including in situ XAS, EPR, infrared, and Raman methods as well as scanning electrochemical microscopy (SECM).

#### 3.6.1 In Situ XAS

XAS can be conducted under reaction/operando conditions, allowing in situ probing of active sites evolved during their interaction with adsorbed intermediates. Liu et al. applied operando XANES to study the CO_2_RR with Ni SACs with Ni–N_4_ coordination, and their results revealed that the Ni^+^ sites were highly active for CO_2_ activation above 0.4 V vs RHE during CO_2_RR, where Ni can act as a rapid electron donor to CO_2_ molecules (Figure 6a).^179^ Similar mechanisms have been proposed for atomically dispersed Ni on graphene substrates.^43^ The dynamic chemical and structural evolution of the active sites can also be investigated under operando conditions. In the Fe–N–C catalyst reported by Gi et al., the Fe^3+^ site maintains a 3+ oxidation state during electrocatalysis, leading to faster CO_2_ adsorption and weaker CO adsorption compared to traditional Fe^2+^ sites. When increasing the cathodic potential to −0.5 V vs RHE, the Fe K-edge of Fe^3+^–N–C shifted to lower energy, indicating Fe^3+^ reduction. As this reduction occurred, the coordination number of the first-shell ligands (Fe–N/Fe–C) dropped from 4 to 3, resulting in a significant decrease in CO_2_RR activity.^180^

**Figure 6:**
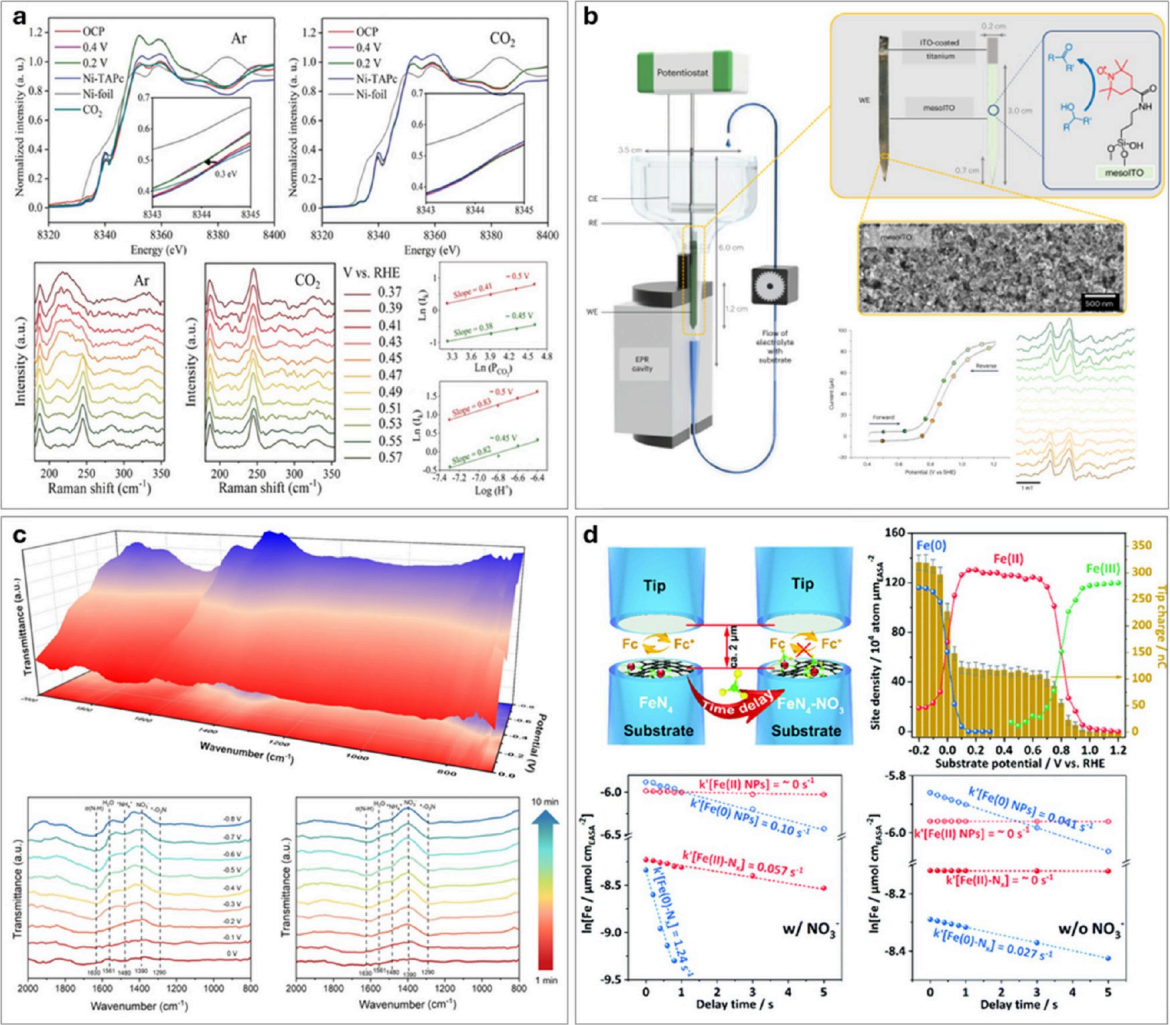
**In situ/operando characterization techniques for SACs.** (a) In situ XANES and Raman characterization of Ni–CNT–CC (nickel linked to carbon nanotubes via C–C coupling). Normalized Ni K-edge XANES spectra of Ni–CNT–CC acquired at various applied potentials vs RHE in 0.5 M KHCO_3_ aqueous solutions at room temperature under an atmosphere of Ar and CO_2_ (both at 1 atm). The inset shows enlarged Ni K-edge XANES. The OCP is about 0.57 V vs RHE. The CO_2_ line was obtained 5 min after switching Ar to CO_2_ at 0.2 V vs RHE. The second row shows Raman spectra of Ni-TAPc collected on an Au electrode at various potentials in 0.5 KHCO_3_ aqueous solution at room temperature in Ar and CO_2_ (1 atm). The figure on the right shows the reaction order of CO_2_ and H^+^, estimated from Koutecky–Levich analysis at different potentials. Reproduced with permission from ref 179. Copyright 2020 Wiley-VCH. (b) Cell design and assembled EPR setup with a three-electrode configuration (not drawn to scale). Ti wire connects the mesoporous indium tin oxide (mesoITO) working electrode to the potentiostat. Reference electrode: Ag/AgCl (3 M KCl); counter electrode: Ni wire. The *iR* drop was minimal at 27 Ω. The enlarged image on the top-right shows the mesoITO electrode with photographic and schematic representation. The bottom right graphs show the CV scan from the EPR cell placed inside the EPR cavity, recorded at 5 mV s^–1^. EPR spectra at the potentials highlighted on the CV scan during forward (green) and reverse (yellow) scans were shown at the bottom right. Reproduced from ref 183. CC BY 4.0. (c) Operando FTIR spectroscopy measurement of Fe–N/P–C SAC during NO_3_RR. Three-dimensional and planar FTIR spectra in the range of 800–2000 cm^–1^ under different given potentials and time-dependent planar spectra were obtained at an applied potential of −0.4 V vs RHE. Reproduced with permission from ref 185. Copyright 2023 Wiley-VCH. (d) In situ SECM for studying electrochemical NO_3_RR. The top-left scheme represents the SECM setup for the titration of Fe Sites. The top-right figure is the measurement of electrochemically active surface area (ECSA)-normalized active site density and corresponding titration charges of the Fe-Ppy SACs plotted against different potentials. The decay in the Fe­(II) and Fe(0) active site concentrations with different delay times is shown on the bottom with (bottom left) or without (bottom right) NO_3_^–^. Reproduced with permission from ref 189. Copyright 2021 Royal Society of Chemistry.

Soft X-ray absorption spectroscopy (soft-XAS) is limited by the high absorption cross sections of C, N, and O atoms, which reduce the penetration depth of soft X-rays compared to hard X-rays. However, soft-XAS can provide insights into the hydrogen-bonded structure of liquid water and the local structure of aqueous solutions, allowing the study of solute organic molecules at the C and N K-edges and solvent water at the O K-edge. It can also distinguish functional groups with similar elements based on energy differences in the soft-XAS peaks. Zheng et al. performed in situ soft-XAS to explore the electronic structure changes in CeO_2_/Ni–G during the N_2_ photoreduction process. In situ soft-XAS revealed the dynamic interfacial charge transfer of photogenerated electrons under illumination and the resulting charge accumulation at the catalytic active sites from CeO_2_ migrating toward Ni sites via rGO substrates, which is essential for N_2_ activation.^181^

In situ/operando soft-XAS and hard-XAS techniques are essential for characterizing the electronic and structural properties of SACs, offering valuable insights into their dynamic evolution, reaction mechanisms, and structure-performance relationships. However, challenges such as reactor design, signal interference, and poor signal-to-noise ratios under in situ/operando conditions limit the accuracy and comparison of data. To fully understand the intrinsic properties of SACs, continuous improvements in XAS methods, along with complementary characterization techniques and theoretical simulations, are necessary. Optimizing synchrotron beam stability, enhancing experimental conditions, and developing advanced synchrotron radiation technologies are critical for advancing the study of SACs.

#### 3.6.2 In Situ EPR

Combined in situ EPR and electrochemical setups have proven effective in a few recent studies representing the frontier of this field. This approach is particularly effective for quantifying oxygen vacancies and defects in electrocatalysts, as well as analyzing solid-phase processes under varying temperature conditions.^182^ A very recent development was introduced by Seif-Eddine et al. to use operando film-electrochemical EPR to enable real-time mechanistic studies of the electrocatalysts under reaction conditions (Figure 6b).^183^ By sensitively and quantitatively detecting paramagnetic species, such as radicals, EPR provides detailed insights into catalytic processes, exemplified here through alcohol oxidation catalysis using a surface-immobilized nitroxide. The study reveals that the surface electron-transfer rate, comparable to the catalytic rate, limits efficiency, offering valuable guidance for designing improved surface-immobilized catalysts. Integrating EPR with complementary techniques like XAS, UV–vis, and IR enhances real-time understanding of active-site structures and dynamics during catalytic cycles, while standardized protocols can broaden its accessibility and impact on designing sustainable catalytic technologies. However, in situ EPR has limitations such as the need for specialized instrumentation, and potential interference from surrounding environments. Additionally, isolating weak intensity signals of SAs is also a major challenge.

#### 3.6.3 In Situ FTIR

In situ synchrotron infrared characterization technology offers significantly higher photon intensity and a broader frequency range compared to traditional thermal radiation sources. Synchrotron-based FTIR can be used in transmission or reflection modes to obtain molecular structural information, with transmission mode preferred for gas–solid phase reactions and reflection mode often used for solid–liquid interface reactions in electrochemical processes. To reduce water molecule interference in electrochemical studies, techniques like the attenuated total reflection method and surface-enhanced infrared spectroscopy are employed. However, challenges exist in maintaining an authentic electrochemical environment during in situ measurements. To mitigate these issues, methods like controlling water layer thickness and adjusting the angle of the infrared light source are used. Synchrotron-based FTIR primarily focuses on the molecular fingerprint region (350–1500 cm^–1^) and functional group region (1500–4000 cm^–1^), providing valuable insights into reaction dynamics at the electrode surface, especially for oxygen- and hydrogen-involving intermediates.^183^

In OER studies, in situ FTIR is commonly employed to monitor the evolution of intermediates such as *O and *OOH, offering valuable insights into the reaction pathways and rate-determining steps of the reaction process. Recently, Su et al. developed an in situ FTIR setup to study the kinetic evolution during the OER. In acidic OER, the key intermediate *O, coordinated to a Co SA via heterocyclic nitrogen, forms when a voltage above 1.38 V is applied. Additionally, the Co–N absorption band shifts from 1048 cm^–1^ to 978 cm^–1^, indicating electron transfer from the Co atom to the adjacent N atom, which facilitates the formation of the *O intermediate at the catalyst’s true active site.^184^ Another example is using in situ FTIR to study nitrate reduction for electrochemical NH_3_ synthesis, where understanding its complex eight-electron transfer mechanism remains challenging. In situ XAS and FTIR offer real-time insights into catalyst surface species, intermediate formation, and reaction pathways, enabling continuous observation of nitrate reduction to NH_3_ and key intermediates like *NO_3_, *NO_2_, *NO, and *NH_2_. Xu et al. conducted a study on the dynamic evolution of Fe–N/P–C SACs for nitrate (NO_3_^–^) to NH_3_ reduction reaction (NO_3_RR), utilizing synchrotron-based in situ XAFS and FTIR (Figure 6c).^185^ FTIR measurements indicate the initiation of NO_3_^–^ reduction at −0.2 V and track the evolution of intermediates, such as *NO_2_^–^ and *NH_4_^+^, which intensify with increasing voltage and time, confirming the efficient progression toward NH_3_ production. This work highlights the critical role of the symmetry-broken Fe–N/P–C structure in enhancing Faradaic efficiency for NH_3_ production. While in situ FTIR is employed as a crucial tool for exploring the dynamic behavior of SACs in realistic environments the requirement of sophisticated experimental setups and careful interpretation of data, limits its wide usage among researchers.

#### 3.6.4 In Situ Raman Spectroscopy

The in situ/operando Raman technique provides molecular-level fingerprint information on the surface of materials, intermediates, and solvents under working conditions. It is an ideal tool for monitoring changes at the electrode–electrolyte interface. Additionally, specialized Raman techniques, such as surface-enhanced Raman spectroscopy (SERS), shell-isolated nanoparticle-enhanced Raman spectroscopy (SHINERS), and tip-enhanced Raman spectroscopy (TERS), have been developed to enhance surface sensitivity and selectivity, enabling the detection of trace interfacial intermediates.^186^ Raman spectroscopy is based on the inelastic scattering of light, which detects characteristic molecular vibrations. During the scattering process, molecules’ vibrational or rotational modes are either excited or relaxed, leading to energy shifts in photons. These shifts provide valuable information about the vibrational modes of the sample. Each molecule’s unique fingerprint Raman spectrum can be collected for both qualitative and quantitative analysis, due to its distinct vibrational energy levels. Raman spectroscopy offers several advantages: (1) the minimal scattering effect of water ensures no interference; (2) catalysts can often be tested directly in the reactor through transparent glass without the need for pretreatment; (3) functional groups with high symmetry and low polarity are especially suited for Raman spectroscopy; and (4) Raman spectroscopy excels in detecting signals in the low-wavenumber region. However, the method has inherent limitations, including shallow penetration depth, reliance on external standards for accurate stress quantification, minimal stress-induced peak shifts, significant peak shifts caused by temperature variations and nonstoichiometry, and the broad, low-intensity peaks observed at elevated temperatures. These challenges hinder precise quantitative stress analysis and contribute to discrepancies across studies.^187^

Wie et al. employed SHINERS successfully to identify single palladium atoms (Pd SAC) on various supports and elucidated the nucleation process of Pd species, progressing from SAs to NPs. Furthermore, by conducting in situ studies, they demonstrated the hydrogenation of a nitro compound catalyzed by Pd SAC.^188^ Their findings demonstrated that, compared to NPs, SACs exhibit distinct catalytic behavior, significantly influencing the interaction between active sites and the nitro group, thereby altering the overall catalytic performance.

#### 3.6.5 Scanning Electrochemical Microscopy

In situ spectroscopic techniques, such as XANES, have demonstrated the ability to probe the dynamic oxidation states and localized structures of catalytically active sites during catalytic processes. However, there is still a lack of studies to interrogate the reactant-adsorbed single metal site models where both the adsorbates and active sites contain nitrogen, to elucidate the mechanisms of NO_3_RR. The rapid reaction rates of active intermediates pose additional challenges for detection and analysis. Moreover, these spectroscopic methods are not suitable for providing quantitative site-specific information or high time-resolution measurements of reaction kinetics. In contrast, substrate-interaction scanning electrochemical microscopy (SI-SECM), a coulometric titration-based technique, enables precise quantification of the charge transferred to the initial catalyst layer, offering a complementary approach to studying such systems.^189^ SI-SECM is a highly effective technique for examining the localized electrochemical activity of a sample in situ within a liquid environment. By utilizing the interaction between a microelectrode tip and the substrate, SI-SECM indirectly detects and characterizes specific molecules adsorbed on the substrate surface. This method enables precise quantification of charge transfer to an initial catalyst layer, offering valuable insights into reaction kinetics.

Li and co-workers utilized SI-SCEM to analyze the time-dependent site density of single-site Fe moieties with dynamic oxidation states during nitrate reduction and water dissociation under specific potentials. As illustrated in Figure 6d,^189^ a catalyst powder ultramicroelectrode (UME) was fabricated to serve as the substrate electrode, while a similar-sized Pt UME was positioned as the tip electrode, aligned approximately 2 μm above the substrate. The experimental setup utilized a solution containing KOH as the supporting electrolyte and ferrocenemethanol (Fc) as a redox mediator. During the titration process, Fc was oxidized to Fc^+^ at the tip electrode. The tip-generated Fc^+^ subsequently reacted with Fe­(II) and Fe(0) species in the catalyst, producing a positive feedback response on the tip current recorded by the instrument. Previous studies have indicated that NH_3_ oxidation on a Pt surface can lead to electrode dissolution. However, during control experiments, where a pure Pt substrate lacking Fe active sites was employed, no such current feedback was observed, regardless of the presence or absence of NH_3_. The charges measured at the tip across various potentials were calculated by integrating the current–time titration curves to identify oxidation state transitions of Fe–N_*x*_ moieties, with Fe­(III) reducing to Fe­(II) at 0.8–0.9 V and further to Fe(0) at 0 to −0.1 V. The Fe(0)–N_*x*_ state, resembling iron (0) porphyrins, exhibited efficient nitrate reduction but remains challenging to characterize due to its transient nature. Kinetic studies revealed high reactivity, with Fe­(II)–N_*x*_ and Fe(0)–N_*x*_ binding nitrate at rates of 0.057 and 1.24 s^–1^, respectively, outperforming Fe NPs. In contrast, Fe­(II) in NPs showed no activity, while Fe(0) facilitated nitrate reduction at a slower rate of 0.01 s^–1^. For HER, only Fe(0) demonstrated activity, with rates of 0.027 and 0.041 s^–1^ for Fe–N–C catalysts and NPs, respectively. These findings underscore the unique thermodynamic and kinetic properties of Fe–N_*x*_ SACs, enabling selective and efficient nitrate reduction without competing HER, offering a pathway for enhanced catalytic performance in reduction reactions.

In situ XAS, EPR, FTIR, Raman, and SECM face challenges in SAC characterization compared to NPs and bulk materials due to weak signals from low atomic concentrations, making detection and spectral interpretation difficult. SACs exhibit diverse coordination environments, leading to broadened or ambiguous spectra, unlike well-defined NP structures. In EPR, only paramagnetic SACs are detectable, while FTIR and Raman suffer from weak adsorbate signals and background interference from support. SECM has limited spatial resolution for isolated atoms, and in situ conditions can induce changes in SAC oxidation states, complicating analysis.

## 4 Theoretical Calculations

Understanding the properties and behavior of SACs at the atomic level is essential not only for rational catalyst design but also helps gather mechanistic insights into catalyzed processes. Theoretical calculations offer a powerful tool to investigate the stability of SACs, active site characteristics, and possible reaction pathways.^190,191^ Both stationary states^192^ as well as dynamic processes^193,194^ can be investigated by theoretical methods. In addition, computational methods such as time-dependent density functional theory (TD-DFT) can be employed to study excited states and photochemical reactions catalyzed by SACs.^195^

### 4.1 Models of Single-Atom Catalysts

The first challenge in SAC computing is selecting a model that accurately represents the real system while balancing computational efficiency (Figure 7a).^190^ The size of the model should be chosen to achieve the same concentration of metal species as determined experimentally. Due to the usually low concentration of SAs, quite large systems must be used, which makes modeling SACs a computationally challenging task. In principle, two kinds of models can be used: cluster and periodic (Figure 7b), whose pros and cons are discussed later. Besides the concentration of the SA species, particular attention should be paid to their oxidation state, mode of immersion into the lattice, and coordination sphere. The oxidation state is usually derived from experimental methods like high-resolution XPS. It is worth noting that the validity of the computational model can be verified by calculating the XPS spectra and comparing it with experimental data.^196,197^ This strategy is highly recommended for modeling SACs because it can not only verify the model’s validity but also support XPS assignments.

**Figure 7:**
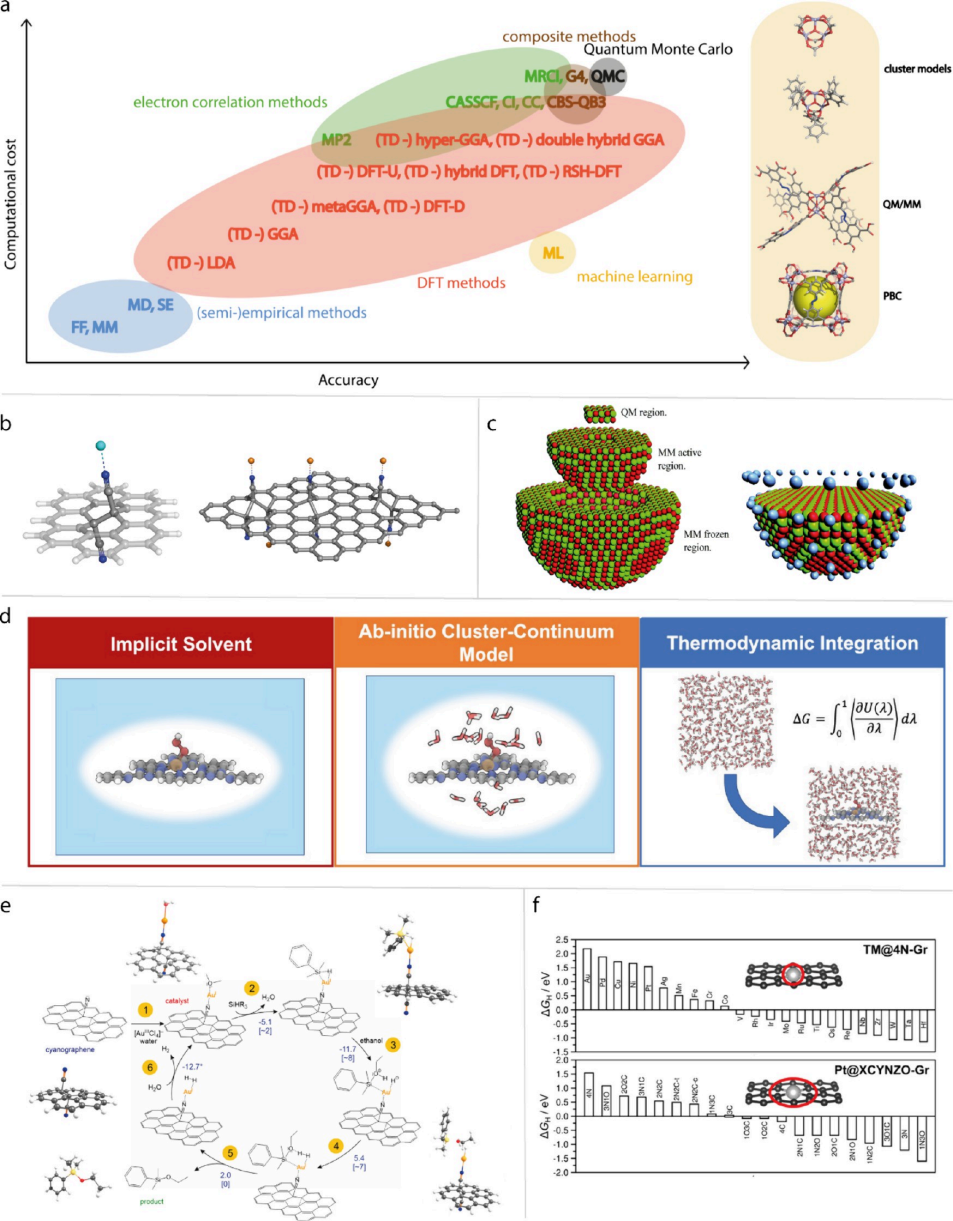
**Theoretical calculations for SACs.** (a) Computational methods that are commonly applied in SAC calculations. (b) Cluster (left) and PBC (right) models of metal SA supported by cyanographene. (c) Pictographic illustration of (left) a QM/MM model of the MgO(100) surface and (right) the complete QM/MM model of the MgO(100) surface including electrostatic correction charges outside the MM region. Reproduced with permission from ref 198. Copyright 2020 Royal Society of Chemistry. (d) Schematic illustration of the implicit model of solvent (left), the hybrid model of solvent (center), and the explicit model of solvent (right) that were used in ref 199. (e) Reaction mechanism of the dehydrogenative coupling of silanes catalyzed by Au atoms anchored on cyanographene that was proposed in ref 200. (f) Influence of the anchoring transition metal atom (top) and the configuration of the anchoring site (bottom) on Gibbs free energy of H adsorbed on the transition metal. Reproduced from ref 201. CC BY 4.0.

Information about detailed structural features of SA anchoring in the support matrix is usually not available, and therefore various binding modes of SA to different structural features of the matrix must be considered.^202,203^ In many studies, only one (dominant) binding mode is usually considered, e.g., binding of SA into triazine or heptazine hollows of g-C_3_N_4_.^204–207^ However, in practice, even such regular materials like graphene contain various defects (e.g., structural defects including Stone–Wales defects, or chemical defects including attachment of various functional groups like −OH, =O, etc.). Such defects usually represent high-surface-energy sites,^208^ and therefore SA species can preferentially bind to such sites. For realistic modeling of SACs, one should also consider binding into such defects. The realistic model should then consider the number of such high-energy sites (i.e., their saturation by SA), the strength of binding to matrix surface features, and also SAC preparation. The synthesis of SACs can be either a kinetically or thermodynamically driven process, which would naturally affect the final catalyst structure. It is also worth noting that the catalyst’s structural features may change during operation, and particularly for high-temperature processes, this feature should be taken into account. If the support can offer various binding features, e.g., those having the same coordination framework but differing in chemical composition, they should also be explored (Figure 7f).^201–203^ A prototypical example is a defective site in N-doped graphene, as such a site can be made up of four C atoms, three C atoms, and one N atom; two C atoms and two N atoms (in three arrangements); one C atom and three N atoms; or four N atoms. Similarly, the surface of MXenes can contain various surface-terminating groups, including −F and −OH, and their arrangement over the MXene surface offers a vast structural space that can house SA species in various coordination spheres made by the surface functional groups. Finally, the coordination sphere of SA species may also contain additional ligands, e.g., originating from preparation methods or solvents. Typically, high oxidation states of metal SA species usually prefer hexacoordination to saturate the high oxidation state. Realistic modeling of SACs must take this feature into account.

The cluster model is a molecular system consisting of some part of the support material with the active site and adsorbed SA species (Figure 7b). Such models are very attractive because they can benefit from theoretical methods developed for molecular systems over the last decades. A full arsenal of methods based on wave function theory (WFT), including post-Hartree–Fock methods (e.g., Møller–Plesset (MP) perturbation methods, coupled cluster (CC) methods, and various flavors of multireference methods such as configuration interaction (CI), complete active space (CAS), etc.), can be applied, along with a wealth of methods stemming from DFT and recently emerging methods based on machine learning (ML) potentials (Figure 7a). So far developed protocols and strategies for modeling metal-containing molecular systems can be adopted here and the interested reader is advised to follow the literature in this field.^191,197,199,209^ However, cluster models may not represent SACs well because they are limited in size and may introduce artificial edge effects and significant deformations compared to the real system. Furthermore, the termination of cluster edges must be carefully assessed to avoid introducing spurious states into the electronic structure that do not represent the actual physical or chemical properties of the material. These unwanted states can distort calculations of electronic structure, i.e., band gaps, density of states, and reactivity. To mitigate the influence of dangling bonds at the edges of cluster models, several strategies can be employed. One of the simplest and most widely used methods is hydrogen passivation, where the dangling bonds are saturated with hydrogen atoms (or small functional groups such as methyl groups). This approach was employed in computational studies of graphene-like materials,^200,210–212^ MOFs,^213,214^ and even of some metal oxides.^215^ In addition, mechanical embedding methods can be applied, where the region of interest is embedded within a larger model that simulates the bulk material (Figure 7c). This approach, called quantum mechanics/quantum mechanics (QM/QM) or quantum mechanics/molecular mechanics (QM/MM), treats the core part of the system with high accuracy using ab initio methods, while the edge regions are handled using more computationally efficient methods such as semiempirical DFT methods or molecular mechanics methods, i.e., based on a cheap empirical potential also known as force field.^190,191,216,217^ These methods have been applied in modeling catalytic reactions of zeolites^218–220^ and MCM-41.^221,222^ Alternatively, the influence of the surrounding environment can be accounted for by introducing an external electrostatic field derived from the charges in the surrounding region, ensuring a more accurate simulation of the system’s electronic interactions. This approach, called electrostatic embedding, was used for example in calculations of catalytic reactions over metal oxides.^198,223^ While both embedding approaches offer a better description of long-range electronic effects, they inevitably increase computational demands. It is worth noting that QM/MM methods can be also used to capture the dynamic evolution of solvent configurations and their impact on reaction pathways. Other methods for incorporating environmental effects into calculations will be discussed later. To ensure the reliability of the results, it is crucial to carefully select the cluster model, optimize its size through additional calculations, and, when possible, compare the results with those from periodic models.

Periodic models offer an alternative to the cluster models (Figure 7b). They consist of a supercell that is repeated in space under periodic boundary conditions (PBC). Such models naturally capture long-range interactions and SAC bulk properties. A wealth of DFT-based computational methods emerging from Bloch’s theorem provides a sufficient basis for electronic structure calculations, allowing the derivation of important features relevant to both SACs and the catalytic process. Recently, ML potentials have been introduced to this field. However, calculations of charged systems under PBC can be challenging, complicating the study of reaction mechanisms involving charged species. Periodic models are typically used for systems where the anchoring site is a structural defect, such as in heteroatom-doped carbon materials^224–226^ or in metal-based supports.^227–229^ The advantages and disadvantages of both approaches have been discussed in detail for graphene and oxides.^230–232^

The active sites of SACs are susceptible to structural transformations during catalytic processes, especially under high-temperature conditions. These transformations can lead to active site reconstruction due to the migration of SAs, defects, etc. Such changes can profoundly influence not only the structural but also the electronic properties of the catalysts, altering their interaction with the reactant system. The SAs can also undergo side reactions, which can modify their chemical properties, e.g., oxidation/reduction, and coordination of reactive species formed during reaction, just to name some examples. Temperature-induced diffusion of SAs may result in their aggregation, leading to the in situ formation of NCs or NPs.^194^ These aspects are crucial for understanding the behavior of SACs under operational conditions and must be considered when evaluating their performance and stability. These features must be taken into account when addressing SACs. Theoretical concepts were developed to investigate these dynamic processes and recently overviewed in literature.^193^

### 4.2 Methods to Study Reaction Mechanisms over Single-Atom Catalysts

Evaluation of reaction mechanisms (an example is in Figure 7e) in terms of reaction coordinates requires an accurate description of both thermodynamics and kinetics. In thermodynamics, i.e., calculations of reaction enthalpies (Δ_r_ *H*) or Gibbs energies (Δ_r_ *G*), the challenge lies mostly in describing non-covalent interactions between the catalyzed reaction system (reactants, intermediates, transition states, and products) and SACs. These non-covalent interactions include electrostatic, induction (polarization), dispersion (London), and exchange (repulsion) forces. Correctly describing dispersion interactions, which involve nonlocal electron–electron correlation effects, is particularly challenging because many DFT methods may fail to accurately describe this physical phenomenon, which often plays an important role in the catalyzed reaction.^233^

Accurately accounting for dispersion forces requires adequate theoretical approaches, such as post-Hartree–Fock methods at a correlated level or DFT methods equipped with empirical dispersion corrections (D) or nonlocal electron–electron correlation terms (e.g., vdW-DF).^199,225,234^ The WFT methods, such as the coupled-cluster technique are robust and accurate, and CCSD­(T) is considered the gold standard in the field of molecular modeling. However, they are prohibitively computationally demanding and are often used only for benchmarking other, less computationally demanding methods, on a small test system (containing usually less than 100 atoms). DFT-D or vdW-DF methods, on the other hand, offer a reasonable compromise between accuracy and computational efficiency and are the most widely used methods.

ML potentials represent an alternative and very attractive approach that can provide computationally affordable predictions of thermodynamics.^30,235,236^ Recent advancements in ML techniques (such as neural network potentials, Gaussian approximation potentials, and graph-based models) have significantly improved their applicability in modeling complex systems like SACs. These methods have been successfully applied to model large systems and explore reaction pathways with computational costs significantly lower than traditional ab initio methods. However, the accuracy of ML potentials heavily depends on the quality and diversity of the training data set. These methods tend to operate within or very close to their training set, and if the training set does not adequately cover the relevant configuration space, the ML model may yield inaccurate predictions when extrapolating beyond the training data. Therefore, their utilization should be cross-validated by adequate DFT or WFT methods. Recent studies have focused on improving training algorithms and incorporating active learning strategies to systematically expand the training set and enhance the model’s transferability. Moreover, researchers are developing hybrid approaches that combine ML potentials with quantum mechanical calculations to balance computational efficiency and accuracy. For instance, ML potentials can be used to rapidly scan potential energy surfaces, while critical points are refined using high-level DFT or WFT methods. Another emerging application of ML potentials is in the study of dynamic catalysis, where the active site can evolve under reaction conditions due to processes such as SAs aggregation or fragmentation of larger particles. In such cases, large-scale simulations enabled by ML potentials can help capture these transformations, providing insights into the real active site configurations during catalysis.^235,237,238^ By integrating ML-driven approaches with advanced sampling techniques and quantum mechanical validation, researchers can better understand the dynamic nature of SACs and refine catalyst design strategies.

Despite these advancements, it remains crucial to cross-validate ML potential predictions with reliable quantum mechanical calculations to ensure their reliability, especially when investigating new or complex reaction mechanisms or underexplored regions of the potential energy surface.

For the evaluation of reaction kinetics, an accurate description of the transition state (TS) is crucial. TSs must be localized, and their energies estimated with reasonable accuracy (within a few kJ mol^–1^). Localization of TSs is not an easy task because a TS cannot be characterized solely as a stationary point on the potential energy surface (PES) by vanishing first spatial derivatives, but the curvature of PES around the TS must also be probed. TSs are stationary points on the PES that have just one negative vibrational frequency, i.e., one downhill direction connecting the valleys of reactants and products on the PES. It should be noted that probing the stationary point curvature requires the computation of the Hessian matrix (of spatial second derivatives), which is a computationally demanding task. Localization of TSs follows the localization of reactants, intermediates, and products along the reaction pathway. Specialized techniques such as linear and quadratic synchronous transit (LST and QST) and nudged elastic band (NEB) methods are commonly employed to locate TSs. It is worth noting that reliable evaluation of TS energies must take into account that the electron densities in TSs are usually more delocalized than in reactants or products. Therefore, TS calculations may suffer from self-interaction error (SIE), necessitating the use of correlated WFT methods, hybrid functionals, or density functional theory with Hubbard corrections (DFT+U) to achieve reasonably accurate results.^199,225,234^

### 4.3 Environmental Effects

SACs catalyze a wide spectrum of chemical reactions across various phases, with gas and liquid phases being predominant. While reactions in the gas phase can often be modeled without considering environmental effects (i.e., in a vacuum) without significant loss of precision, modeling reactions in the liquid phase presents additional challenges due to solvent effects. The solvent provides electrostatic screening and can significantly influence reaction mechanisms and kinetics. To account for these effects, methods like the polarizable continuum model (PCM),^239–241^ the conductor-like screening model for real solvents (COSMO-RS),^242–244^ and the solvation model based on density (SMD)^245^ have been developed to enable polarization of the wave function or electron density, reflecting the solvent’s dielectric constant. In cases where the solvent actively participates in the reaction, such as facilitating proton transfer through specific hydrogen-bond networks, explicit inclusion of solvent molecules becomes essential (Figure 7d).^246–248^ Hybrid models that combine implicit and explicit solvation techniques are employed to balance computational efficiency with accuracy, treating solvent molecules explicitly near the reactive site while representing the bulk solvent implicitly. As mentioned above, techniques such as molecular dynamics (MD) simulations and QM/MM methods can capture the dynamic evolution of solvent configurations and their impact on reaction pathways. MD simulations can be used to probe solvent behavior close to the SAC catalytic site and to select representative model(s). The multiscale QM/MM method represents a reasonable compromise when a complex environment close to the SA catalytic site must be considered.

It is necessary to mention that SACs can proceed under an applied voltage and variable pH conditions which can cause dramatic changes in electrochemical reactions.^201,203^ In modeling electrochemical processes, the choice of reference states is crucial.

In electrochemistry, commonly referenced electrodes such as the normal hydrogen electrode (NHE), standard hydrogen electrode (SHE), and reversible hydrogen electrode (RHE) are pivotal for establishing standard potentials to benchmark reaction energies. These reference electrodes facilitate a uniform basis for comparing electrochemical potentials across different systems. The computational hydrogen electrode (CHE) model,^249^ frequently used in computational studies, standardizes electrode potentials relative to these hydrogen electrodes, enabling the theoretical evaluation of reaction energetics under consistent conditions. It is commonly used to simplify electrochemical calculations by not explicitly including protons or electrons.^250–255^ Despite its utility, the CHE model has several limitations. It operates under an assumption of constant charge instead of constant potential, and it tends to oversimplify the effects of the electrolyte. It also neglects changes in charge distribution and does not account for dynamic potentials, pH variations, or the specific site behaviors crucial in complex systems such as SACs. Additionally, the model does not consider electrode polarization, which can significantly affect reaction kinetics and the stability of intermediates.

To address these deficiencies, advanced computational methods have been developed. Grand canonical density functional theory (GC-DFT) offers an improved framework by simulating reactions at constant potential and allowing for electron exchange with the electrode, effectively incorporating more realistic conditions.^256^ Another method, the constant-potential embedded cluster model (CP-DFT), accounts for polarization effects and the interactions between the electrode and electrolyte, providing a more comprehensive analysis of electrochemical systems.^257^ These techniques have been applied to model key electrocatalytic reactions, such as the ORR^258^ and CO_2_RR,^259^ yielding insights that are closer to experimental conditions and more applicable to real-world scenarios. For dynamic and explicit modeling of solvent and ion effects, ab initio molecular dynamics (AIMD) is used to simulate solvent reorganization, ion migration, and proton transfers that are often overlooked in static DFT calculations.^260^ Additionally, methods like metadynamics and umbrella sampling explore reaction-free energy landscapes, providing a deeper understanding of activation barriers under electrochemical conditions.^261^ Moreover, the integration of ML with DFT is emerging as a powerful tool to accelerate and refine the prediction of reaction energies under electrochemical conditions, helping to balance accuracy with computational efficiency.^262^ These advancements are proving crucial for studying electrocatalytic processes, particularly in the context of innovative catalysts such as SACs.

All in all, computational chemistry plays a pivotal role in advancing the field of SACs. Emerging techniques, such as ML and artificial intelligence, are enhancing the predictive power of computational chemistry by accelerating material screening processes. Future challenges include improving the accuracy of theoretical models to better capture long-range interactions, charge transfer, and dynamic effects. The integration of experimental and computational data will also be critical for validating models and refining predictions. As computational power increases and methodologies evolve, computational chemistry will remain an indispensable tool for developing next-generation SACs tailored to address global energy and environmental challenges.

## 5 Applications of Single-Atom Catalysts in Electrocatalysis

This section explores the application of SACs in various electrocatalytic processes by presenting selected, relevant studies, emphasizing their design principles, performance metrics, and underlying mechanisms. By harnessing the unique advantages of SACs, researchers aim to address energy challenges and pave the way for efficient, scalable, and environmentally friendly electrocatalytic solutions.

### 5.1 Key Performance Metrics

The performance of SACs in electrocatalytic processes is assessed using several critical metrics that provide insight into their activity, efficiency, selectivity, and stability:

- Overpotential (η) [V]. It measures the additional voltage required to drive a reaction beyond its thermodynamic potential. For several processes, including OER and HER, the overpotential at a specific current density (e.g., η_10_ at 10 mA cm^–2^) is a key indicator of the catalytic efficiency;
- Tafel slope [mV dec^–1^]. Representing reaction kinetics, the Tafel slope reflects the relationship between overpotential and current density: a lower Tafel slope indicates that less overpotential is required to obtain a higher current;^263^
- Turnover frequency (TOF) [s^–1^]. The TOF quantifies the number of product molecules generated per active site per unit of time, offering a measure of the intrinsic catalytic activity independent of material loading;
- Mass activity [A g^–1^]. This parameter denotes the catalytic current generated per unit mass of the active material, highlighting the efficiency of the utilization of the catalyst. A high mass activity signifies better cost-effectiveness;
- Faradaic efficiency (FE). It indicates the fraction of the total current used for the desired reaction, reflecting the selectivity of the catalyst. High FE values are essential for minimizing side reactions and maximizing the efficiency of the process;
- Stability. It reflects the ability of a catalyst to maintain its performance over extended operation. It is typically evaluated through long-term chronoamperometry or cyclic voltammetry tests, where steady activity and minimal loss of performance over time indicate robust durability.

These metrics collectively enable a comprehensive evaluation of SAC performance across the various electrocatalytic reactions discussed in the following subsections.

### 5.2 Water Splitting

#### 5.2.1 Oxygen Evolution Reaction

Electrochemical water splitting has gained significant attention as a clean and efficient method to sustainably produce green energy by decomposing water into hydrogen fuel (H_2_) and oxygen (O_2_).^264^ This process consists of two half-reactions: the HER at the cathode, where H_2_ is produced, and the OER at the anode, where O_2_ is generated. While HER involves a straightforward two-electron transfer, OER is a complex four-electron transfer reaction, making it inherently sluggish and energetically demanding.^265^ The latter represents a major bottleneck of the water splitting reaction, which often requires high overpotentials, representing the additional energy required to drive the water splitting reaction compared to the thermodynamic value of 1.23 V, especially in acidic conditions.^266^ Therefore, developing catalysts that can accelerate OER while maintaining stability in harsh environments is essential to advancing this technology.

Traditional catalysts for water splitting include noble metals, such as Pt for HER and IrO_2_ or RuO_2_ for OER, due to their high activity and durability.^13,267,268^ However, their scarcity and high cost have driven the interest toward the development of alternative catalyst materials that can deliver similar performance with greater abundance and affordability. Oxides and hydroxides based on transition metals like Co, Ni, and Fe have shown promise as OER catalysts, but their activity and stability are often far from those of noble metals, especially under acidic conditions.^269^

SACs have emerged as a revolutionary approach to address these challenges by maximizing the utilization of the catalyst since each atom can serve as an active site.^270^ This approach also enables superior catalytic properties, as the surrounding SA environment can be fine-tuned to favor desirable reaction pathways. In this context, traditional SACs for the OER in acidic conditions, such as Ru SAs, operate via the lattice oxygen mechanism (LOM) and adsorbate evolution mechanism (AEM), which are illustrated in the left and central panels of Figure 8a.^271^ The LOM is usually characterized by more favorable kinetics and involves O atoms from the catalyst’s structure, which react with adsorbed intermediates to produce O_2_. However, the involvement of lattice O often destabilizes the catalyst material, as continuous vacancy creation and refilling can lead to structural degradation over time. On the contrary, in the AEM, adsorbed O_2_ intermediates undergo sequential transformations and couple to form O_2_ without the participation of the O from the catalyst’s lattice. Therefore, the AEM ensures higher stability compared to the LOM. However, the process follows a “scaling relationship” between the adsorption energies of intermediates (*O, *OH, *OOH) which limits the catalyst’s efficiency, as adjusting one intermediate’s binding energy may proportionally affect the others.

**Figure 8:**
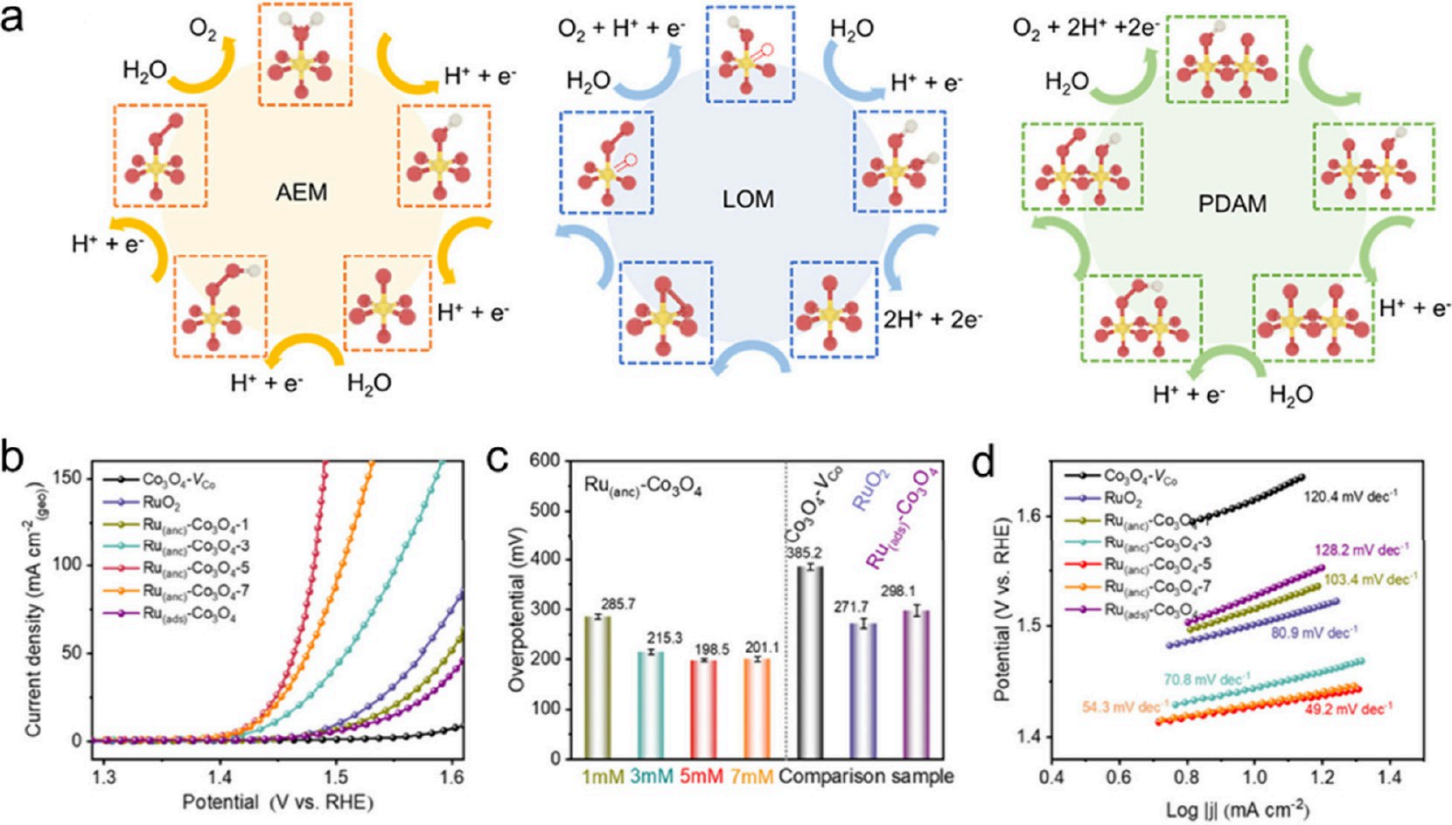
**Oxygen evolution reaction by SACs.** (a) Schematic diagram of the electrocatalytic mechanisms by Ru SAs on defective Co_3_O_4_, including the adsorbate evolution mechanism (AEM), lattice oxygen mechanism (LOM), and proton donor–acceptor mechanism (PDAM). (b) Polarization curves for defective Co_3_O_4_ (Co_3_O_4_-V_Co_), RuO_2_, lattice-anchored Ru (Ru_anc_-Co_3_O_4_-X, where X denotes Ru concentration), and surface-adsorbed Ru (Ru_ads_-Co_3_O_4_) in 0.5 M H_2_SO_4_. (c) η_10_ and (d) Tafel slope values. Reproduced from ref 272. Copyright 2023 American Chemical Society.

To address LOM and AEM limitations, a recent study by Hao et al. developed a novel SAC for the OER in acidic conditions by precisely positioning Ru atoms on defective Co_3_O_4_ supports to switch the reaction pathway from the LOM to a more efficient AEM.^272^ The controlled embedding of Ru atoms was a key aspect of this work, as it enabled tuning the specific interaction between Ru atoms and the surrounding lattice, switching the OER pathway. In particular, to understand the role of atomic positioning, they designed two configurations: lattice-anchored Ru (Ru_anc_-Co_3_O_4_), where Ru atoms are embedded within the cation vacancies in the Co_3_O_4_ lattice, providing a strong metal–support interaction, and surface-adsorbed Ru (Ru_ads_-Co_3_O_4_), where Ru atoms are loosely adsorbed onto the Co_3_O_4_ surface without being fully embedded. The performance of the two SACs was evaluated in 0.5 M H_2_SO_4_ (Figure 8b–d). First, compared to RuO_2_ or defective Co_3_O_4_, there is an outstanding improvement with Ru SACs. Specifically, Ru_anc_-Co_3_O_4_ showed an η_10_ of 198.5 mV, which is significantly lower than the 298.1 mV required by Ru_ads_-Co_3_O_4_, suggesting a higher catalytic activity for the lattice-anchored Ru SAs. The lower Tafel slope of 49.2 mV dec^–1^, recorded for the Ru_anc_-Co_3_O_4_, denotes a faster reaction kinetics. Nevertheless, the most impressive result was obtained for the mass activity, which was estimated to be 4012.11 A g^–1^ at 1.50 V vs RHE for Ru_anc_-Co_3_O_4_ and 36.96 A g^–1^ for Ru_anc_-Co_3_O_4_. This 100-fold increase originated from Ru_ads_-Co_3_O_4_ primarily following LOM, while Ru_anc_-Co_3_O_4_ promoted the AEM with a novel enhancement through the proton donor–acceptor mechanism (PDAM). The PDAM, depicted in the right panel of Figure 8a, involves efficient proton transfer, breaking the characteristic scaling relationship and allowing for lower energy barriers and faster reaction kinetics.

Apart from the mechanism, another factor affecting the performance of SACs in OER is the limited SA density. In fact, SACs often contain a low concentration of active metal atoms (typically around 1–1.5 wt %) which restricts their overall catalytic activity. Increasing the SA density poses challenges, as a higher metal content tends to cause agglomeration, where individual atoms cluster together into NPs, reducing the catalyst’s effective surface area and diminishing the benefits compared to atomically dispersed sites. To overcome these issues, Kumar et al. developed a high-density SAC with a Co loading of 10.6 wt %, embedded in a nitrogen-rich carbon framework.^273^ This material was created by a novel macromolecule-assisted synthesis method, employing cobalt phthalocyanine and a N-rich molecule, melem, to create a stable, high-concentration Co–N_4_ configuration within a graphitic carbon matrix. The strong coordination between Co and N atoms in the Co–N_4_ structure allowed Co atoms to remain isolated and anchored, even at high loadings, without clustering. This N-doped carbon scaffold provided a highly porous and conductive environment that not only stabilized the Co atoms but also improved electron transfer capabilities, essential for OER efficiency. Moreover, this design allowed Co atoms to exist in close proximity within the carbon matrix, enabling “cooperative catalysis,” where neighboring active sites interact to enhance catalytic performance. Specifically, the SAC exhibited an η_10_ of 351 mV and a Tafel slope of 84 mV dec^–1^ in 1 M KOH, demonstrating a notable electrocatalytic activity. Its mass activity reached 2209 A g^–1^ at 1.65 V vs RHE, which is exceptionally high for a non-noble metal catalyst. A high intrinsic catalytic activity was attested by a high TOF of 0.37 s^–1^ at 420 mV. It also remained stable over an extended testing period of more than 300 h.

A relevant work by Zhou et al. explored a novel strategy to enhance the OER efficiency of N-doped carbon materials hosting transition metal SAs (TM–N–C), such as the one described in the previous example.^154^ Usually, the performance of these catalysts is limited by challenging O–O coupling and strong *O adsorption on the transition metal center. To overcome these limitations, the researchers carbonized a P-doped Fe-ZIF-8 MOF, resulting in P atoms located in the vicinity of Fe active sites. The characterization of the synthesized material revealed that P creates a stable second coordination sphere, contributing to a higher surface area compared to the unmodified Fe–N–C, which is beneficial for the electrocatalytic activity. Indeed, this modification led to an η_10_ as low as 304 mV, a Tafel slope of 65 mV dec^–1^, and a TOF of 0.27 s^–1^ in 0.1 M KOH, comparable to the IrO_2_ benchmark, denoting a substantial improvement over Fe–N–C catalyst, which showed a higher η_10_ of 450 mV. Furthermore, DFT calculations suggested that P incorporation induces a local distortion around the Fe center, which adjusts the adsorption strengths of O intermediates (such as *OOH and *O), breaking the conventional scaling relation and enhancing the OER activity by balancing *OOH/*O adsorption energies.

In addition to the high η required for OER, another aspect worth consideration is that the water utilized for water splitting may be contaminated. However, wastewater has an intrinsic value, since it can contain substances whose involvement can contribute to reducing the η for OER, while simultaneously purifying the water. For instance, Luo et al. introduced a SAC designed for urea-assisted water splitting, thanks to the lower theoretical voltage necessary for urea oxidation.^160^ Once again, an N-doped carbon matrix was used to host Ni SAs by pyrolisis. The catalyst’s performance was notable since it obtained a current density of about 80 mA cm^–2^ at 1.4 V vs RHE in 1 M KOH in the presence of 0.5 M urea, surpassing Ni NPs onto the N-doped carbon matrix and commercial Pt/C, whose current densities were below 10 mA cm^–2^ at the same applied potential. It also exhibited the lowest Tafel slope of 40.9 mV dec^–1^ and the highest mass activity of 66500 A g^–1^ at 1.4 V vs RHE. This improvement was attributed to the Ni–N_4_ structure, which lowers the energy required for cleaving the C–N bond, the rate-limiting step in the urea oxidation reaction, and the reduced CO_2_ binding, which further improves the catalytic efficiency.

In recent years, computational methods, particularly DFT, combined with ML have emerged as powerful tools for the large-scale design and exploration of new SAC properties. For instance, Ha et al. combined DFT with ML algorithms to explore the stability and activity of SAs in N-doped graphene defect sites.^274^ Their findings revealed that specific N_2_C_2_ configurations exhibit better electrochemical performance and durability than others, uncovering new OER and ORR mechanisms. Similarly, Wu et al. demonstrated the synergy between computational predictions and experimental validation by incorporating transition metal SAs into N-doped carbon materials, significantly enhancing their OER catalytic performance.^275^ Their work highlights the importance of tuning the local coordination environment to optimize catalytic activity and stability. These interdisciplinary approaches collectively pave the way for the rational design of high-performance SACs.

#### 5.2.2 Hydrogen Evolution Reaction

SACs demonstrated to be effective also for HER. In this regard, RuO_2_ is considered among the most efficient catalysts for OER in alkaline conditions, while it presents insufficient HER activity when tested in acidic media. Nonetheless, by embedding Co SAs within a RuO_2_ matrix via a one-pot hydrothermal process, Shah et al. altered the electronic environment around Ru atoms, facilitating intermediate bonding and enhancing the catalytic performance for HER in acidic media.^156^ For HER, the catalyst showed exceptional activity with an ultralow η_10_ of 45 mV and a Tafel slope of 58 mV dec^–1^ in 0.5 M H_2_SO_4_, which is superior to benchmark catalysts, and strong stability after a 20 h continuous test. This performance was attributed to electronic interactions between Co and Ru that adjust the H^+^ binding energy on Ru sites, thereby accelerating the adsorption and desorption steps critical for HER efficiency. Tested also for OER, it achieved an η_10_ of 200 mV and a Tafel slope of 100 mV dec^–1^ in 1 M KOH. In this case, Co SAs contributed to enhancing RuO_2_ catalytic performance and preventing the overoxidation of Ru sites under high applied potentials, prolonging its lifespan.

The previous example introduced an approach to improve the limited electrocatalytic activity of RuO_2_ for HER in acidic conditions, where Pt-based electrodes are widely recognized as the most active catalysts. On the contrary, Pt catalytic efficiency in alkaline media is approximately 2 orders of magnitude lower than in acidic environments. This lower performance is attributed to the minor water dissociation ability of Pt atoms in these conditions, resulting in an insufficient supply of protons for H_2_ generation. Additionally, Pt is an expensive noble metal, and many efforts have been devoted to reducing Pt loading. To address this issue, a study by Zhu et al. proposed the development of a highly active and cost-effective catalyst for HER in alkaline media based on Ru/RuO_2_ heterostructure doped with atomically dispersed Pt.^150^ This SAC was obtained by chemically synthesizing a RuO_2_ enriched with grain boundaries, followed by impregnation with Pt atoms and a calcination process in the Ar atmosphere leading to Pt incorporation as SAs and slight reduction of RuO_2_ to Ru. The catalyst achieved ultralow values of 18 mV and 18.5 mV dec^–1^ for η_10_ and Tafel slope, as well as an impressive mass activity of 2227.3 A g^–1^ at an η of 63 mV in 1 M KOH, surpassing benchmarks such as Pt/C and Ru/C electrodes at a fraction of the cost (Figure 9a–c). DFT calculation attributed these results to the synergy between RuO_2_, which promotes water dissociation in the initial Volmer step of HER, and Pt SAs, which facilitate efficient proton adsorption. Moreover, the metallic Ru further contributed to facilitating subsequent H_2_ evolution steps, leading to improved HER efficiency. Of note, in an anion exchange membrane water electrolyzer, the catalyst required only 1.90 V to reach 1 A cm^–2^, demonstrating excellent efficiency, and remarkable stability over 100 h of continuous operation.

**Figure 9:**
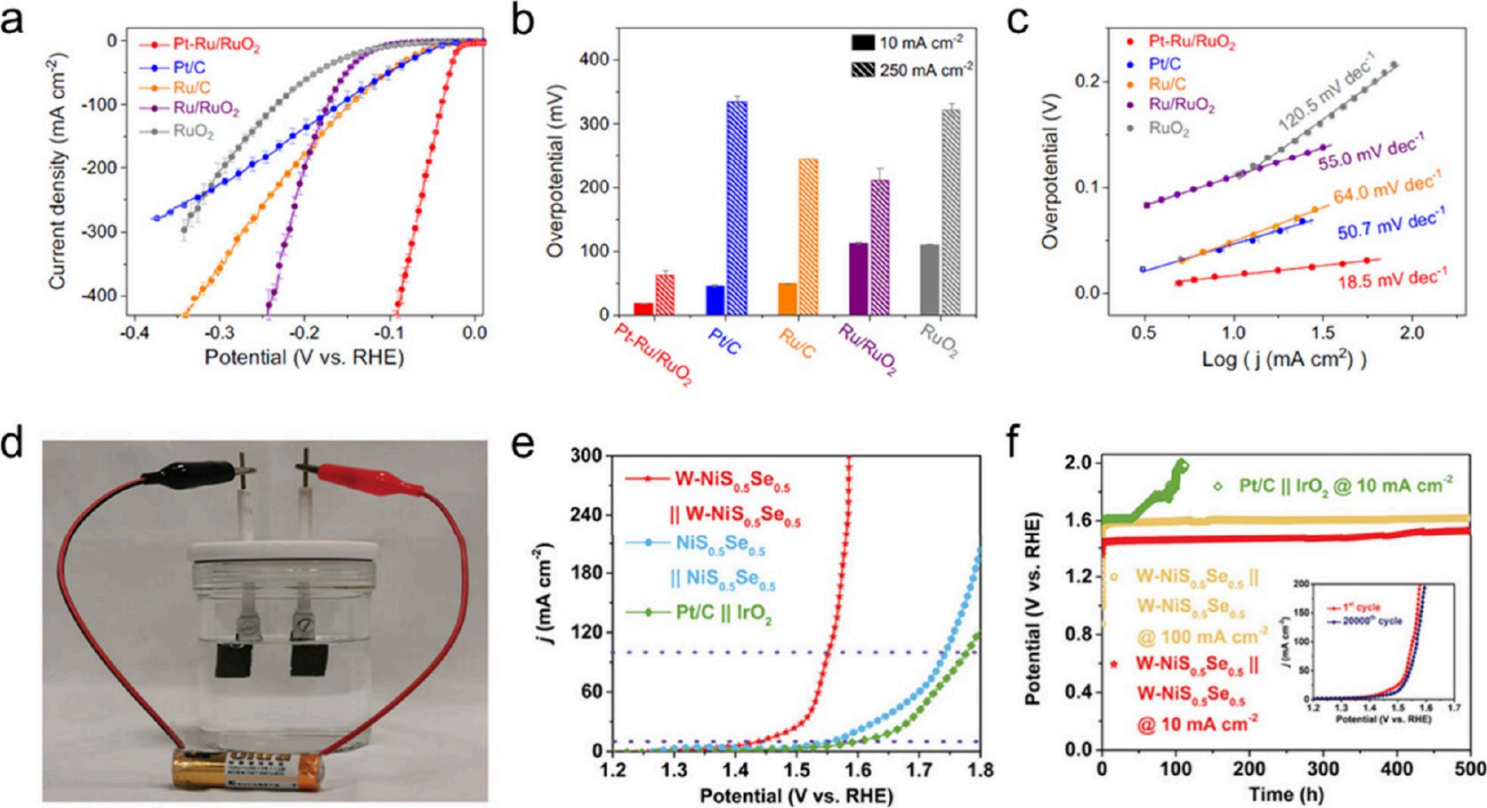
**Hydrogen evolution reaction by SACs.** (a) Polarization curves of RuO_2_, Ru/RuO_2_, Ru/C, Pt/C, and Pt SAs on Ru/RuO_2_ in 1 M H_2_SO_4_. (b) η and (c) Tafel slope values. Reproduced with permission from ref 150. CC BY 4.0. (d) Photograph showing the gas evolution of an electrolysis cell with W SAs on NiS_0.5_Se_0.5_ nanosheets@nanorods as both the cathode and anode, powered by a commercial 1.5 V AA battery. (e) Polarization curves of Pt/C||IrO_2_, NiS_0.5_Se_0.5_||NiS_0.5_Se_0.5_, W-NiS_0.5_Se_0.5_||W-NiS_0.5_Se_0.5_ in 1 M KOH. (f) Durability test by chronoamperometry at 10 mA cm^–2^. The inset shows the polarization curve of W-NiS_0.5_Se_0.5_||W-NiS_0.5_Se_0.5_ before and after 20000 cyclic voltammetry cycles. Reproduced with permission from ref 280. Copyright 2022 Wiley-VCH.

Depending on the nature of the SAC, its HER or OER efficiency is heavily influenced by the medium’s pH. Therefore, the development of SACs that overcome the limitations of the various catalysts under specific pH conditions is highly desirable. To face this challenge, Rong et al. designed a dual-site SAC capable of performing effectively both HER and OER across a wide pH range.^276^ Particularly, the Co–N_4_ sites increased the electron density around Ru atoms and enhanced Ru–O covalency, facilitating the adsorption and desorption of H_2_/O_2_ intermediates, crucial for efficient HER and OER processes. As a result, for the HER, the catalyst demonstrated an η_10_ of 13 mV, a Tafel slope of 23.3 mV dec^–1^ in 0.5 M H_2_SO_4,_ and a slightly worse activity in 1 M KOH (e.g., η_10_ of 23 mV), which is still among the best HER performances in the field. Furthermore, the catalyst’s long-term stability at various pH levels proved the enhanced resistance to corrosion provided by Co atoms. For OER, it reached an η_10_ of 232 mV and an outstanding TOF of 9.2 s^–1^ at 1.53 V vs RHE in 0.5 M H_2_SO_4_, as well as a low η_10_ of 247 mV in 1 M KOH, confirming its robustness across different environments. Additionally, it showed stable operation at a high current density of 450 mA cm^–2^ for 330 h in a proton exchange membrane water electrolyzer.

In a similar study, Zhang et al. presented a novel, pyrolysis-free synthesis method to produce a bimetallic Ni/Fe SAC embedded in a conjugated phthalocyanine framework (CPF), allowing electrochemical water splitting across a wide range of pH conditions.^277^ The CPF was employed to avoid the aggregation of the metal sites that commonly occurs during high-temperature pyrolysis, ensuring the good dispersion of the atoms. The metal atoms were coordinated to N in the stable Ni/Fe–N_4_ configuration, which enhances electronic interactions between Ni and Fe atoms but also optimizes the binding energies for H_2_ and O_2_ intermediates, promoting HER and OER in both acidic and alkaline solutions. In particular, the catalyst achieved low η_10_ but moderate Tafel slope values of 23 mV and 82.6 mV dec^–1^ for HER and 201 mV and 169.5 mV dec^–1^ for OER in 0.5 M H_2_SO_4_ and 42 mV and 91.4 mV dec^–1^ for HER and 194 mV and 102.1 mV dec^–1^ for OER in 1 M KOH. TOF analysis revealed high intrinsic activity per site, with the highest registered values of 3.16 s^–1^ at an η of 200 mV for HER in 0.5 M H_2_SO_4_ and 1.4 s^–1^ at an η of 400 mV for OER in 1 M KOH. Electrochemical durability tests reveal that it maintains stable performance for over 200 h without significant degradation, retaining its two-dimensional slice morphology and SAs dispersion.

The structural stability of SACs is considered a key performance parameter for both HER and OER, and identifying effective strategies to prevent their degradation has become increasingly important. In this context, Sun et al. designed a SAC to perform both HER and OER without deteriorating by anchoring Ru atoms onto a Ni–V layered double hydroxide (Ni–V LDH) by a facile hydrothermal reaction.^278^ The catalyst displayed an impressive HER activity, with a low η_10_ of 21 mV, a Tafel slope of 53 mV dec^–1^, a high mass activity of 6678 A g^–1^, and a TOF of 3.495 s^–1^ at an η of 100 mV in 1 M KOH, outperforming commercial Pt/C. For OER, it achieved an η of 227 mV, a low Tafel slope of 33 mV dec^–1^, a TOF of 0.143 s^–1^, and a mass activity of 931.3 A g^–1^ at an η of 300 mV, still better than the RuO_2_ benchmark catalyst. Despite this, it is the high stability that truly distinguishes its performance since it maintained consistent activity without any structural reconstruction or loss of catalytic sites even under 240 h electrolysis conditions. This result is due to the strong metal–support interaction between Ru and Ni–V LDH, with the Ni–V LDH stabilizing Ru sites so that they undergo small fluctuations in oxidation state during HER and, in turn, the Ru sites improving the structural tolerance of the Ni site during OER.

As an alternative to LDH, 2D materials like MXenes represent advantageous supports for SACs, providing both large surface area and metal-like conductivity that are crucial for improving HER and OER kinetics. For example, ul Haq et al. combined Fe–N–C SA sites with Nb_4_C_4_T_*x*_ MXene nanosheets, forming a composite material with remarkable efficiency for both HER and OER.^279^ For instance, it demonstrated η_10_ and Tafel slope values of 91 mV and 70.08 mV dec^–1^ for HER, and 290 mV and 42.44 mV dec^–1^ for OER in 0.1 M KOH. Importantly, experiments and simulations indicated that the MXene backbone prevents aggregation and enhances the stability of SA sites, in addition to improving the charge transfer and the adsorption of O_2_ intermediates, thus accelerating reaction kinetics.

Aiming at attaining both remarkable activity and durability for HER and OER, the study by Wang et al. investigated a novel water splitting electrocatalyst featuring W SAs doping a heterostructure of NiS_0.5_Se_0.5_ nanosheets grown on NiS_0.5_Se_0.5_ nanorods prepared by solvothermal reactions.^280^ This catalyst reached low η_10_ and Tafel slope values of 39 mV and 51 mV dec^–1^ for HER, and 171 mV and 41 mV dec^–1^ for OER in 1 M KOH, with TOF values of 0.21 s^–1^ at −130 mV vs RHE for HER and 0.056 s^–1^ at 250 mV vs RHE for OER. Owing to these metrics, the catalyst surpassed established benchmarks, such as Pt/C for HER and IrO_2_ for OER. This enhancement is attributed to the electronic interaction between W atoms and the NiS_0.5_Se_0.5_ support: W dopant modifies the electronic structure of the Ni atoms by inducing spin-state delocalization, which increases the d-electron density of Ni, ultimately facilitating better adsorption/desorption of reaction intermediates and improving reaction kinetics. DFT calculations revealed that W atoms optimize the adsorption energy for O_2_ intermediates, particularly during the *O → *OOH transition, which is often the rate-limiting step in OER. Additionally, the unique nanosheet-on-nanorod architecture enhances structural stability and accessibility to active sites, allowing electrolyte ions to penetrate and circulate freely within the structure, maximizing the utilization of the catalyst’s surface area. Besides, a two-electrode electrolysis cell with W-NiS_0.5_Se_0.5_ as both the cathode and anode was developed and tested (Figure 9d–f). This cell outperformed a NiS_0.5_Se_0.5_||NiS_0.5_Se_0.5_ cell, further demonstrating the superior catalytic activity enabled by SAs, and a benchmark cell with Pt/C||IrO_2_. Stability tests confirmed the catalyst’s robustness over 500 h or 20000 cyclic voltammetry cycles without significant degradation.

A recent computational study by Zhao et al. explored how asymmetric coordination environments in SACs can regulate HER performance.^281^ By utilizing heteroatom-doped graphdiyne as a support, they demonstrated that tuning the D-band structure and spin states of transition metals significantly impacts catalytic activity. Notably, Cr@N_B_1_-GDY exhibited Gibbs free energy changes close to zero, indicating superior HER performance. In the study of Song et al., an ML-DFT approach is used to assess the performance of chiral and achiral M-N-SWCNTs (M = In, Bi, Sb) SACs for HER.^282^ The authors demonstrated that chirality significantly influences the stability and catalytic activity of metal atoms, with right-handed In–N–SWCNT showing a 5.71-fold enhancement in HER activity compared to its achiral counterpart. The study attributes this enhancement to the symmetry breaking of spin density, which is analyzed using ML, revealing how chirality affects spin selectivity and electron transport efficiency. The study of Khossossi et al. combined DFT with few-shot learning to screen SACs supported on Ga_2_CoS_4–*x*_, a novel 2D material.^283^ They demonstrated that introducing surface sulfur vacancies significantly enhances HER activity, achieving overpotentials as low as 0–60 mV, comparable to or better than commercial Pt/C.

Table 1 summarizes the key performance metrics of the presented SACs for OER and HER in acidic and alkaline media. Taking into account the lowest η_10_ and Tafel slope values at first, for OER in acidic conditions the Ru_anc_-Co_3_O_4_ SAC demonstrates superior catalytic activity and efficiency (198.5 mV, 49.2 mV dec^–1^). In contrast, in alkaline conditions, W-NiS_0.5_Se_0.5_ SAC shows the best performance (171 mV, 41 mV dec^–1^). For HER in acidic conditions, Ru/Co–N_4_ SAC outperforms others (13 mV, 23.3 mV dec^–1^). Instead, in alkaline conditions, Pt–Ru/RuO_2_ SAC exhibits the best metrics (18 mV, 18.5 mV dec^–1^). Of note, W-NiS_0.5_Se_0.5_ SAC exhibits a remarkable HER activity in alkaline conditions (39 mV, 51 mV dec^–1^), positioning itself as one of the most promising candidates for overall water splitting.

**Table 1:** Key Performance Metrics of SACs for OER and HER

reaction	SAC	electrolyte	η_10_ [mV]	Tafel slope [mV dec^–1^]	TOF [s^–1^]	mass activity [A g^–1^]	ref
OER	Ru_anc_-Co_3_O_4_	0.5 N H_2_SO_4_	198.5	49.2	n/a	4012.11	^272^
	Ru/Co–N_4_	0.5 M H_2_SO_4_	232	n/a	9.2	n/a	^276^
	P-doped Fe–N–C	0.1 M KOH	304	65	0.27	n/a	^154^
	Fe–N–C Nb_4_C_4_T_*x*_	0.1 M KOH	290	42.44	n/a	n/a	^279^
	Co–N_4_	1 M KOH	351	84	0.37	2209	^273^
	Co-RuO_2_	1 M KOH	200	110	n/a	n/a	^156^
	Ni/Fe–N_4_	1 M KOH	194	102.1	1.4	n/a	^284^
	Ru- Ni–V LDH	1 M KOH	227	33	0.143	931.3	^278^
	W-NiS_0.5_Se_0.5_	1 M KOH	171	41	0.056	n/a	^280^
HER	Co-RuO_2_	0.5 M H_2_SO_4_	45	58	n/a	n/a	^156^
	Ru/Co–N_4_	0.5 M H_2_SO_4_	13	23.3	n/a	n/a	^276^
	Ni/Fe–N_4_	0.5 M H_2_SO_4_	23	82.6	3.16	n/a	^284^
	Fe–N–C Nb_4_C_4_T_*x*_	0.1 M KOH	91	70.8	n/a	n/a	^279^
	Pt–Ru/RuO_2_	1 M KOH	18	18.5	n/a	2227.3	^150^
	Ru- Ni–V LDH	1 M KOH	21	53	3.495	6658	^278^
	W-NiS_0.5_Se_0.5_	1 M KOH	39	51	0.21	n/a	^280^

### 5.3 Oxygen Reduction Reaction

The ORR is an essential electrochemical process crucial for the efficiency of various energy technologies, including Zn–air batteries and fuel cells.^285^ There are various possible ORR pathways based on the number of transferred electrons: a four-electron reaction pathway, in which O_2_ is directly reduced to H_2_O or OH^–^ in acidic or alkaline conditions, respectively, and a two-electron reaction pathway, where O_2_ is partially reduced to H_2_O_2_ or H_2_O in acidic or alkaline media, respectively. The preferred ORR pathway in these applications is the four-electron one. Zn–air batteries rely on OER for charging and ORR for discharging, converting chemical energy to electrical energy.^286^ These batteries are especially promising for grid storage and electric vehicles due to their high theoretical energy density and safety. In fuel cells, ORR occurs at the cathode, converting O_2_ and protons (from the anode) into H_2_O, which releases the stored energy in the fuel as electrical energy.^4^ However, ORR’s inherent kinetic barriers lead to sluggish reaction rates, motivating the need for the development of more efficient catalysts. Moreover, for practical applications, it is fundamental to reduce catalysts’ costs and improve their durability.

It is in this context that SAC offers the possibility to maximize the utilization of the active material, replacing expensive noble metal catalysts or reducing the fabrication costs, while contemporarily allowing enhanced efficiency and stability by precisely tuning the electronic environment around the atomically dispersed catalytic sites.^287^ In this section, a selection of original and impactful strategies to improve ORR by SACs is presented. To better understand the performance of SACs in ORR and Zn air batteries, the following metrics have to be introduced in addition to those described in section 5.1, allowing to better understand the ORR and Zn–air performance of SACs:

- Half-wave potential (*E*_1/2_) [V]. The potential at which the current density reaches half of its limiting value in ORR, reflecting the catalytic activity: a higher *E*_1/2_ indicates better performance;
- Kinetic current density (*j*_k_) [mA cm^–2^]. It denotes the current normalized to the unit area, measured after eliminating mass transport effects. It reveals the efficiency of the catalyst.
- Open circuit potential (OCP) [V]. It is the voltage of the Zn–air battery when no current flows, indicating the maximum thermodynamic potential;
- Peak power density [mW cm^–2^]. It is the maximum power per unit area the Zn–air battery can deliver, typically measured under optimized load conditions.
- Discharge specific capacity [mAh g^–1^]. Reflecting the energy storage efficiency of the Zn–air battery, this parameter represents the total charge the battery delivers during discharge, normalized to the mass of the Zn anode.

Many efforts have been devoted to enhancing ORR efficiency by modifying the active site configuration. In a pioneering study, Dai et al. explored the concept of d-orbital splitting to enhance the ORR activity of a Fe-based SAC using alkalized MXene nanosheets as a support.^288^ In particular, a quasi-octahedral coordination environment was engineered around the Fe–N_4_ site by introducing an additional O ligand, creating a Fe–N_4_O_1_ configuration. This modification induced a distortion in the typical square-planar Fe–N_4_ field, altering the electronic density distribution in the d orbitals of Fe. As a result, the desorption of ORR intermediates like *OH was enhanced, improving the catalytic activity of the catalyst. Indeed, it showed a higher *E*_1/2_ (0.924 V vs RHE), a lower Tafel slope (54.79 mV dec^–1^), a higher *j*_k_ (16.9 mA cm^–2^), TOF (0.555 s^–1^), and mass activity (953.2 A g^–1^) at 0.9 V vs RHE, as well as an excellent stability compared to the traditional Fe–N_4_ structure in 0.1 M KOH. Furthermore, it was used as a cathode for a Zn–air battery, which exhibited remarkable performance as an OCP of 1.487 V, a peak power density of 191.5 mW cm^–2^ at a current density of 282.1 mA cm^–2^, and a discharge-specific capacity of 779.8 mAh g^–1^ at 10 mA cm^–2^ with 95.1% utilization of the theoretical specific capacity.

On the contrary, Li et al. focused on the influence of N-doped mesoporous carbon as a support for Fe SAs.^289^ The study revealed that this design resulted in an enhanced dispersion and stability of Fe active sites. Additionally, the mesoporous carbon structure improved the mass transport while the N doping altered the electronic properties of Fe atoms, allowing for more effective adsorption and desorption of intermediate species. Consequently, a remarkable ORR activity was obtained with high *E*_1/2_ values of 0.93, 0.83, and 0.75 V vs RHE across alkaline, acidic, and neutral pH levels, respectively. In particular, in 0.1 M KOH, the improvement in catalytic activity and efficiency compared to control samples without SAs is remarkable (Figure 10a). In these conditions, it also showed a low Tafel slope of 77.7 mV dec^–1^ and a high *j*_k_ of 63.69 mA cm^–2^ at 0.8 V vs RHE, as well as a high stability. A Zn–air battery designed using this catalyst as a cathode achieved an OCP of 1.46 V, a peak power density of 306.1 mW cm^–2^, and a discharge-specific capacity of 746.9 mAh g^–1^ at 10 mA cm^–2^, corresponding to the 91.1% of the theoretical capacity (Figure 10b,c).

**Figure 10:**
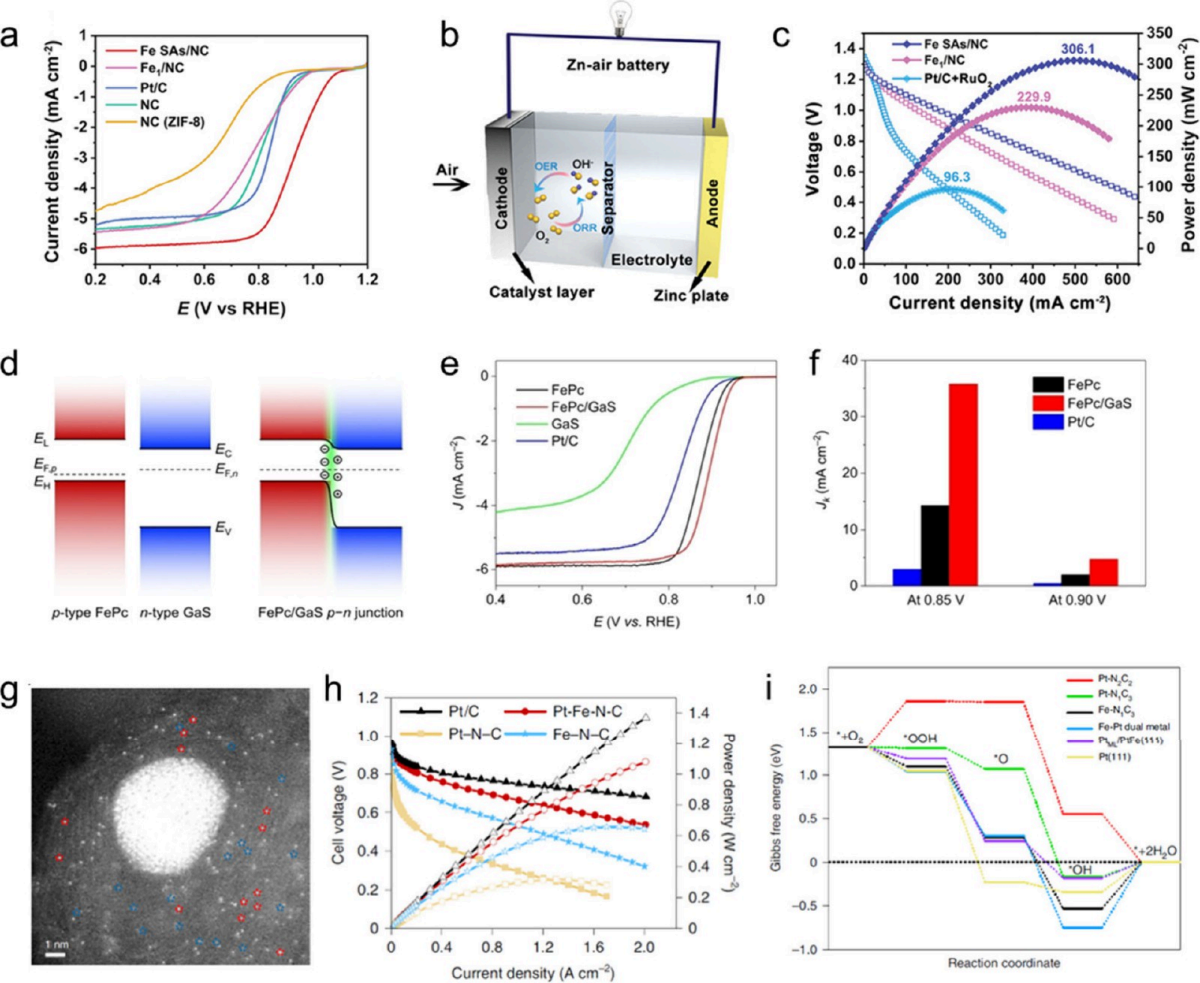
**Oxygen reduction reaction by SACs.** (a) ORR polarization curves of ZIF-8 MOF, N-doped carbon (NC), Pt/C, Fe_1_/NC, and Fe SAs on NC (Fe SAs/NC) in 0.1 M KOH. (b) Schematic illustration of a Zn–air battery. (c) Discharging polarization (hollow symbols and left axis) and power density (solid symbols and right axis) curves for Zn–air batteries with Pt/C+RuO_2_, Fe_1_/NC, and Fe SAs/NC as the cathode. Reproduced with permission from ref 289. Copyright 2022 Wiley-VCH. (d) Schematic illustration of interfacial charge redistribution in FePc/GaS p–n junction. (e) ORR polarization curves of Pt/C, GaS, FePc, and FePc/GaS in 0.1 M KOH. (f) *j*_k_ values at different potentials. Reproduced with permission from ref 294. Copyright 2022 Wiley-VCH. (g) HAADF-STEM image showing the coexistence of Fe (blue) and Pt (red) SAs near Pt–Fe NPs. (h) H_2_/O_2_ fuel cell polarization (solid symbols and left axis) and power density (hollow symbols and right axis) curves with loadings of 0.1 mg_Pt_ cm^–2^ for Pt/C and Pt–N–C and 0.015 mg_Pt_ cm^–2^ for Pt–Fe–N–C, and a 3.5 mg cm^–2^ catalyst loading for Fe–N–C in the cathode. (i) Gibbs free energy diagram of ORR on various possible active sites at 0.9 V. Reproduced from ref 300. CC BY 4.0.

To better understand the stability and dispersion of transition metal SACs, Wu et al. conducted a DFT study on defective carbon supports, identifying key factors that govern these properties.^290^ Their findings revealed that ORR activity is closely linked to the electronic structure and coordination environment of the metal SAs. By employing a few-shot ML model, the authors achieved a significant reduction in computational cost, over 130,000-fold compared to conventional DFT calculations, while maintaining a prediction error of 8.33%. In their innovative work, Li et al. combined DFT and ML to discover efficient electrocatalysts for H_2_O_2_ production.^291^ By utilizing Δ*G*(O) and Δ*G*(OOH) as descriptors within a contour map, they screened 150 N-doped graphene-supported metal SACs, identifying 21 systems with high selectivity and activity for H_2_O_2_ production. Further analyses, including constant-potential and kinetic studies, revealed that catalysts such as Pd−N_4_, Pt−N_4_, Ni−N_4_, and Cu−N_4_ exhibit selectivity for the two-electron ORR under both acidic and basic conditions, aligning with current experimental findings. Additionally, a compressed sensing data analytics approach was employed to uncover inherent correlations between structure and catalytic performance, enabling accurate predictions of descriptors and significantly reducing time and cost.

To achieve a high metal loading in SACs, Zhang et al. utilized a self-sacrificing template method to create Co SAs and clusters coanchored on N-doped graphene with 14 wt % Co loading.^292^ This method allowed Co atoms to remain stable and active, thanks to the interaction with the nitrogen dopants in the carbon support. On the other hand, the coexistence of Co SAs and clusters increased ORR activity, as confirmed by both experimental and DFT studies. Indeed, this catalyst showed high values for *E*_1/2_ (0.890 V vs RHE) and *j*_k_ (17.070 mA cm^–2^ at 0.85 V vs RHE) and excellent stability in 0.1 M KOH. In Zn–air battery applications, it exhibited an OCP of 1.5 V, a peak power density of 221 mW cm^–2^, and good stability following a rechargeability test.

Introducing C vacancies in Fe–N–C SACs proved to be an effective strategy to enhance ORR performance, as suggested by the study of Tian et al.^293^ Thanks to this innovation, the catalyst reached a high *E*_1/2_ of 0.91 V vs RHE, a low Tafel slope of 58 mV dec^–1^, a *j*_k_ of 7.00 mA cm^–2^, and remarkable durability over 100,000 cycles, with only a 29 mV loss in *E*_1/2_, in 0.1 M KOH. DFT calculations indicated that these vacancies lower the *OH adsorption free energy, stabilizing the metal center by reducing its dissolution and significantly enhancing ORR kinetics. Besides, in a Zn–air battery system, the catalyst showed excellent performance, with an OCP of 1.53 V, a peak power density of 230 mW cm^–2^, a discharge specific capacity of 814 mAh g^–1^ at 10 mA cm^–2^, and impressive stability over 1200 h.

Another innovative strategy to enhance ORR in SACs is through p–n junction modulation, as demonstrated by Zhuang et al.^294^ In this approach, an iron phthalocyanine (FePc) SAC with p-type semiconducting behavior was coupled with an n-type semiconductor GaS support. As indicated by experimental analysis and DFT calculations, the resulting p–n junction introduced a rectifying space-charge region at the interface, which facilitated continuous charge redistribution around the Fe–N_4_ active site (Figure 10d). The stabilization of the Fe center and the favorable spin-state transition induced by this charge rectification effect synergistically enhanced ORR kinetics and stability, as the catalyst displayed an *E*_1/2_ of 0.87 V vs RHE, higher than pristine FePc and Pt/C, and a doubled *j*_k_, reaching 35.7 mA cm^–2^ at 0.85 V vs RHE in 0.1 M KOH (Figure 10e,f). Testing with other n-type chalcogenides validated a linear relationship between ORR activity and the work function of the n-type support, highlighting the versatility of this approach for improving the ORR performance of SACs.

Dual atom catalysts (DACs), featuring two different metal atoms isolated at atomic scales within shared support, have recently received increasing attention as an effective strategy to boost catalytic performance by exploiting the synergistic interactions between distinct atomic sites. Regarding ORR, various metal combinations have demonstrated remarkable catalytic activity metrics, including Fe–Mn,^295^ Ni–Co,^296^ Fe–Se,^297^ and Fe–Co.^298,299^ For example, Yasin et al. developed SACs with dual SAs consisting of Fe and Co atoms coordinated with N and S atoms doping carbon nanosheets.^158^ Each element provided a distinct function in ORR. The asymmetric coordination with N and S atoms and the synergistic effect between Fe and Co active sites promoted effective adsorption and activation of O_2_, leading to enhanced reaction kinetics. In fact, this catalyst achieved an *E*_1/2_ of 0.879 V vs RHE and demonstrated long-term cycling stability for Zn–air batteries with minimal deterioration in 0.1 M KOH. Serving as a cathode for a Zn–air battery it allowed for achieving an OCP of 1.52 V, a peak power density of 240 mW cm^–2^, and a discharge-specific capacity of 748 mAh g^–1^ at 10 mA cm^–2^, showing no changes during a 60 h long rechargeability test.

Table 2 reports the key performance metrics of SACs for ORR in 0.1 M KOH and their application in Zn–air batteries. Among the SACs, Fe–N_4_O_1_ and Fe SAs/NC show the highest *E*_1/2_ values (0.924 and 0.93 V, respectively), indicating strong ORR activity, while Fe SAs/NC also exhibits the highest kinetic current density (*j*_k_ = 63.69 mA cm^–2^). All SACs show comparable OCP (∼1.5 V) and discharge specific capacity (750–780 mAh g^–1^) values. However, Fe SAs/NC outperforms the other SACs with a significantly higher peak power density (306.1 mW cm^–2^), emerging as the ideal cathode for Zn–air batteries among those examined in the present review.

**Table 2:** Key Performance Metrics of SACs for ORR in 0.1 M KOH Electrolyte and Zn–Air Batteries

catalyst	*E*_1/2_ [V vs RHE]	Tafel slope [mV dec^–1^]	*j*_k_ [mA cm^–2^]	OCP [V]	peak power density [mW cm^–2^]	discharge-specific capacity [mAh g^–1^]	ref
Fe–N_4_O_1_	0.924	54.79	16.9	1.487	191.5	779.8	^288^
Fe SAs/NC	0.93	77.7	63.69	1.46	306.1	746.1	^289^
Co–N–C	0.89	n/a	17.07	1.5	221	n/a	^292^
Fe–N–C	0.91	58	7	1.53	230	n/a	^293^
FePc/GaS	0.87	n/a	35.7	n/a	n/a	n/a	^294^
Fe/Co–N/S–C	0.879	n/a	n/a	1.52	240	748	^158^

An important study by Xiao et al. addressed key challenges in the development of high-performance, durable, and cost-effective catalysts for proton exchange membrane fuel cells (PEMFCs).^300^ In fact, conventional PEMFCs rely on expensive Pt-based catalysts for ORR, which also suffer from limited durability under long-term operating conditions. To improve the stability, and simultaneously boost the efficiency and decrease the amount of Pt, this study introduces a catalyst composed of atomically dispersed Pt and Fe SAs embedded in N-doped carbon support, complemented by Pt–Fe alloy NPs (Figure 10g). This catalyst achieved a 3.7 times greater mass activity than commercial Pt/C in a fuel cell setup, achieving 1.74 A mg^–1^ at 0.9 V. Moreover, it maintained its high activity over 100,000 voltage cycles, with only a 3% decrease in Pt mass activity, exceeding the U.S. Department of Energy (DOE) target of <40% loss after 30,000 cycles. Furthermore, under a constant potential of 0.6 V, the catalyst sustained a stable current for over 200 h with no significant drop, differently from Pt/C and Fe–N–C catalysts. The authors of the study investigated the performance of the catalyst under H_2_/O_2_ and H_2_/air conditions in a PEMFC, achieving a peak power density of 1.08 W cm^–2^ at a current density of 2 A cm^–2^ (Figure 10h). Despite this value being slightly lower than that of commercial Pt/C (1.37 W cm^–2^), it is notable that in this case, the quantity of Pt in the catalyst was seven times lower than Pt/C. In addition, during an accelerated durability test involving continuous cycling between 0.6 and 0.95 V, it retained 97% of its initial activity over 100,000 cycles, confirming its exceptional stability also within the fuel cell device. Experimental and computational investigations allowed attributing such an outstanding performance to the coexistence of atomically dispersed Pt and Fe sites and Pt–Fe alloy NPs: the former enhances the ORR process through efficient electron transfer pathways, while the Pt-rich shell around a Pt–Fe alloy NPs core promotes stability and enhances ORR kinetics by reducing the energy barriers for intermediate adsorption and desorption (Figure 10i).

In an innovative approach to modifying the electronic state of Fe atoms, Wei et al. introduced Pd NCs into a Fe–N–C system.^301^ This modification caused a transition in the spin state of Fe from a low-spin to an intermediate-spin configuration, which facilitated optimal ORR pathways. As a consequence, the catalyst manifested an *E*_1/2_ of 0.87 V vs RHE, a low Tafel slope of 51.1 mV dec^–1^, a *j*_k_ of 13.82 mA cm^–2^ at 0.85 V vs RHE, and good durability in 0.1 M HClO_4_. As a cathode for fuel cells in a proof of concept demonstration, it delivered an OCP of 0.95 V, a maximum power density of 920 mW cm^–2^, and a current density of 0.61 A cm^–2^ at 0.6 V.

Overall the examples above provide valuable insights to improve the ORR performance of SACs in terms of efficiency and stability for both Zn–air battery and fuel cell applications, serving as the foundations for future studies.

### 5.4 Carbon Dioxide Reduction

The electrochemical reduction of carbon dioxide (CO_2_) into valuable chemicals and fuels is a promising approach to mitigate greenhouse gas emissions and address sustainable energy demand.^302^ However, CO_2_RR is a challenging process, requiring precise control over electron transfer to orient the selectivity and efficiency toward targeted products. Indeed, this reaction can lead to various products, including carbon monoxide (CO), methane (CH_4_), ethylene (C_2_H_4_), and formate (HCO_2_^–^), depending on the catalyst material and reaction conditions. Therefore, to advance this technology it is necessary to develop catalysts capable of driving CO_2_RR efficiently, i.e., at low η, with high FE for specific products. The performance of traditional catalysts, including Cu-based nanomaterials, lacks one or more of these characteristics.^303^ In this context, SACs have emerged thanks to the possibility of engineering the catalysts with atomic precision, increasing not only the catalytic efficiency but also enhancing the selectivity by allowing to properly modify the electronic properties of active sites.^304^ This section provides an in-depth review of recent and relevant studies in the field of CO_2_RR by SACs, presenting a comprehensive understanding of their synthesis, catalytic performance, and mechanism.

CO production is often investigated due to the importance of this product as a feedstock for further chemical processes. The study by Li et al. on P-doped Fe–N–C SAC illustrates how efficiency and selectivity can be optimized for CO production.^305^ The catalyst was synthesized by pyrolyzing a mixture of Fe^3+^-activated carbon black, urea, and triphenylphosphine under an argon atmosphere, resulting in highly dispersed Fe atoms coordinated by N and P within the carbon framework (Figure 11a). It exhibited an outstanding catalytic activity for CO_2_ conversion to CO, obtaining a 98% FE at the low η of 0.34 V with a TOF value of 0.141 s^–1^, which is significantly higher than the 0.058 s^–1^ of the undoped SAC. Durability tests further confirmed the ability of the catalyst to sustain this performance with minimal degradation over time. DFT calculations and XANES analysis demonstrated that P doping reduces the oxidation state of Fe so that more electrons are available for CO_2_RR. Moreover, it promotes the adsorption and activation of CO_2_ molecules, lowers the free-energy barrier for the formation of key intermediates like the *COOH, and facilitates the desorption of CO, favoring CO_2_RR over the competing HER (Figure 11b,c).

**Figure 11:**
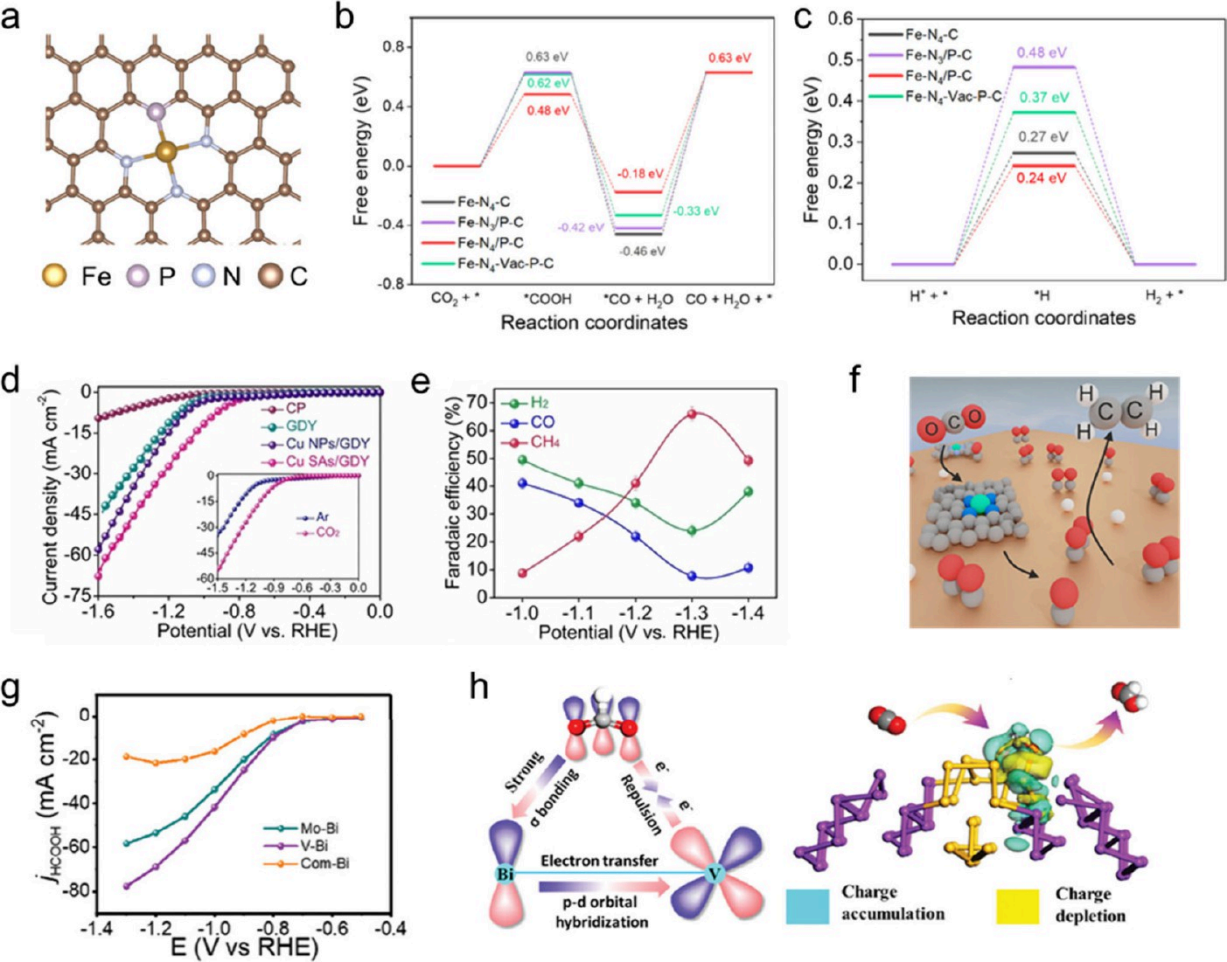
**Carbon dioxide reduction reaction by SACs.** (a) Atomic structure model of the Fe–N/P–C SAC. (b, c) Gibbs free energy profiles of the Fe SAs structures with different coordination environments for (b) electroreduction of CO_2_ to CO and (c) hydrogen adsorption. Reproduced from ref 305. Copyright 2022 American Chemical Society. (d) CO_2_RR to CH_4_ polarization curves of CP, GDY, Cu NPs on GDY (Cu NPs/GDY), and Cu SAs on GDY (Cu SAs/GDY) in Ar- and CO_2_-saturated 0.1 M KHCO_3_. (e) FE of Cu SAs/GDY for H_2_, CO, CH_4_ at various potentials. Reproduced with permission from ref 313. Copyright 2022 Wiley-VCH. (f) Schematic diagram of Ni SAs on the Cu(111) surface, where Ni SAs provide a larger amount of CO and Cu facilitates the C–C bonding. Color scheme: orange, Cu; gray, C; red, O; blue, N; white, H; green, Ni. Reproduced from ref 314. CC BY-NC-ND 4.0. (g) HCOOH partial current density for V–Bi, Mo–Bi, and Com–Bi samples in an H-type cell for CO_2_RR to HCOOH. (h) Schematic representations of the electronic coupling among Bi, V, and *OCHO, and calculated charge density with bonded *OCHO on V–Bi. Reproduced with permission from ref 155. Copyright 2023 Wiley-VCH.

Beyond the experimental optimization of SACs, computational approaches play a crucial role in accelerating catalyst design by identifying key activity and selectivity descriptors. The study of Mou et al. combined intuition-driven dimensionality reduction strategies with ML methods to identify key descriptors based on atomic properties and the geometric configuration of SACs.^306^ These descriptors enabled the creation of accurate ML models to predict limiting potentials for the production of formic acid (HCOOH), CO, and CH_4_/methanol (CH_3_OH) on 48 different SACs@UiO-66-X (X = H, NH_2_, and Br). The transferability of these models was further validated on an additional 48 systems with different functional groups (X = CH_3_, OH, and NO_2_). The predicted activity and selectivity trends were confirmed through additional DFT calculations. In the study of Huang et al., the role of heteroatom doping in M-N_4_–C SACs for CO_2_ electroreduction was investigated.^307^ By integrating DFT calculations with ML techniques, the authors systematically evaluated the influence of dopants on the catalytic activity and selectivity of SACs. The study highlights that heteroatom doping can modulate the electronic structure, optimize adsorption energies, and enhance reaction pathways, leading to improved CO_2_ reduction performance.

Building on the foundations laid by SACs, DACs offer new ways to improve the efficiency of the CO_2_RR, as demonstrated by Wang et al. thanks to their innovative Fe_2_–N–C DAC.^308^ Despite Fe–N–C SACs having shown low η values for CO production from CO_2_, their performance is limited by the slow product desorption due to the strong binding of *CO intermediates on Fe sites. DACs can overcome these limitations by combining the unique properties of SAC with the synergistic effect due to two close metal atoms. Indeed, the Fe_2_–N–C DAC, obtained via the pyrolysis of Fe-loaded ZIF-8 MOF, exhibited an 80% FE over the potential range of −0.5 to −0.9 V vs RHE, and a high TOF of 7.40 s^–1^ at 1.0 V, which is almost three times higher than Fe–N–C SAC. The dual-atom system improved also the stability of the catalyst, with a 95.85% retention of the FE after continuous operation for 20 h. Experimental and theoretical analyses indicated that the synergy between the two Fe atoms decreases the energy gap between bonding and antibonding states in *CO adsorption, boosting CO_2_ conversion into CO compared to the SA configuration.

SACs demonstrate great potential not only in enhancing the electrocatalytic activity toward CO_2_RR but also in modulating the reaction pathways to achieve high selectivity. One effective strategy to regulate CO_2_RR products is to properly tailor the atomic coordination environment of SACs. In this regard, a postsynthetic metal substitution (PSMS) strategy has been reported to produce Ni–N_*x*_–C SACs with varied nitrogen coordination numbers (denoted as N_*x*_) using N-doped carbon derived from a MOF as support.^309^ Among them, the Ni–N_3_–C SAC exhibited exceptional selectivity for CO, reaching the highest FE of 95.6% at −0.65 V vs RHE, while Ni–N_4_–C SAC showed an FE of 89.2%. Theoretical calculations explain this improvement based on the enhanced formation of the *COOH intermediate in correspondence with the reduced Ni coordination number in Ni–N_3_–C, which accelerates CO_2_ reduction to CO.

Another study demonstrated how dynamic coordination environments in SACs can be exploited to control selectivity.^310^ Particularly, Ga SACs were designed on ZIF-8-derived N-doped carbon support to harness the unique fluxional properties of Ga, a liquid metal element capable of adaptive deformation during catalysis. Unlike traditional rigid active site models, Ga SACs featured dynamic structural transitions that optimize the adsorption energies of the *COOH intermediate and, consequently, promote CO production with an FE up to ∼92% at −0.3 V vs RHE. Moreover, the regeneration of the active sites during CO_2_RR improved the stability of the SAC.

Additionally, an asymmetric coordination strategy was employed to prepare a Fe SAC with one S and three N atoms (Fe–S_1_N_3_–C), resulting in a higher selectivity toward CO.^311^ This structure, characterized by a significant geometric distortion due to the large radius of S, enabled the self-relaxation of the Fe–S_1_N_3_ site, allowing independent regulation of the adsorption energies for the *COOH and *CO intermediates, as indicated by operando XAFs and DFT calculations. As a consequence, the Fe–S_1_N_3_–C achieved an impressive CO Faradaic efficiency of 99.02% at −0.50 V vs RHE and high stability.

Another important product of the CO_2_RR is CH_4_. It is a high-energy-density fuel and one of the primary components of natural gas. The conversion of CO_2_ to CH_4_ provides a means to store renewable energy (e.g., from solar or wind) as chemical energy, which can be burned or used directly as a fuel in existing infrastructures. In addition, it is a valuable feedstock in the chemical industry. CO_2_ reduction to CH_4_ is not easy to achieve, as it involves a kinetically challenging eight-electron reduction process compared to CO production. However, SACs have demonstrated the ability to overcome the unfavorable energy barriers associated with this reaction. Aiming at improving the efficiency of CH_4_ production from CO_2_, Chen et al. prepared a novel SAC based on Cu SAs ligated with N-heterocyclic carbene (NHC) and encapsulated within the UiO-67 MOF.^312^ This SAC displayed a selectivity to CH_4_ over the potential range −1.1 to −1.6 V vs RHE, reaching a FE of 81% at −1.5 V vs RHE with a high partial current density of −340.2 mA cm^–2^, surpassing the best previous catalysts. Furthermore, the TOF was calculated as 16.3 s^–1^, establishing a new benchmark for Cu-based catalysts in CH_4_ production, and a durability test demonstrated the stability of the catalyst with minimal loss in efficiency. Mechanistic studies revealed that the NHC ligand’s strong σ-donating effect increases the electron density at the Cu sites, facilitating the preferential adsorption of *CHO intermediates, vital for forming CH_4_. At the same time, MOF support’s porosity ensures efficient CO_2_ diffusion to the Cu atoms, maximizing the availability of active sites and enhancing the catalytic efficiency.

Different strategies for CH_4_ selectivity reveal the versatility of SAC designs, where support materials play a critical role. In this regard, Shi et al. developed an SAC by anchoring Cu SAs to graphdiyne (GDY).^313^ Although confirming the superior electrocatalysis by SAs over NPs (Figure 11d), the CH_4_ production metrics of this catalyst are slightly worse than the previous example, reaching a maximum FE of 66% at −1.3 V vs RHE, a partial current density of −24 mA cm^–2^, and a TOF of 0.64 s^–1^. However, the key feature of this SAC is the robust Cu–C bonds, which alter reaction intermediates adsorption favoring CH_4_ over CO production (Figure 11e), by promoting the *OCHO pathway over the *COOH pathway, as indicated by in situ spectroelectrochemical analysis and DFT calculations.

A partial-carbonization strategy was developed to modify the electronic structure of SACs, leading to the synthesis of CuN_2_O_2_ active sites embedded in carbon dots.^95^ These catalysts achieved remarkable selectivity, with 99% of CO_2_ reduction products being CH_4_ and a high FE of 78% at a current density of 40 mA·cm^–2^. DFT calculations revealed that the introduction of oxygen ligands fine-tunes the electronic structure of Cu active sites, suppressing HER and favoring CH_4_ production.

Additionally, Cu SAs supported on 2D carbon nitride frameworks, such as polyheptazine imide (PHI) and polytriazine imide (PTI), via a simple metal-ion exchange reaction demonstrated outstanding selectivity toward CH_4_ formation.^65^ The Cu–N_2_ coordination environment at the vertices of the triangular carbon nitride nanopores enabled achieving an FE of ∼68% and a partial current density of 348 mA·cm^–2^ at −0.84 V vs RHE. These results originated from carbon nitride frameworks allowing precise control over Cu–Cu distances in the nanopores and the cooperative catalytic effect between Cu and carbon nitride, enhancing intermediate stabilization and accelerating rate-limiting steps for CH_4_ production.

As the complexity of CO_2_RR products increases, new challenges emerge. Although being considered the optimal catalyst for C–C coupling, Cu-based catalysts show low efficiency for CO_2_ conversion to C_2_H_4_ due to limitations in the CO_2_ to CO conversion step, resulting in poor surface coverage by CO and low current densities for C_2_H_4_ production. Nevertheless, tandem catalysts provide a strategic solution for facilitating this issue, as exemplified by the work of Liu et al., who dispersed Ni SAs onto a Cu catalyst by an electrostatic self-assembly method.^314^ This design was chosen based on Ni broad CO generation-potential window, allowing efficient CO supply to the Cu surface at the exact potential range required for C–C coupling. The SAC allowed boosting C_2_H_4_ production compared to the bare Cu catalyst (Figure 11f), achieving a partial current density of 370 mA cm^–2^ with a 62% FE at approximately −1.3 V vs RHE, remaining stable over 14 h operation at a current density of 500 mA cm^–2^ in a flow-cell reactor.

The production of HCO_2_^–^ represents a distinct CO_2_RR pathway, offering valuable applications as an intermediate in chemical synthesis. For this reason, Cao et al. developed an innovative SAA catalyst via the pyrolisis of Bi_*x*_MO_*y*_ nanosheets (M = V, Mo, W) synthesized by a hydrothermal method, followed by the incorporation of V or Mo SAs via electrochemical reduction.^155^ Among these, the V–Bi SAA showed exceptional catalytic performance, showing an FE of 96.1% at −1.0 V vs RHE, above 90% selectivity within the broad potential range of −0.8 to −1.3 V vs RHE, a HCOOH partial current density of 77.7 mA cm^–2^ at −1.3 V vs RHE (Figure 11g), and stable activity over 90 h operation. Insights obtained via in situ spectroscopy analyses and DFT computations suggest that the strong p–d orbital hybridization between M and Bi enhances electron delocalization and CO_2_ activation at Bi sites, lowering the protonation energy barrier for HCO_2_^−^ production (Figure 11h). Extending its applicability, the researchers integrated the V–Bi SAA in a solar-driven electrochemical cell, reaching an impressive 90.5% yield of 2,5-furandicarboxylic acid production from 5-hydroxymethylfurfural, a versatile biomass derivative, revealing the potential of this technology also in the biomass valorization.

Unlike conventional Cu catalysts, which often suffer from low selectivity due to the competing *COOH and *HCOO pathways, a study introduced a Pb-alloyed Cu SAC (Pb_1_Cu) that uniquely modulates the electronic and geometric properties of Cu active sites, favoring the *HCOO pathway and, therefore, steering the reaction exclusively toward HCO_2_^–^ production, attaining a 96% FE at −0.8 V vs RHE.^315^ This outstanding selectivity is attributed to the isolated Pb atoms, acting as electronic modifiers rather than direct active sites, which increase the energy barrier for *COOH formation and simultaneously suppress HER. Overall the catalyst displayed an excellent performance with a high current density exceeding 1 A cm^–2^ and stability for over 180 h in a solid electrolyte reactor, continuously producing pure HCOOH.

### 5.5 Ammonia Production

NH_3_ ranks as the second most-produced chemical globally, valued for its long-standing use as a major agriculture fertilizer as well as a potential energy carrier, considering its low liquefying pressure and high hydrogen density. However, traditional NH_3_ synthesis through the Haber–Bosch process is energy-intensive and generates substantial CO_2_ emissions, triggering serious environmental and sustainability concerns.^316,317^ In response, the scientific community is intensively exploring electrochemical methods for NH_3_ synthesis, which promise reduced energy demands and a significant reduction in CO_2_ emissions. The electrocatalytic process offers a green and sustainable pathway to NH_3_ production, employing diverse nitrogen sources, including molecular nitrogen (N_2_), NO_3_^–^, nitrite (NO_2_^–^), and nitric oxide (NO). Each nitrogen source exhibits distinct properties that influence its suitability and efficiency for electrochemical NH_3_ synthesis. For example, N_2_, though abundant, presents significant challenges for conversion due to its robust triple bond (942 kJ mol^–1^), which necessitates substantial energy input for dissociation. In contrast, N–O compounds possess weaker bonds, facilitating easier reduction and thus offering more energy-efficient pathways for NH_3_ synthesis. Additionally, the environmental concerns associated with NO and NO_2_ as pollutants make these sources attractive for sustainable NH_3_ synthesis.^318,319^ The conversion to NH_3_ and the corresponding numbers of electrons involved are illustrated in eqs 1–4:

1 $$N_{2}+6H^{+}+6e^{-}\to 2{\mathrm{NH}}_{3}$$

2 $${{\mathrm{NO}}_{3}}^{-}+9H^{+}+8e^{-}\to {\mathrm{NH}}_{3}+3H_{2}$$

3 $${{\mathrm{NO}}_{2}}^{-}+7H^{+}+6e^{-}\to {\mathrm{NH}}_{3}+2H_{2}O$$

4 $$\mathrm{NO}+5H^{+}+5e^{-}\to {\mathrm{NH}}_{3}+H_{2}O$$

The performance of electrocatalysts in NH_3_ synthesis is assessed through several critical parameters, including NH_3_ yield and FE, selectivity, and stability. Furthermore, as an electrochemical process, optimizing the reduction potential is crucial to improving energy efficiency. These characteristics rely on the number of active sites and their intrinsic activity. SACs have demonstrated considerable promise in electrochemical NH_3_ synthesis as they maximize active site utilization and significantly enhance catalytic efficiency. However, this field is still evolving, with significant opportunities for further advancement, particularly in enhancing catalyst stability and improving the yield.

#### 5.5.1 Electrochemical Nitrogen Reduction to Ammonia

The electrochemical N_2_ reduction reaction (NRR) is acquiring significant attention as a highly promising method that can be conducted at atmospheric pressure and moderate temperatures using renewable electricity sources. Since NRR occurs at a similar potential to HER from a thermodynamic point of view, the competition between these reactions poses a significant challenge in achieving high selectivity for NRR.

Fe is well-known for its catalytic properties in NRR, serving as the catalyst in the industrial Haber-Bosch process and playing a crucial role in biological nitrogen fixation. In this regard, Wang et al. synthesized Fe SAs anchored on N-doped carbon (Fe SAs/N–C) by optimized modulation of the polypyrrole–Fe coordination complex, demonstrating the possibility of highly efficient and selective electrochemical NRR under ambient conditions by positively shifting the reaction potential.^320^ They obtained an exceptional NH_3_ yield of 7.48 μg h^–1^ mg^–1^ and an FE of 56% at 0 V vs RHE. This remarkable performance results from the combined effects of HER suppression and NRR enhancement.

In one of the major studies, Li et al. anchored Fe SAs to a Pd metallene (PdFe_1_) through a one-step wet chemistry strategy for NRR.^321^ The unique structure of Pd metallene makes it an ideal platform for hosting SAs. They have identified a Pd-coordinated Fe SA as the active center for efficiently activating N_2_, and conducted an isotopic labeling experiment using ^1^H NMR with ^15^N_2_ as the input gas to show that all the NH_3_ produced is solely from the reduction of the precursor N_2_ gas (Figure 12a). Moreover, they evaluated its performance using chronoamperometry measurements followed by the quantification using UV–vis spectrophotometry (Figure 12b), achieving an NH_3_ yield of 111.09 μg h^–1^ mg^–1^ and an FE of 37.8% at −2 V vs RHE (Figure 12c), surpassing nearly all previously reported Pd-based NRR catalysts. Together with the higher performance they also reported great catalytic stability with 100 h electrolysis and no significant decline in NH_3_ yields or Faradaic efficiencies observed across ten cycling tests (Figure 12d).

**Figure 12:**
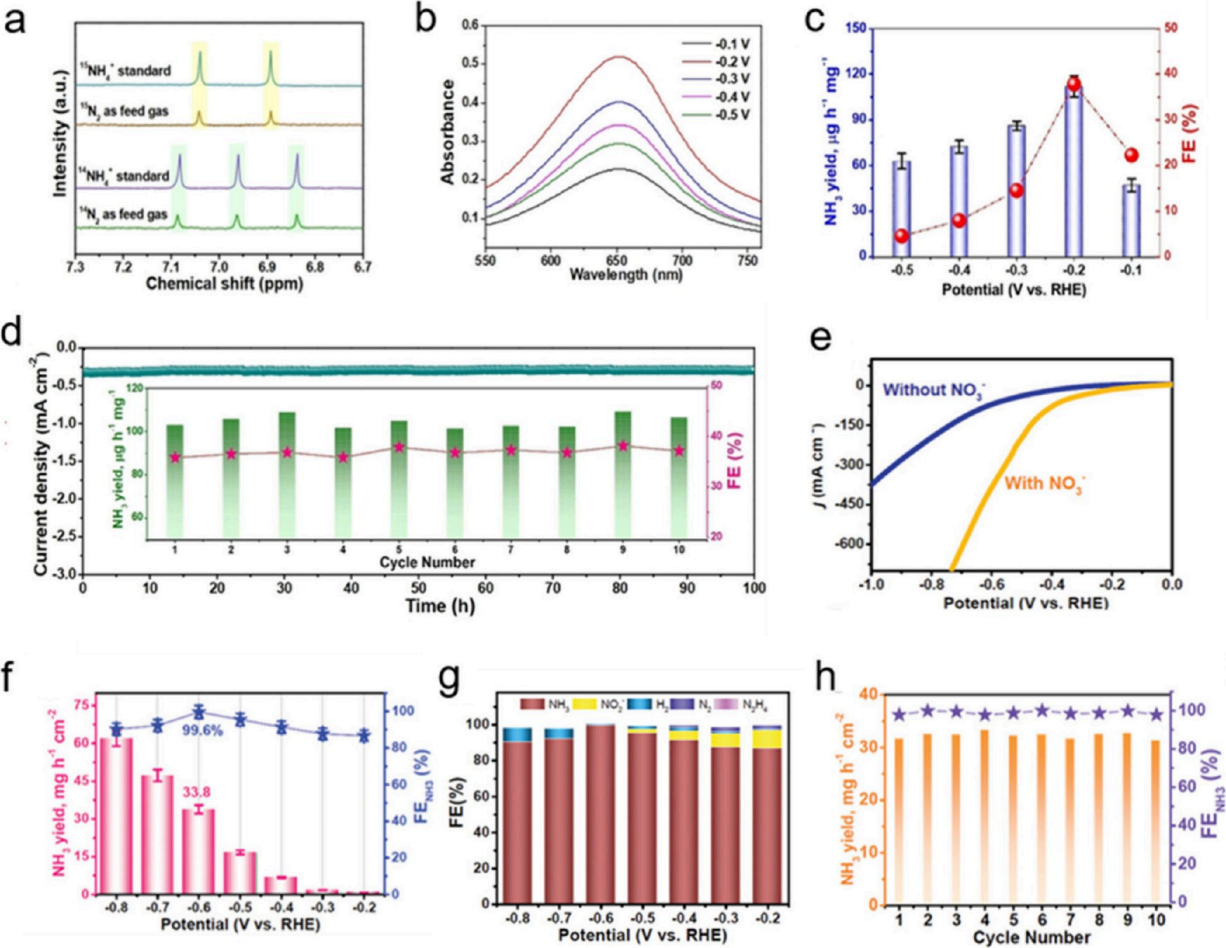
**Ammonia production by SACs.** (a) ^1^H NMR measurements fed by ^14^N_2_ and ^15^N_2_ gases and ^14^NH_4_^+^/^15^NH_4_^+^ standard samples, confirming that NRR by PdFe_1_ SAC produces NH_3_. (b) UV–vis absorption spectra and (c) calculated NH_3_ FEs and yield. (d) Long-term chronoamperometry test at −0.2 V vs RHE for 100 h and cycling test (inset). Reproduced with permission from ref 321. Copyright 2022 Wiley-VCH. (e) Polarization curves of the Bi_1_Pd SAC in 1 M KOH with and without 0.1 M NO_3_^–^. (f) NH_3_ yield and FE at various potentials. (g) FEs of different products (NO_2_^–^, N_2_H_4_, H_2_, and N_2_) after NO_3_RR electrolysis at various potentials. (h) NH_3_ yield and FE following cyclic chronoamperometry tests at −0.6 V vs RHE. Reproduced with permission from ref 325. Copyright 2023 Wiley-VCH.

The coordination of metal SAs, their electronic structure, and the pH of the electrolyte used during electrolysis are all crucial factors that influence NH_3_ yield and FE. The coordination environment plays a key role in optimizing the performance of a SAC by tuning its electronic structure. Typically, metal SAs are coordinated by N with varying coordination numbers. However, O coordination offers the potential for improved H_2_ activation without hindering N_2_ activation, which could result in increased NH_3_ production. Zhang et al. pioneered the synthesis of Fe SAs coordinated with O, reporting a high NH_3_ yield.^196^ They developed Fe-based SACs supported on N-free lignocellulose-derived carbon, using both carbon cloth and glassy carbon substrates (Fe-SAs/LCC/CC and Fe-SAs/LCC/GC, respectively). Notably, Fe-SAs/LCC/GC exhibited an exceptionally high NH_3_ yield of 307.7 μg h^–1^ mg^–1^ and a higher FE of 54.1% at −0.05 V vs RHE compared to the Fe-SAs/LCC/CC. The Fe–(O–C_2_)_4_ coordination was revealed as the active site for NRR, where the adsorption of N_2_ occurred through a back-donation mechanism. The superior catalytic activity of Fe-SAs/LCC/GC was confirmed by electrochemical impedance spectroscopy (EIS) analysis, which revealed a higher charge transfer resistance of Fe-SAs/LCC/CC (3.58 Ω) compared to Fe-SAs/LCC/GC (0.013 Ω) and the thin catalyst layer on the GC substrate.

The study by Zafari et al. explored the potential of 2D transition metal borides (MBenes), defective 2D materials, and 2D π-conjugated polymers as efficient catalysts for NRR.^322^ DFT calculations reveal that nitrogen molecules can be effectively trapped in the cavities of MBenes, leading to enhanced adsorption strength and bond elongation of N≡N. Furthermore, defective 2D materials with vacancies of Te, Se, and S create unique environments that facilitate N_2_ activation in the vicinity of transition metal centers, significantly improving catalytic performance. The study introduced a novel NRR mechanism that combines dissociative and associative pathways and develops an ML-based screening strategy for predicting efficient NRR electrocatalysts. Promising materials such as TaB, NbTe_2_, NbB, HfTe_2_, MoB, MnB, HfSe_2_, TaSe_2_, and Nb@SAC exhibit remarkable selectivity against HER with overpotentials ranging from 0.17 to 0.64 V. The study of Zhang et al. investigated an innovative approach to catalyst design by employing interpretable ML models trained on non-DFT-calculated features.^323^ The authors successfully predicted reaction Gibbs free energy for SACs supported on graphene, demonstrating the high accuracy of the gradient boosting regression (GBR) model with *R* ^2^ values of 0.972 and 0.984 for key reaction steps. Feature importance analysis reveals that covalent radius, d-electron occupation, and coordination type play critical roles in catalytic performance.

#### 5.5.2 Electrochemical Nitrate Conversion to Ammonia

The electrochemical reduction reaction of NO_3_^–^ to NH_3_ (NO_3_RR) is garnering significant interest among researchers, primarily due to the lower bond dissociation energy of the N=O bond in comparison to the much stronger N≡N triple bond in N_2_. This approach addresses critical environmental concerns, particularly the issue of NO_3_^–^ contamination in groundwater, which poses risks to ecosystems and human health. Additionally, the economic burden associated with NO_3_^–^ remediation efforts is substantial. NO_3_RR involves a complex transfer of eight electrons, presenting both a challenge and an opportunity for catalyst development in achieving efficient and selective NH_3_ production.

In this context, a study by Zhang et al. showed that the local structural environment of an SAC can be tailored through the synergistic interaction between hetero SAs and surface defects, enabling precise manipulation of the catalytic performance.^161^ In particular, they reported an Au SAs supported on 2D Cu nanosheets (Au_1_Cu­(111)) and further constructed Cu vacancies on their surface (V_Cu_-Au_1_Cu SAAs) via facile galvanic replacement with a subsequent dealloying process. This SAC was employed for NO_3_RR with an NH_3_ yield of 555 μg h^–1^ cm^–2^ and an FE of 98.7% at −2 V vs RHE, recovering 97% of the produced NH_3_ by a simple distillation method. The large surface area and the high selectivity for NO_3_RR make the Cu nanosheet an ideal host material. In addition, the electron migration from Cu to Au atoms creates electron-deficient Cu active sites, which promote the generation of active hydrogen species (*H) that can readily hydrogenate NO_3_^–^.

Liu et al. developed an efficient SAC for NO_3_RR consisting of Rh clusters and SAs dispersed onto Cu nanowires (NWs) by galvanic replacement reaction between Cu and Rh^3+^, delivering a partial current density of 162 mA cm^–2^ for NH_3_ production with a record NH_3_ yield of 1.27 mmol h^–1^ cm^–2^ and an FE of 93% at 0.2 V vs RHE.^324^ Comprehensive investigations using EPR, in situ infrared spectroscopy, differential electrochemical mass spectrometry (DEMS), and DFT modeling revealed that the exceptional activity stems from the synergistic catalytic interplay between Rh and Cu sites. In this mechanism, H^+^ adsorbed on the Rh site is transferred to nearby *NO intermediate species adsorbed on the Cu site, facilitating hydrogenation and promoting NH_3_ formation.

In another interesting study, Chen et al. reported a highly active, selective, and stable SA Bi alloyed Pd metallene (Bi_1_Pd) for NO_3_RR.^325^ The NO_3_RR performance of the SAC can be appreciated from the polarization curves in Figure 12e. The Bi_1_Pd exhibited an enhanced current density beyond an onset potential of −0.2 V when NO_3_^–^ is present, indicating its high catalytic activity for NO_3_RR. As shown in Figure 12f, combined chronoamperometric (1 h electrolysis) and colorimetric tests were conducted to quantify the potential-dependent NO_3_RR performance of Bi_1_Pd, revealing that the NH_3_ FE reaches nearly 100% at −0.6 V vs RHE. At this potential, the corresponding NH_3_ yield and partial current density are 33.8 mg h^–1^ cm^–2^ and 425.3 mA cm^–2^, respectively. Controlled colorimetric and NMR measurements confirmed that the NH_3_ produced is derived from the Bi_1_Pd-catalyzed NO_3_RR process. This performance is attributed to Bi SAs, which electronically couple with neighboring Pd atoms to synergistically activate NO_3_^–^ and destabilize *NO, leading to the reduced energy barrier of the rate-determining step and enhancing the protonation energetics of NO_3_^–^ to NH_3_ pathway. They also evaluated all the possible byproducts of the reaction, including NO_2_^–^, hydrazine (N_2_H_4_), H_2_, and N_2_ to study the selectivity of the Bi_1_Pd catalyst for NO_3_RR, finding that NO_2_^–^ is the primary byproduct within the potential range −0.2 to −0.4 V vs RHE, but nearly vanishes when the potential exceeds −0.5 V vs RHE before H_2_ production due to HER becoming significant beyond −0.6 V vs RHE (Figure 12g). In addition to its outstanding NO_3_RR activity and selectivity, Bi_1_Pd demonstrated exceptional stability and cyclability based on a 20 h chronopotentiometry test with minimal variation in current density and FE and minor fluctuations in FE and NH_3_ yield following ten consecutive cycles (Figure 12h). Control experiments with Pd metallene showed a much inferior performance, highlighting the significant role of Bi_1_ incorporation in substantially enhancing the NO_3_RR activity.

Among the non-noble metal SACs for NO_3_RR, Cu ones are among the best. An interesting study was carried out by Yang et al. by synthesizing a Cu–N_4_ SAC, using operando XAS to provide strong evidence that the as-synthesized structure underwent a successive transformation to near-free Cu^0^ SAs and eventually aggregated Cu^0^ NPs as the applied potential shifted from 0 to −1 V vs RHE.^159^ This structural evolution was accompanied by a significant enhancement in the NH_3_ production, indicating that the reaction-induced Cu^0^ NPs, rather than the original Cu–N_4_ structure, serve as the real active sites. The catalyst showed an NH_3_ yield of 4.5 mg h^–1^ cm^–2^ and an FE of 84.7% at −1 V vs RHE. Furthermore, after electrolysis, the aggregated Cu^0^ NPs can revert to SAs and restore the Cu–N_4_ structure when exposed to ambient conditions.

Fe is also recognized for its effectiveness in NO_3_RR. As the industrial catalyst for the Haber–Bosch process for decades, the potential SA Fe efficiency warrants thorough investigation. For this reason, Wu et al. reported a highly selective and active Fe SAC using a transition metal-assisted carbonization method with SiO_2_ powders as a hard template.^326^ The catalyst displayed an NH_3_ yield of ∼20,000 μg h^–1^ mg^–1^ and an FE of ∼75% by successfully preventing N–N coupling and promoting NH_3_ selectivity. Despite the lower Fe content, the Fe SAC showed a significantly improved NO_3_RR performance compared to the Fe NPs counterpart.

Conventionally, the NO_3_RR is performed at a constant applied potential. However, Li et al. introduced a Cu single-atom gel (Cu SAG) that produces NH_3_ from NO_3_^–^ and NO_2_^–^ in neutral conditions under potential pulses.^327^ SAGs have recently gained attention as a promising class of electrocatalysts, combining the structural features of SA active sites with 3D channel frameworks. At the same time, the pulse electrolysis strategy enables sequential accumulation and conversion of NO_2_^–^ intermediates during NO_3_^–^ reduction. Indeed, this method effectively minimized the competition from HER, leading to substantial enhancements in NH_3_ yield and FE compared to traditional constant potential electrolysis. The energy consumption analysis indicated that pulse electrosynthesis of NH_3_ could be highly practical, achieving approximately 22% power savings and a ∼28% increase in yield.

The work by Lu et al. demonstrated the large-scale capabilities of computational methods combined with ML.^328^ The authors predicted the catalytic performance of 1891 previously unexplored SACs for the NO_3_RR. By employing a four-step screening strategy, assessing stability, NO_3_^–^ adsorption, activity, and selectivity, they identified 10 promising SACs that outperform benchmark materials. The study not only presents novel candidates and descriptors for NO_3_RR but also establishes an efficient, rapid, and cost-effective methodology for discovering and designing valuable SACs.

#### 5.5.3 Electrochemical Nitrite Reduction to Ammonia

NO_2_^–^ is also a promising nitrogen source for electrochemical NH_3_ synthesis, primarily due to the relatively low dissociation energy of the N=O bond. Similar to NO_3_^–^, NO_2_^–^ arises as an environmental pollutant, commonly originating from fertilizers and various industrial activities. However, NO_2_^–^ is particularly advantageous as it offers higher selectivity and requires lower energy input for reduction, implying improved energy efficiency. Moreover, the NO_2_^–^ reduction reaction to NH_3_ (NO_2_RR) produces minimal byproducts, further enhancing its appeal. Nevertheless, the inherently lower stability of NO_2_^–^ compared to NO_3_^–^ poses a challenge for process efficiency and control, necessitating advanced catalyst designs and reaction conditions to optimize the performance.

Xiang et al. developed an efficient SAC by obtaining Rh SAs on BN nanosheets via a straightforward two-step synthesis method and used it for NO_2_RR.^329^ Their experimental results demonstrated that the catalyst, when integrated into a flow cell, achieves an NH_3_ yield of 2165.4 μmol h^–1^ cm^–2^ and an outstanding FE of 97.83% at a current density of 355.7 mA cm^–2^. With support from DFT studies, the authors proposed that the Rh sites, along with promoting the activation and hydrogenation of the NO_2_^–^ to NH_3_ process, also diminish the undesired HER.

In a further study, Wan et al. designed highly active and durable Zn SAs supported on MnO_2_ NWs through combined hydrothermal and impregnation methods.^330^ Thanks to atomic scale characterizations, they showed that Zn SAs coordinated with three surface O atoms of MnO_2_ to form Zn_1_–O_3_ units. These enhance NO_2_^–^ activation and the stabilization of key intermediate *NHO and reduce the energy barrier of the NO_2_^–^ to NH_3_ hydrogenation process. Based on these properties, this SAC reached an NH_3_ FE of 95.3% at the current density of −288.5 mA cm^–2^.

Instead, Du et al. prepared Nb SAs on a ZrO_2_ substrate, leading to a dual-site Nb_1_–Zr configuration that overall boosted the NO_2_RR by promoting the adsorption and activation of NO_2_^–^ and key intermediates, thus lowering the energy barrier of the process.^331^ Particularly, they achieved an NH_3_ yield of 1588 μmol h^–1^ cm^–2^ and a maximum FE of 95.87% at a current density of 263.59 mA cm^–2^ in a flow cell, along with stability up to 100 h, which is among the best of reported NO_2_RR electrocatalysts.

#### 5.5.4 Electrochemical Nitric Oxide Reduction to Ammonia

The reduction of NO presents a promising dual-purpose strategy, addressing the removal of harmful NO emissions while simultaneously enabling the efficient synthesis of NH_3_. As a common pollutant resulting from industrial processes and vehicle exhaust, NO contributes significantly to environmental and health issues, including acid rain formation and issues for the human respiratory system. NO reduction reaction (NORR) offers a pathway to mitigate these impacts by converting NO into valuable NH_3_ under controlled conditions. This approach is particularly advantageous due to the lower bond dissociation energy of NO compared to N_2_. NORR is a multielectron transfer process, often involving intermediates such as nitroxyl (HNO) or hydroxylamine (NH_2_OH), and requires precise control to achieve NH_3_ production, avoiding the competitive HER. The development of efficient, selective catalysts for NORR is crucial.

In this framework, Chen et al. conducted a pioneering study on NORR by SACs using main group p-block elements, which are excellent candidates for NORR but lack satisfactory exploration. In particular, they employed a one-step supercritical CO_2_ approach to synthesize Sb SAs confined in amorphous MoO_3_ (Sb_1_/a-MoO_3_), which exhibited an NH_3_ yield of 273.5 μmol h^–1^ cm^–2^ and an FE of 91.7% at −0.6 V vs RHE.^332^ Theoretical analysis confirmed that the isolated Sb sites are responsible for the outstanding performance of the catalyst, optimizing the adsorption of *NO/*NHO intermediates, decreasing the energy barriers associated with the reaction, and, simultaneously, exhibiting higher affinity toward NO rather than H_2_O/H species.

By integrating high-throughput DFT calculations with ML techniques, Zhao et al. designed a series of SACs and evaluated their NO reduction capabilities.^333^ Notably, SACs such as MoS_2__N_3__Zn, MoS_2__N_3__Cd, and Mo_N_3__Zr demonstrated exceptional performance, attributed to a self-regulating coordination environment facilitated by the support and N atoms. The study also introduced linear and volcanic descriptors to quantify catalyst performance, emphasizing the effectiveness of gradient boosting regression (GBR) in predicting and classifying SACs efficiency. A DFT-ML study by Yang et al. explored SACs based on wavy antimony nitride (SbN), investigating 23 different transition metals as active centers.^334^ Nb@SbN emerged as the most promising candidate, achieving nearly 100% Faraday efficiency and setting a record-low limiting potential of −0.06 V.

### 5.6 Other Electrocatalytic Processes

SACs demonstrated remarkable efficiency in water splitting, ORR, CO_2_RR, and NH_3_ production. However, their utility extends far beyond these applications. This section presents the potential of SACs in catalyzing other important electrocatalytic processes.

For example, Tian et al. presented a study on the selective photoelectrochemical (PEC) oxidation of glucose to glucaric acid (GLA) using an SAC (Figure 13a).^339^ The interest behind this electrocatalytic process lies in the significant economic value and high demand for GLA, which is recognized as a top value-added chemical being used in many products, including biodegradable polymers, personal care products, and pharmaceuticals, and as a preserver in food and beverages. Moreover, it is produced from the oxidation of glucose derived from biomass, which promotes a circular economy. Traditional methods for GLA production, such as microbial fermentation and chemical oxidation, often require harsh conditions and lack selectivity, necessitating expensive processes to isolate GLA from the various byproducts, among which is gluconic acid (GLU). On these bases, the PEC approach represents a green alternative, utilizing renewable energy such as sunlight and operating under mild conditions without hazardous chemical oxidants. For this purpose, a TiO_2_ nanorod array grown by a hydrothermal method was defected by electrochemical reduction, introducing oxygen vacancies that enhance charge separation and reduce the band gap of the TiO_2_, extending the absorption of the semiconductor to the visible light. Pt SAs were then deposited on the defective TiO_2_ using ALD. Under simulated sunlight irradiation, the defective TiO_2_ and the SAC achieved similar photocurrent densities of 1.91 and 1.86 mA cm^–2^ for glucose oxidation at 0.6 V vs RHE. Nevertheless, after 5.5 h, the SAC converted 98.8% of the glucose to GLU and, then, to GLA, with yields of 9.2% and 84.3%, respectively. In contrast, the defective TiO_2_ exhibited a glucose conversion efficiency of 97.2% with GLU and GLA yields of 77.7% and 13.8%. Control experiments using Pt NPs or an increased number of ALD cycles proved to be detrimental to the performance of the catalyst, as expected from a SAC undergoing agglomeration of the active sites. More in-depth analyses revealed that this selectivity derives from the accelerated oxidation of GLU to GLA at the SA Pt sites, as suggested by the measured rate constants (Figure 13b).

**Figure 13:**
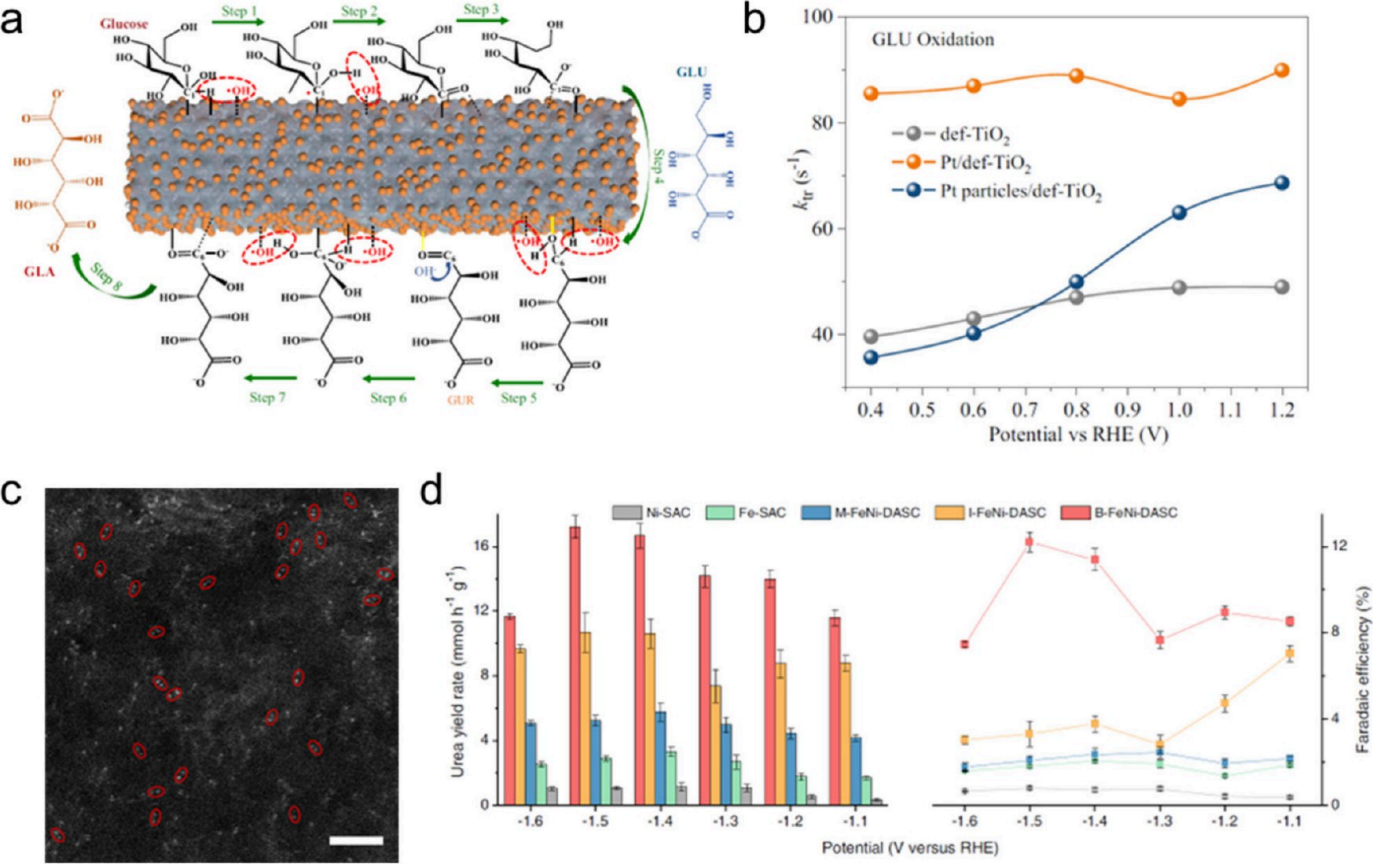
**Other electrocatalytic reactions by SACs.** (a) Schematic illustration of the photoelectrochemical conversion mechanism of glucose to GLA by Pt SAs on defective TiO_2_ nanowire array (Pt/def-TiO_2_). (b) Rate constant for GLU oxidation to GLA at different potentials. Reproduced from ref 339. CC BY 4.0. (c) HAADF-STEM image of FeNi-DASC showing the dual atomic sites. The scale bar is 2 nm. (d) Urea yield rate and FE at different potentials of Ni SAC, Fe SAC, and Fe–Ni diatomic electrocatalyst (FeNi-DASC) prepared with different approaches. M-FeNi-DASC was obtained by mixing Ni and Fe SACs. Reproduced from ref 277. CC BY 4.0.

In another study related to biomass valorization using SAC, Ge et al. investigated the selective electrooxidation of biomass-derived alcohols.^335^ This is a promising approach for the sustainable production of industrially important chemicals, such as aldehydes. Conventionally, this process is performed using non-noble metal catalysts that operate in alkaline electrolytes. Nonetheless, in these media, side reactions like dimerization or further oxidation of aldehydes to carboxylates occur, dramatically reducing the aldehydes formation yield. For these reasons, performing the alcohol oxidation reaction in neutral media is highly desirable. To achieve this result, Ru SAs were deposited on NiO nanosheets by wet impregnation and calcination. This SAC induced the electrooxidation of 5-hydroxymethylfurfural (HMF) to 2,5-diformylfuran (DFF), an important intermediate for the pharmaceutical and chemical industry, reaching a current density of 10 mA cm^–2^ at the low potential of 1.283 V vs RHE in a neutral electrolyte (1 M PBS). Furthermore, it showed an impressive selectivity of 90% and an FE of 70% for DFF production at 1.5 V vs RHE. An extensive investigation of the reaction mechanism revealed the crucial role of the SA Ru sites in promoting water dissociation to generate more *OH species compared to bare NiO nanosheets in the neutral medium, which successively react with HMF to produce DFF. On the contrary, in the alkaline medium, the oxidation of the aldehyde is kinetically favored, obstructing the controlled oxidation of HMF to DFF. The applicability of the catalyst was further demonstrated through the selective oxidation of various biomass-derived alcohols, with yields up to 66.6% for benzaldehyde in neutral electrolyte.

Seawater is another valuable source of useful elements, including chlorine. Chlorine production from seawater offers a sustainable alternative to traditional brine-based methods, supporting the high demand in industries like water treatment and chemical manufacturing. Utilizing seawater could reduce costs and reliance on concentrated salt sources, but selective and efficient chlorine evolution remains challenging due to competition with OER. Recent developments in SACs are being investigated to improve efficiency and selectivity, making seawater a viable large-scale chlorine source for the chlor-alkali industry. In this context, Liu et al. developed a Ru-based SAC by pyrolisis, specifically designed for chlorine electrosynthesis (CER) from seawater-like solutions, overcoming the limitations of high η and low selectivity associated with the state-of-the-art catalysts.^336^ In fact, it demonstrated an exceptionally low η_10_ of only 30 mV with a chlorine selectivity of 99% and high stability over 1000 h of operation under high currents. This impressive performance results from the Ru–O_4_ coordination, lowering the Gibbs free energy for chloride adsorption and enhancing the direct formation of M–Cl intermediates, thus selectively favoring CER over OER.

In the previous section, the electrocatalytic reduction of NO_3_^−^ and NO_2_^−^ to NH_3_ was introduced as a promising alternative to the Haber–Bosch process due to the possibility of using electricity produced by renewable energy sources and reducing CO_2_ emissions. However, about 80% of the NH_3_ is used to synthesize urea, which is an important nitrogen fertilizer and chemical feedstock, through the Bosch–Meiser process. At the same time, the intensive use of fertilizers in agriculture to fulfill the increasing demand for food, as well as various industrial activities, has led to the emission of NO_3_^−^ pollutants into the environment. Therefore, at the end of the cycle, the amount of CO_2_ and NO_3_^−^ in the environment is increased. In this framework, the electrocatalytic C–N coupling between CO_2_ and NO_3_^−^ represents a promising approach to synthesizing urea and, simultaneously, mitigating CO_2_ and NO_3_^−^ pollution. Based on this motivation, Zhang et al. designed a diatomic catalyst with bonded Fe–Ni pairs on an N-doped carbon substrate (Figure 13c), enabling coordinated activation of CO_2_ and NO_3_^−^ through the synergistic action of Fe and Ni sites.^277^ Specifically, a urea yield rate of 20.2 mmol h^–1^ g^–1^ with a FE of 17.8% was obtained, outperforming the separated or mixed Ni and Fe SACs (Figure 13d). Moreover, a total FE near 100% was recorded for urea, CO, and NH_3_ as primary products, meaning that the competing HER was effectively suppressed. These results are due to FeNi–N_6_ bridge sites, promoting C–N bond formation with low activation barriers, as confirmed by numerical computations and spectroscopy analyses, which detected *NH and *CO intermediates on the Fe–Ni sites.

Similarly, Wei et al. explored the electrocatalytic synthesis of urea from CO_2_ and NO_3_^−^ using Cu SAs on CeO_2_ nanorods prepared by impregnation and calcination.^337^ Interestingly, the authors observed an unexpected yet very advantageous behavior of this SAC, since Cu atoms transitioned from the SAs state to small clusters (Cu_4_) during the reduction process to produce urea. Mechanistic studies, supported by DFT calculations, revealed that these clusters are the real active sites, facilitating the adsorption and activation of CO_2_ and NO_3_^−^ and leading to the formation of key intermediates for urea synthesis, such as *OCNO. In this way, a high urea yield rate of 52.84 mmol h^–1^ g^–1^ was obtained at an applied potential of −1.6 V vs RHE. Additionally, the reconstitution of Cu_4_ clusters to SAs was achieved upon switching to the OCP, which allows for restoring the catalyst and its original catalytic activity. The excellent structural and electrochemical stability of the catalyst was probed through eight reaction cycles, whereas CuO/CeO_2_ exhibited a rapid deterioration of its activity. Despite this work does not provide direct evidence of the electrocatalytic properties of SACs, it underscores another potential benefit of using dynamically reconstituting SAs in the catalyst design over larger and less stable NPs.

H_2_O_2_ represents another compound widely used in chemical and environmental industries, whose traditional production methods are energy-intensive and generate hazardous waste. Electrochemical two-electron ORR offers an on-site, sustainable pathway for H_2_O_2_ production. Under this motivation, Zhang et al. developed an SAC by a multistep synthesis process involving a MIL-68-In MOF as a precursor, with controlled doping of N, S, and B, followed by a carbonization process resulting in In SAs.^157^ This catalyst reached an impressive H_2_O_2_ selectivity beyond 95% across various pH levels, with a production rate of 6.49 mol g^–1^ h^–1^ in 0.1 M KOH and 6.71 mol g^–1^ h^–1^ in 0.1 M PBS with high FE, and excellent durability. Based on DFT calculations, this performance was ascribed to the unique electronic properties induced by In–N_3_SB configuration, with N and S as primary ligands and B as a secondary ligand, favoring the two-electron ORR pathway over the competing four-electron pathway that produces H_2_O. In addition, this configuration optimizes the adsorption energy for *OOH, allowing efficient H_2_O_2_ formation.

Other valuable chemicals are silanols, which are used as intermediates in the production of silicones and different chemical products. Nevertheless, conventional synthesis methods are costly and not environmentally friendly. To address these issues, a work by Tang and al introduced a sustainable and selective method for silanol synthesis from the oxidation of silanes.^338^ The researchers developed an SAC with Mn SAs on N-doped carbon, synthesized through the pyrolysis of an Mn-encapsulating ZIF-8 MOF. Under mild electrochemical conditions and with a catalyst loading as low as 600 ppm, it achieved a high turnover number of 9132, representing the number of times a single active site on the catalyst can facilitate a reaction before it becomes inactive. Its selectivity for triphenyl silane oxidation reached up to 90%, with similarly high yields of 70–85% for other silane substrates, including those containing electron-donating or electron-withdrawing groups. Of note, the catalyst retained stability and activity in a flow setup, with minimal loss in performance even under challenging reaction conditions. Overall, this study demonstrates the broad applicability of SACs, which extends also to organic electrosynthesis.

## 6 Open Questions and Limitations

Despite significant advancements, key challenges continue to hinder the full potential of SACs in electrocatalysis.^41^ We have summarized the key limitations and propose the following open questions that need to be addressed, ranging from the fundamental aspects to practical applications of SA engineering for electrocatalysis.

### 6.1 How Can Synthetic Strategies, Cost, and Commercialization Barriers Be Overcome for SACs in Electrocatalysis?

Numerous synthetic strategies have been successfully made to fabricate SACs with tailored coordination environments, increased metal loadings, and/or on a large scale. Even with these advances, there are still challenges to be addressed for the industrial application of SACs, such as mass activity, durability, and scalability. Although the atomic activity of SACs overwhelms the NP-based catalysts in most of the electrocatalytic reactions, it does not guarantee better mass activity due to the low SA metal loadings. Several successful cases of carbon-based SACs using pyrolysis were reported for high metal loadings. Also, the durability of SACs should be further enhanced for the practical application. Only a few strategies, such as thermal treatment or application of mechanical forces, were reported to improve the durability. The synthetic strategies to make the SACs intact during the reactions should be further developed. Lastly, large-scale synthesis is crucial for industrial applications. Kilogram synthesis of SACs could be achieved by ball-milling, showing it as a promising method for the scaled-up synthesis of SACs. Besides, further advances should be made to prepare SACs with desired ligand environments and supports. Meanwhile, an intrinsic limitation of SACs is the absence of ensemble sites, which are required to catalyze some surface reactions, such as bond scissoring or creating, or the reactions with multiple reactants.^340^ This can be overcome by the adoption of dual- or triple-atom catalysts which provide additional binding sites to cleave or create bonds.^341^ Currently, the cost of commercially available SACs remains prohibitively high, posing a significant barrier to their widespread adoption in industrial applications. The expense of producing SACs, particularly those with precise atomic dispersion and tailored coordination environments, far exceeds that of benchmark catalysts like Pt/C. Addressing this cost disparity is essential for transitioning SACs from research to commercial-scale applications.

### 6.2 How Can In Situ Characterization under Reaction Conditions Improve the Understanding of SACs?

Characterizing SACs for electrocatalysis presents several challenges that must be addressed to optimize their properties for practical applications. A key issue is developing more sensitive and accurate techniques to detect and characterize isolated single atoms, especially in the presence of complex support. Current methods, like electron microscopy and XAS, often struggle with weak signals and diverse coordination environments. In situ and operando techniques are crucial for observing SACs under reaction conditions, yet these methods face difficulties in tracking dynamic changes in atomic structure, oxidation states, and coordination due to the low concentration of active sites and instability under reaction environments. Future research should focus on advancing these techniques to provide real-time, detailed insights into SAC behavior during catalysis. The integration of multiple characterization methods, such as EPR, FTIR, and XPS, is essential for overcoming the limitations of individual techniques and achieving a more comprehensive understanding of SACs’ performance and mechanisms during the electrocatalysis process.

### 6.3 How Can Computational Methods and Machine Learning Enhance Mechanistic Insights into SACs?

Gaining mechanistic insights into SACs requires a comprehensive exploration of the chemical configurations of SAC active sites. This includes variations in the oxidation states and spin states of the anchored metals, as well as the arrangement of their anchoring sites.^192,197^ This exploration calls for computational methods that are both efficient and reliable in capturing reaction profiles since conventional approaches often demand extensive computational resources. Fortunately, the increasing computational power of modern computers has facilitated more accurate simulations, allowing researchers to explore complex catalytic environments and narrow the gap between theoretical models and practical applications. Simultaneously, the rise of semiempirical methods and ML-based potentials has revolutionized large-scale screening of potential SACs, significantly accelerating the discovery of new materials. However, these approaches are not without challenges, because ML models often suffer from limited interpretability and depend on extensive, high-quality training data sets that are still largely based on DFT calculations, inheriting their intrinsic limitations.

An underexplored yet vital aspect is the interplay between the catalytic activity and stability, which involves identifying the most active and stable configurations.^190,197^ This understanding is crucial for guiding experimentalists in designing more efficient SACs. Additionally, the dynamic changes that SACs may undergo during catalysis must be considered, necessitating methods that can describe dynamics during catalytic processes.^193,194^ While many tools of theoretical chemistry have been developed to describe catalytic processes involving inorganic homogeneous and heterogeneous catalysts, as well as enzymes, the complexity of SACs calls for even more sophisticated tools and workflows.

### 6.4 How Can We Optimize Active Site Density, Stability, and Performance of SACs in Practical Applications?

Regarding SACs’ limitations in practical applications, one primary challenge lies in optimizing the density and accessibility of active sites. Current methods struggle to balance the atomic dispersion of metals with maximizing the number of active sites available for electrochemical reactions. This limitation becomes particularly significant in industrial-scale applications, where achieving high current densities is essential. Another critical question is related to the mechanistic understanding of multielectron processes, such as CO_2_RR, where the competition between multiple reaction pathways often leads to low selectivity and efficiency. Although significant progress has been made in tailoring the coordination environment of SACs to influence product selectivity, the dynamic behavior of the active sites under operating conditions remains poorly understood.^342^

Furthermore, the stability of SACs under prolonged electrochemical conditions presents a significant challenge. In addition to the agglomeration of the active sites, in reactions like water splitting, ORR, CO_2_RR, and NRR, SACs face degradation mechanisms such as corrosion and atom detachment from the support, especially under high potentials or acidic/alkaline environments. Understanding and mitigating these degradation pathways is crucial for industrial use. Another under-explored issue is the influence of electrolyte composition and interface effects on the performance of SACs. Despite electrolyte ions and relatively small fluctuations in pH can dramatically affect the adsorption energies of intermediates, altering both activity and selectivity, the precise mechanisms driving these effects remain ambiguous. Finally, the role of mass transport limitations in SACs has yet to be systematically explored. The tailored engineering of SACs using porous architectures with enhanced reactant accessibility and product desorption characteristics could unlock new performance thresholds.^343^

## 7 Conclusions

SACs embody a groundbreaking evolution in the field of electrocatalysis, combining maximum atomic efficiency with tunable catalytic properties. By leveraging the full exposure of atomic active sites and tailoring electronic structures through precise anchoring, SACs address two critical challenges of conventional electrocatalysts: the high cost of noble metals and the limited efficiency of nonprecious materials. The insights gathered in this review highlight the transformative implications of SACs, not only for fundamental catalytic science but also for the future of renewable and sustainable energy solutions.

The success of SACs lies in their ability to uniquely merge the strengths of homogeneous and heterogeneous catalysis. The isolated active sites provide uniform and well-defined reaction environments, bridging the gap between the two paradigms. By engineering metal–support interactions, SACs achieve unparalleled control over activity, selectivity, and stability. Advanced bottom-up and top-down synthetic methods have enabled the anchoring of metal SAs on various supports, including materials with large surface areas or efficient charge transport properties, which further contribute to the catalytic efficiency. However, a critical achievement in SAC research is the ability to precisely stabilize these SAs at high metal loadings, overcoming their natural tendency to aggregate due to high surface free energy. Microscopy and spectroscopy characterization techniques with atomic resolution, such as HAADF-STEM and XAS, have been crucial for studying SACs, with in situ/operando techniques enabling the observation of SAC dynamics under realistic conditions. Parallely, theoretical calculations have proved to be vital in modeling the catalytic properties of SACs, guiding the rational design of catalysts and providing a higher degree of comprehension of the experimental results.

SACs have outperformed conventional nanostructured electrocatalysts, as indicated by superior performance metrics such as lower overpotential and Tafel slope values, and higher TOF and mass activity. A common denominator of these indicators is the better utilization of the active sites. This supremacy is evident across a range of electrocatalytic reactions, and, in this review, the most recent and relevant strategies that advanced OER and HER, ORR, CO_2_RR, and NH_3_ synthesis have been presented. In particular, by properly engineering SAs, it has been possible to adjust the adsorption/desorption energy of reaction intermediates, favoring a specific reaction pathway over competing reactions. In addition, the synergistic effect between different SA metal sites or neighboring NCs has shown to be an efficient strategy to boost the performance of SACs. Besides, in recent years, they have demonstrated considerable improvements in other relevant electrocatalytic processes for natural resource recovery, waste-to-value conversion, and organic synthesis, with many more interesting reactions that have not been explored yet.

Despite these advances, to fully exploit the potential of SACs, researchers must deepen their understanding of reaction mechanisms under operational conditions. This will require the integration of operando spectroscopic and microscopic methods to capture the dynamic behavior of catalysts during reactions. Additionally, computational modeling, augmented by ML, can accelerate the discovery of novel SACs, optimizing their properties for specific reactions. Another promising avenue is the integration of SACs into hybrid systems, such as photoelectrocatalytic setups, which could revolutionize renewable energy production by combining photo- and electrocatalysis in a single platform. These processes have started to be investigated but are still less explored compared to electrocatalytic reactions and may unlock new solutions for green fuel generation and recovery/production of valuable chemical substances. Sustainability will remain a core priority in SAC development. Efforts to utilize abundant and inexpensive metals, coupled with eco-friendly and affordable synthesis methods, will enhance the environmental and economic viability of SACs. The journey ahead will require overcoming existing limitations through interdisciplinary collaboration, blending the best of materials science, chemistry, and engineering. As SACs transition from laboratory studies to industrial applications, they have the potential to play a pivotal role in shaping a cleaner and more sustainable future.
